# Sex-specific trajectories of molecular cardiometabolic traits from childhood to young adulthood

**DOI:** 10.1136/heartjnl-2022-321347

**Published:** 2023-03-13

**Authors:** Linda M O'Keeffe, Kate Tilling, Joshua A Bell, Patrick T Walsh, Matthew A Lee, Deborah A Lawlor, George Davey Smith, Patricia M Kearney

**Affiliations:** 1 School of Public Health, University College Cork, Cork, Ireland; 2 MRC Integrative Epidemiology Unit at the University of Bristol, University of Bristol, Bristol, UK; 3 Population Health Sciences, Bristol Medical School, University of Bristol, Bristol, UK

**Keywords:** Metabolic Diseases

## Abstract

**Background:**

The changes which typically occur in molecular causal risk factors and predictive biomarkers for cardiometabolic diseases across early life are not well characterised.

**Methods:**

We quantified sex-specific trajectories of 148 metabolic trait concentrations including various lipoprotein subclasses from age 7 years to 25 years. Data were from 7065 to 7626 offspring (11 702 to14 797 repeated measures) of the Avon Longitudinal Study of Parents and Children birth cohort study. Outcomes were quantified using nuclear magnetic resonance spectroscopy at 7, 15, 18 and 25 years. Sex-specific trajectories of each trait were modelled using linear spline multilevel models.

**Results:**

Females had higher very-low-density lipoprotein (VLDL) particle concentrations at 7 years. VLDL particle concentrations decreased from 7 years to 25 years with larger decreases in females, leading to lower VLDL particle concentrations at 25 years in females. For example, females had a 0.25 SD (95% CI 0.20 to 0.31) higher small VLDL particle concentration at 7 years; mean levels decreased by 0.06 SDs (95% CI −0.01 to 0.13) in males and 0.85 SDs (95% CI 0.79 to 0.90) in females from 7 years to 25 years, leading to 0.42 SDs (95% CI 0.35 to 0.48) lower small VLDL particle concentrations in females at 25 years. Females had lower high-density lipoprotein (HDL) particle concentrations at 7 years. HDL particle concentrations increased from 7 years to 25 years with larger increases among females leading to higher HDL particle concentrations in females at 25 years.

**Conclusion:**

Childhood and adolescence are important periods for the emergence of sex differences in atherogenic lipids and predictive biomarkers for cardiometabolic disease, mostly to the detriment of males.

**WHAT IS ALREADY KNOWN ON THIS TOPIC**

- Causal risk factors and novel predictive biomarkers for cardiometabolic diseases are increasingly being identified from the recent application of comprehensive metabolomic profiling in epidemiological studies, but the changes which typically occur in these traits across childhood, adolescence and early adulthood are not well understood.

**WHAT THIS STUDY ADDS**

- In this prospective cohort study with repeat assessments of 148 molecular cardiometabolic traits from comprehensive metabolomic profiling at ages 7, 15, 18 and 25 years, we demonstrate that marked changes in levels of causal risk factors and novel predictive biomarkers for cardiometabolic diseases occur from childhood to early adulthood. These findings suggest that childhood and adolescence are important life stages for the development of sex differences in atherogenic lipids and predictive biomarkers for cardiometabolic disease, mostly to the detriment of males.

**HOW THIS STUDY MIGHT AFFECT RESEARCH, PRACTICE OR POLICY**

- Findings suggest that childhood and adolescence should be targeted for prevention of cardiometabolic disease and sex differences in cardiometabolic risk across the life course

## Introduction

Cardiometabolic diseases are a leading cause of death globally.^1^ Cardiometabolic risk factors such as adiposity, blood pressure and circulating lipids, as well as underlying subclinical artery disease likely originate in early life, potentially beginning in childhood and tracking through adolescence into adulthood.^2^ Understanding how risk factors begin, change and track from childhood to adulthood is important for informing aetiological understanding of cardiometabolic diseases and identifying groups in need of targeted prevention.

To date, longitudinal studies have characterised how conventional cardiometabolic risk factors change over time from childhood to adulthood, including studies of change in adiposity,^3–5^ blood pressure,^3–6^ and circulating lipids (triglycerides, high-density lipoprotein (HDL) and non-HDL,^3 4 7^ as well as glucose and insulin.^3 5 8^ In a previous study of the Avon Longitudinal Study of Parents and Children (ALSPAC), we demonstrated distinct patterns of change in conventional cardiometabolic risk factors through the first decades of life, with change for some risk factors coinciding with the sensitive period of puberty.^9^ We also demonstrated notable sex differences in circulating lipids that began at birth, including higher HDL and non-HDL among females, which widened further by age 18 years.^3 5^ While these studies provide important insights into early life course development and change in a small number of cardiometabolic risk factors, few studies to date have characterised change in molecular cardiometabolic traits from metabolomics platforms which are now being used for more granular and high-resolution cardiovascular phenotyping to better understand cardiometabolic disease aetiology.^10–12^ These platforms include directly measured apolipoprotein B containing lipoprotein subclasses that cause atherosclerotic plaques and lead to coronary heart disease (CHD) and could only previously be indirectly estimated using conventional approaches.^13^ As yet, understanding of how these traits typically begin and change from childhood through to adulthood using population-based/non-clinical samples is lacking, despite potential to provide refined understanding of cardiometabolic disease aetiology. In addition, characterising the sex-specific development of these traits is important due to striking sex differences in cardiometabolic disease risk across the life course, which remain poorly understood. The study of sex differences in cardiometabolic health is now widely recognised as an area of unlocked potential for informing improved aetiological understanding and mobilising more effective future prevention opportunities.^14^ In particular, the study of sex-specific trajectories from early in the life course through to adulthood has potential to reveal important mechanisms/mediators of these sex differences for future study, which are likely to be complex and multifactorial and driven by both biological (sex hormones, genetics and epigenetics) and social factors (such as health behaviours, adiposity or health service use). For instance, sex differences arising very early in childhood are likely to be mediated by biological factors such as genetics or perhaps early social differences but would be less likely to be mediated by health behaviours such as smoking and alcohol use, which begin in adolescence. In contrast, sex differences that emerge or widen during the sensitive period of adolescence may suggest that both biological factors (sex hormones and puberty timing) and/or perhaps sex differences in the onset and prevalence of health behaviours such as smoking and alcohol use play a role. In addition, studying sex differences in trajectories of absolute levels of key cardiometabolic traits in early life provides opportunity to better understand whether sex differences are driven by this or differences in the relative association of risk factors with cardiometabolic risk, which has been a key focus of much research to date.^15^

Using data from ALSPAC, a large contemporary birth cohort study from South west England, we examined sex-specific trajectories of 148 concentrations of molecular cardiometabolic traits, mostly lipoprotein subclasses and fatty acids, but also including glucose and an inflammatory marker, measured using targeted metabolomics on 4 occasions among the same individuals, from 7 years to 25 years.

## Methods

### Study population

The ALSPAC is a prospective birth cohort study in South west England. Pregnant women resident in Avon, UK with expected dates of delivery 1st April 1991 to 31st December 1992 were invited to take part in the study. The initial number of pregnancies enrolled was 14 541. Of these, there was a total of 14 676 fetuses, resulting in 14 062 live births and 13 988 children who were alive at 1 year. Follow-up has included parent and offspring completed questionnaires, links to routine data and clinic attendance. The study has been described elsewhere in detail.^16–18^ The study website contains details of all the data that are available through a fully searchable data dictionary (http://www.bristol.ac.uk/alspac/researchers/our-data/). Study data were collected and managed using Research Electronic Data Capture (REDCap) electronic data capture tools hosted at the University of Bristol.^19^ REDCap is a secure, web-based software platform designed to support data capture for research studies.

### Patient and public involvement

This analysis was performed without patient or public involvement.

### Assessment of cardiometabolic traits

Proton nuclear magnetic resonance (NMR) spectroscopy from a targeted metabolomics platform^20^ was performed on EDTA plasma samples from blood samples drawn in clinics at ages 7, 15, 18 and 25 years to quantify 148 concentrations including cholesterol, triglyceride, and other lipid content in lipoprotein subclass particles, apolipoproteins, fatty acids, glucose and an inflammatory marker (glycoprotein acetyls). Four traits were not measured at 25 years (diacylglycerol, fatty acid chain length, estimated degree of unsaturation and conjugated linoleic acid). Blood was taken after a minimum of a 6-hour fast (stability in these trait concentrations has been shown over different fasting durations).^21^ Laboratory NMR quality control and further data preparation steps are described in online supplemental appendix S1).

10.1136/heartjnl-2022-321347.supp1 Supplementary data

### Statistical analysis

We used multilevel models to examine sex-specific patterns of change in 148 trait concentrations; 144 traits had measures available from 7 years to 25 years, while 4 traits had measures available from 7 years to 18 years^22^; thus, for these traits, change over time is only modelled to age 18 years. Multilevel models estimate mean trajectories of the outcome while accounting for the non-independence (ie, clustering) of repeated measurements within individuals and differences in the number and timing of measurements between individuals (using all available data from all eligible participants under a missing at random assumption).^23 24^ For inclusion in the present analysis, participants required data on sex and at least 1 measure of a metabolic trait between 7 years and 25 years (144 traits) and between 7 years and 18 years (4 traits). Given that sex is the exposure in these analyses, we did not adjust for potential confounders because sex cannot be altered by factors that influence the metabolic traits (outcomes). For example, body mass index is likely to influence many of the metabolic traits, but it could not change participant sex and therefore could not confound the effect of sex on metabolic trait levels (although it might mediate such effects). Change in all 148 traits was estimated here using linear spline multilevel models (2 levels: measurement occasion and individual). Linear splines allow knot points to be fit at different ages to derive periods in which change is approximately linear. All trajectories were modelled in MLwiN V.3.04, called from Stata V.15^25^ using the runmlwin command.^26^ Data visualisation was performed using R V.3.6.3 using the ggforestplot V.0.0.2 package. Further information on the traits included and our modelling strategy is included in online supplemental appendix S2 and table S1. We also performed several sensitivity analyses and details of these are included in online supplemental appendix S3 and table S2.

## Results

**Figure 1:**
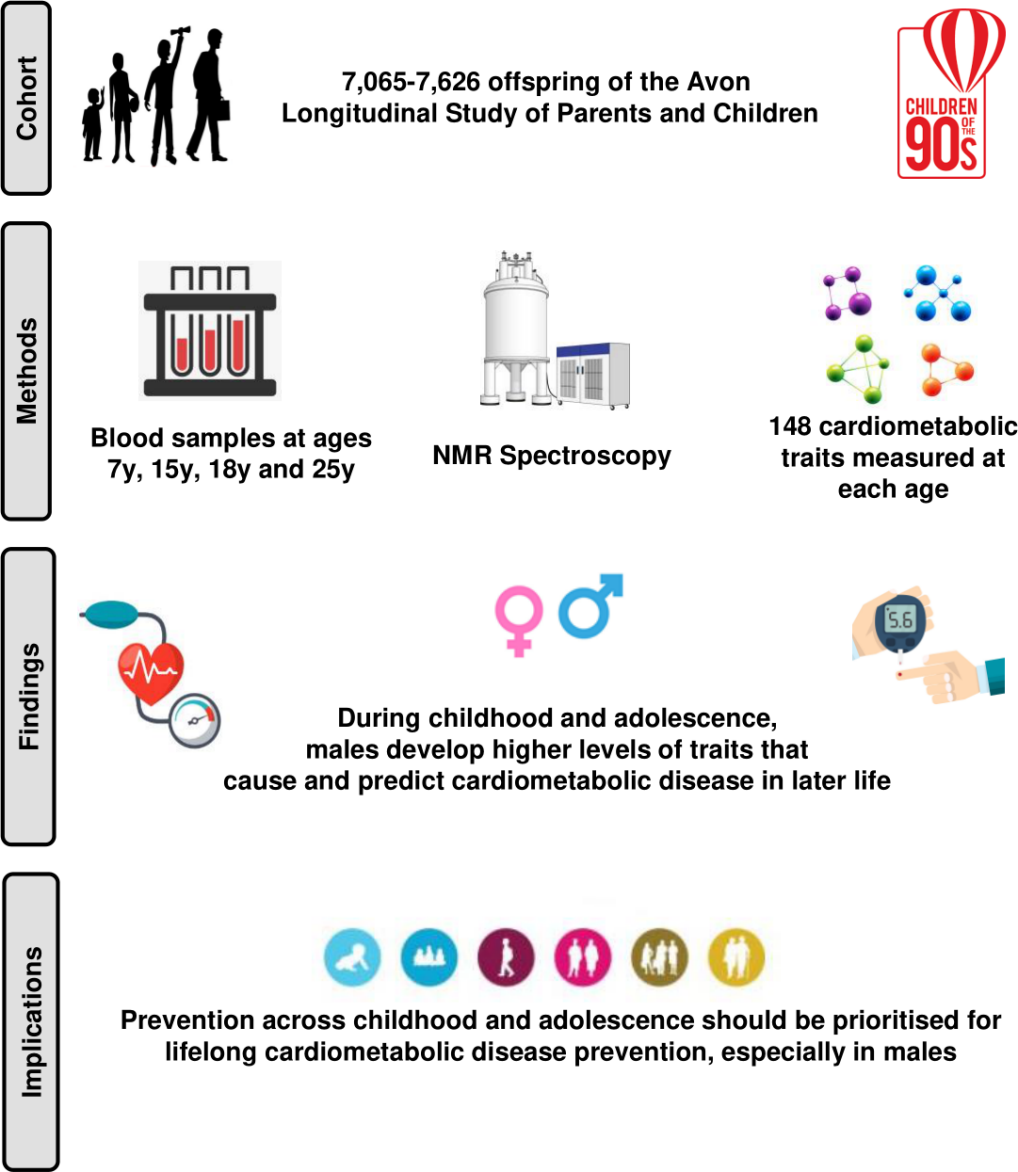
Summary of methods and findings of study. Legend: NMR, nuclear magnetic resonance; y, years.

Figure 1 provides an overall summary of our study and its findings. The characteristics of participants included in analyses by sex (49% male) are shown in table 1. A total of 148 metabolic trait concentrations were modelled. A total of 7626 participants (14 797 total repeated measures; 5444 at 7 years, 3048 at 15 years, 3121 at 18 years and 3184 at 25 years) were included in the analyses of 144 concentrations. A total of 7065 participants (11 702 total repeated measures; 5395 at 7 years, 3219 at 15 years and 3088 at 18 years) were included in analyses of diacylglycerol, fatty acid chain length, estimated degree of saturation and conjugated linoleic acid as these traits were not available at 25 years.

**Table 1:** Characteristics of Avon Longitudinal Study of Parents and Children participants included in the analysis, by sex

	Females n=3909*	Males n=3717*
	n (%)	n (%)
Non-white ethnicity	75 (2.2)	60 (1.8)
Maternal marital status		
Never married	527 (15.1)	467 (13.7)
Widowed	<5	8 (0.2)
Divorced	110 (3.2)	118 (3.5)
Separated	50 (1.4)	36 (1.1)
1st marriage	2581 (73.9)	2544 (74.8)
Marriage 2 or 3	224 (6.4)	229 (6.7)
Household social class†		
Professional	517 (15.8)	532 (16.7)
Managerial and technical	1463 (44.6)	1404 (44.1)
Non-manual	793 (24.2)	792 (24.9)
Manual	343 (10.5)	328 (10.3)
Part skilled and unskilled	161 (4.9)	128 (4.0)
Maternal education		
Less than O level	761 (22.2)	724 (21.6)
O level	1166 (34.1)	1195 (35.7)
A level	926 (27.1)	883 (26.4)
Degree or above	569 (16.6)	548 (16.4)
Mother’s partner’s highest educational qualification		
Less than O level	965 (28.9)	863 (26.5)
O level	717 (21.5)	724 (22.2)
A level	916 (27.5)	918 (28.2)
Degree or above	737 (22.1)	750 (23.0)
Maternal smoking during pregnancy	660 (18.9)	657 (19.2)
	Mean (SD)	Mean (SD)
Gestational age (weeks)	39.6 (1.8)	39.3 (1.9)
Birth weight (g)	3370 (512)	3469 (578)
Maternal age (years)	28.8 (4.6)	29.1 (4.7)
Maternal prepregnancy body mass index (kg/m^2^)	22.8 (3.6)	22.9 (3.8)

*Note that denominators in this table do not sum exactly to N participants included in models due to some missing covariate data, which were not required for inclusion in our analyses.

†Household social class was measured as the highest of the mother’s or her partner’s occupational social class using data on job title and details of occupation collected about the mother and her partner from the mother’s questionnaire at 32 weeks’ gestation. Social class was derived using the standard occupational classification codes developed by the UK Office of Population Census and Surveys and classified as I, professional; II, managerial and technical; IIINM, non-manual; IIIM, manual; and IV&V, part skilled occupations and unskilled occupations.

### Very-low-density lipoprotein (VLDL) concentrations

**Figure 2:**
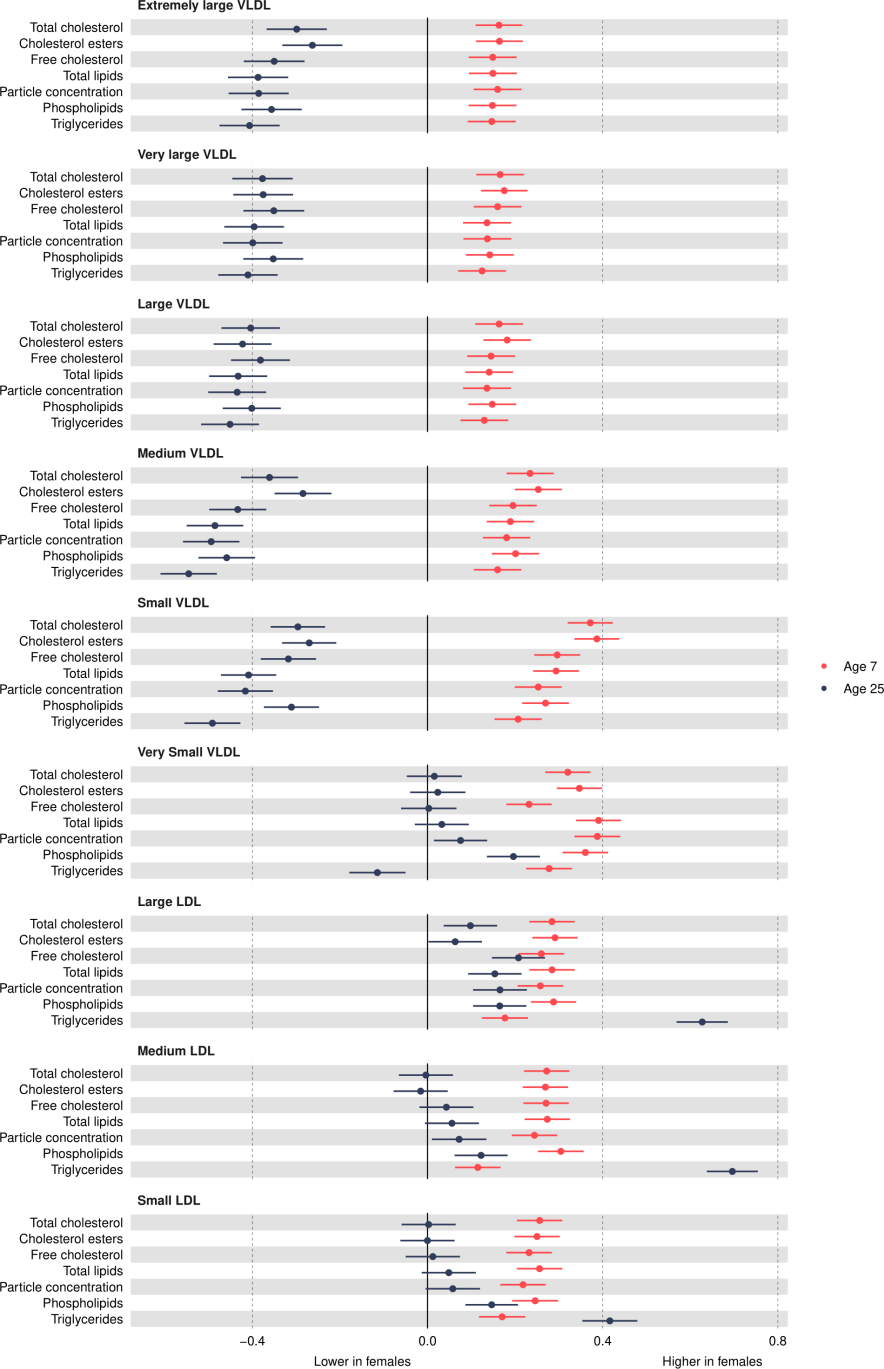
Mean sex difference in VLDL and LDL lipoprotein concentrations in SD units at 7 and 25 years, estimated from multilevel models. Differences shown are for females compared with males. LDL, low-density lipoprotein; VLDL, very-low-density lipoprotein.

**Figure 3:**
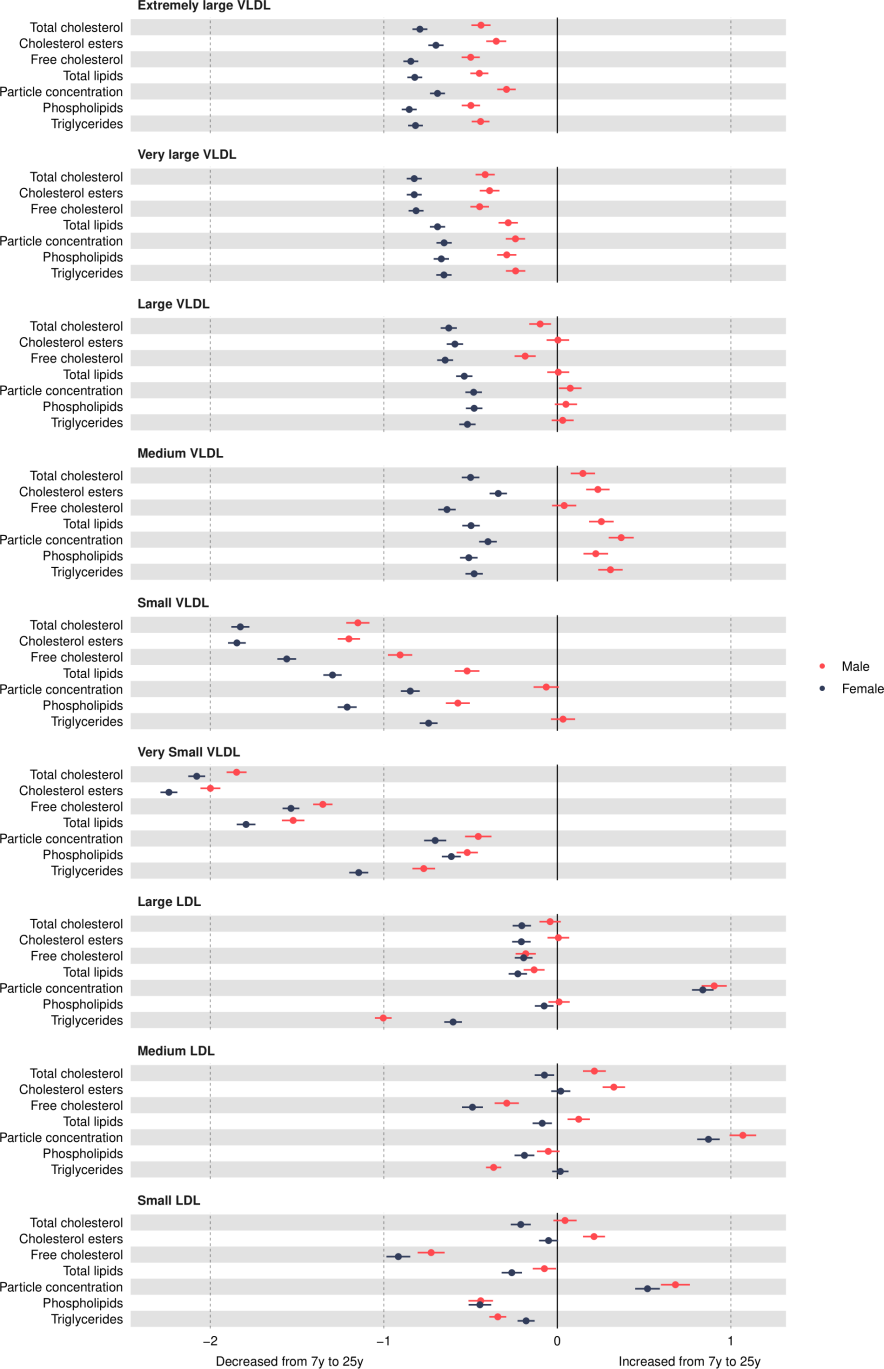
Mean sex-specific change in VLDL and LDL lipid concentrations in SD units from 7 to 25 years, estimated from multilevel models. LDL, low-density lipoprotein; VLDL, very-low-density lipoprotein.

VLDL particle concentrations were higher in females at 7 years, e.g., 0.39 SD (95% CI, 0.34, 0.44) higher for very small VLDL (figure 2 and online supplemental table S3). Except for large and medium concentrations among males which increased over time, most other VLDL particle concentrations decreased from 7 years to 25 years in both sexes (figure 3 and online supplemental table S4), and females had larger decreases compared with males. At 25 years, females had lower levels of most VLDL particle concentrations, except for very small VLDL particle concentrations which remained 0.08 SD (95% CI 0.01 to 0.14) higher in females, although the difference had reduced in magnitude. Patterns were broadly similar for lipid content in VLDL particles over time.

### Low-density lipoprotein (LDL) concentrations

Most LDL particle concentrations were higher in females at 7 years (eg, 0.26 SD (95% CI 0.21 to 0.31) higher for large LDL) (figure 2 and online supplemental table S3). LDL particle concentrations increased from 7 years to 25 years (figure 3 and online supplemental table S4). At 25 years, higher levels of LDL particle concentrations persisted in females, for example, 0.17 SD (95% CI, 0.1, 0.23) higher for large LDL but the difference had reduced in magnitude due to smaller increases in females compared with males. These patterns were similar for lipid content in LDL particles, except for triglycerides in LDL; females had higher levels of triglycerides in LDL at 7 years, and this difference widened at 25 years in contrast to a reduction in the difference for other lipid particles in LDL. This reduction appeared to be driven by smaller decreases in triglyceride in LDL particles over time in females.

### HDL concentrations

**Figure 4:**
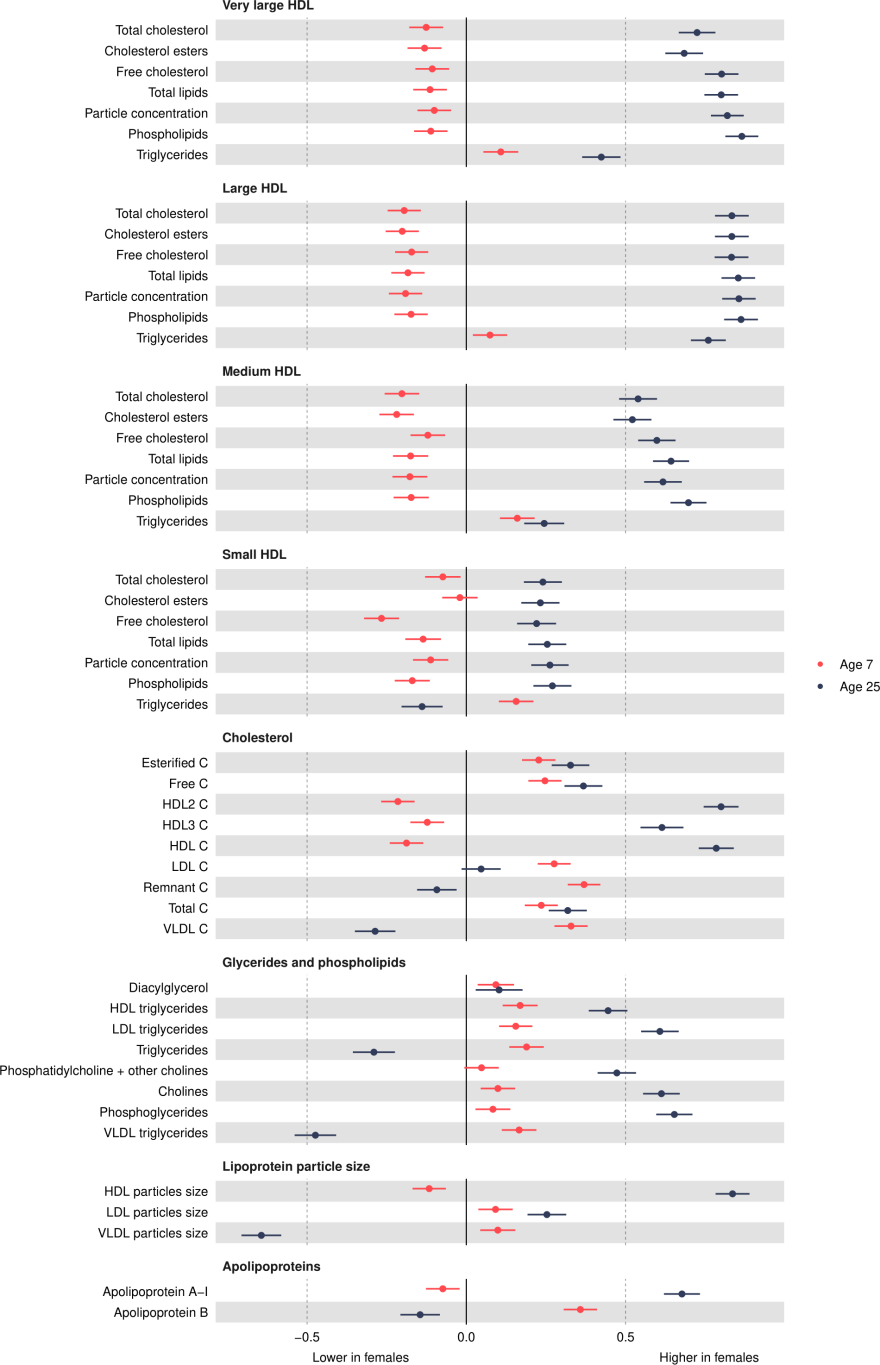
Mean sex difference in lipid concentrations in SD units at 7 and 25 years, estimated from multilevel models. Note that diacylglycerol is measured only up to 18 years. Differences shown are for females compared with males. HDL, high-density lipoprotein; LDL, low-density lipoprotein;VLDL, very-low-density lipoprotein.

**Figure 5:**
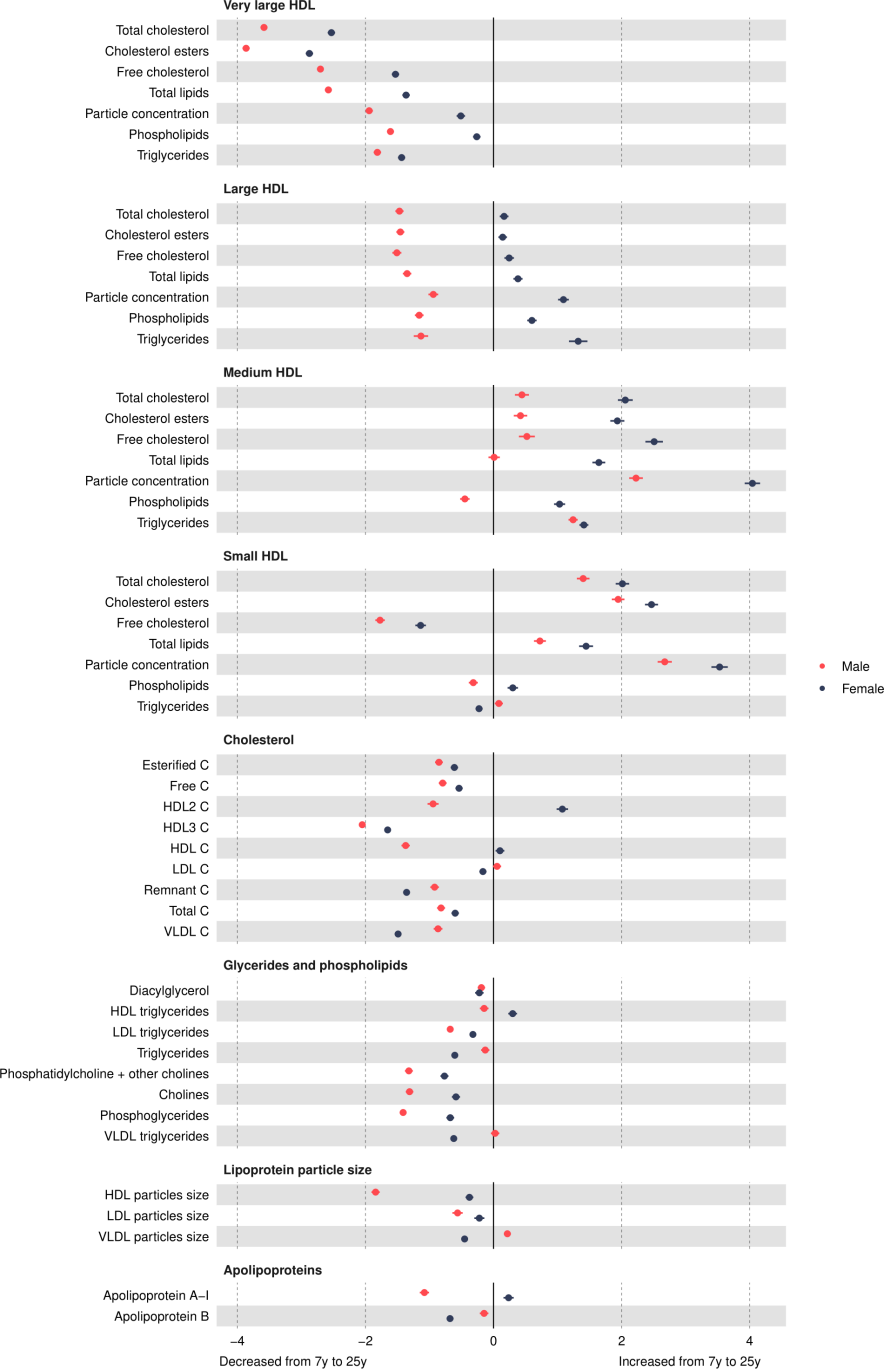
Mean sex-specific change in lipid concentrations in SD units from 7 to 25 years, estimated from multilevel models. Note that diacylglycerol is measured only up to 18 years. HDL, high-density lipoprotein; LDL, low-density lipoprotein; VLDL, very-low-density lipoprotein.

Most HDL particle concentrations were lower in females at age 7 years (eg, −0.1 SD (95% CI, −0.15 to –0.05) lower for very large HDL (figure 4 and online supplemental table S3). Very large HDL particle concentrations decreased from 7 years to 25 years in both sexes and females had smaller decreases than males (figure 5 and online supplemental table S4). Large HDL particle concentrations increased from 7 years to 25 years in females but decreased in males. Medium and small HDL particle concentrations increased from 7 years to 25 years in both sexes with larger increases in females. At 25 years, females had higher levels of all HDL particle concentrations, for example, 0.82 SD (95% CI 0.77 to 0.87) higher for very large HDL. Patterns were similar for different lipid contents in HDL particles.

### Cholesterol

HDL cholesterol concentrations were lower in females at 7 years (figure 4 and online supplemental table S3). All other cholesterol concentrations were higher in females at 7 years. Most cholesterol concentrations decreased from 7 years to 25 years except for HDL and HDL2 in females and LDL in males, which increased over time (figure 5 and online supplemental table S4). Females had smaller decreases in esterified, free, HDL3 and total cholesterol concentrations and larger decreases in VLDL and remnant cholesterol concentrations. At 25 years, females had higher esterified, free, total and HDL cholesterol concentrations and lower VLDL and remnant cholesterol concentrations, though levels of LDL cholesterol concentrations were similar between the sexes.

### Glycerides and phospholipids

All glyceride and phospholipid concentrations were higher in females at 7 years (figure 4 and online supplemental table S3). Concentrations decreased over time in both sexes except for HDL triglyceride concentrations in females which increased over time and VLDL triglycerides which did not change between 7 years and 25 years in males (figure 5 and online supplemental table S4). Females had smaller decreases for most traits, except for total triglyceride concentrations which had a larger decrease. At 25 years, females had higher levels of most glyceride and phospholipid concentrations except total and VLDL triglycerides, which were lower in females.

### Particle size and apolipoproteins

LDL and VLDL particle sizes were larger in females at 7 years, but HDL particle size was smaller (figure 4 and online supplemental table S3). HDL and LDL particle sizes decreased in both sexes from 7 years to 25 years, and this decrease was smaller in females (figure 5 and online supplemental table S4). In contrast, VLDL particle size decreased in females and increased in males between 7 years and 25 years. At 25 years, LDL and HDL particle sizes were larger in females, while VLDL particle size was smaller. Apolipoprotein B was higher in females at 7 years and decreased from 7 years to 25 years in both sexes, but females had larger decreases. Apolipoprotein B was lower in females at 25 years. Apolipoprotein A-1 was lower in females at 7 years and increased over time in females but decreased over time in males. Apolipoprotein A-1 was higher in females at 25 years.

### Other non-lipid traits

**Figure 6:**
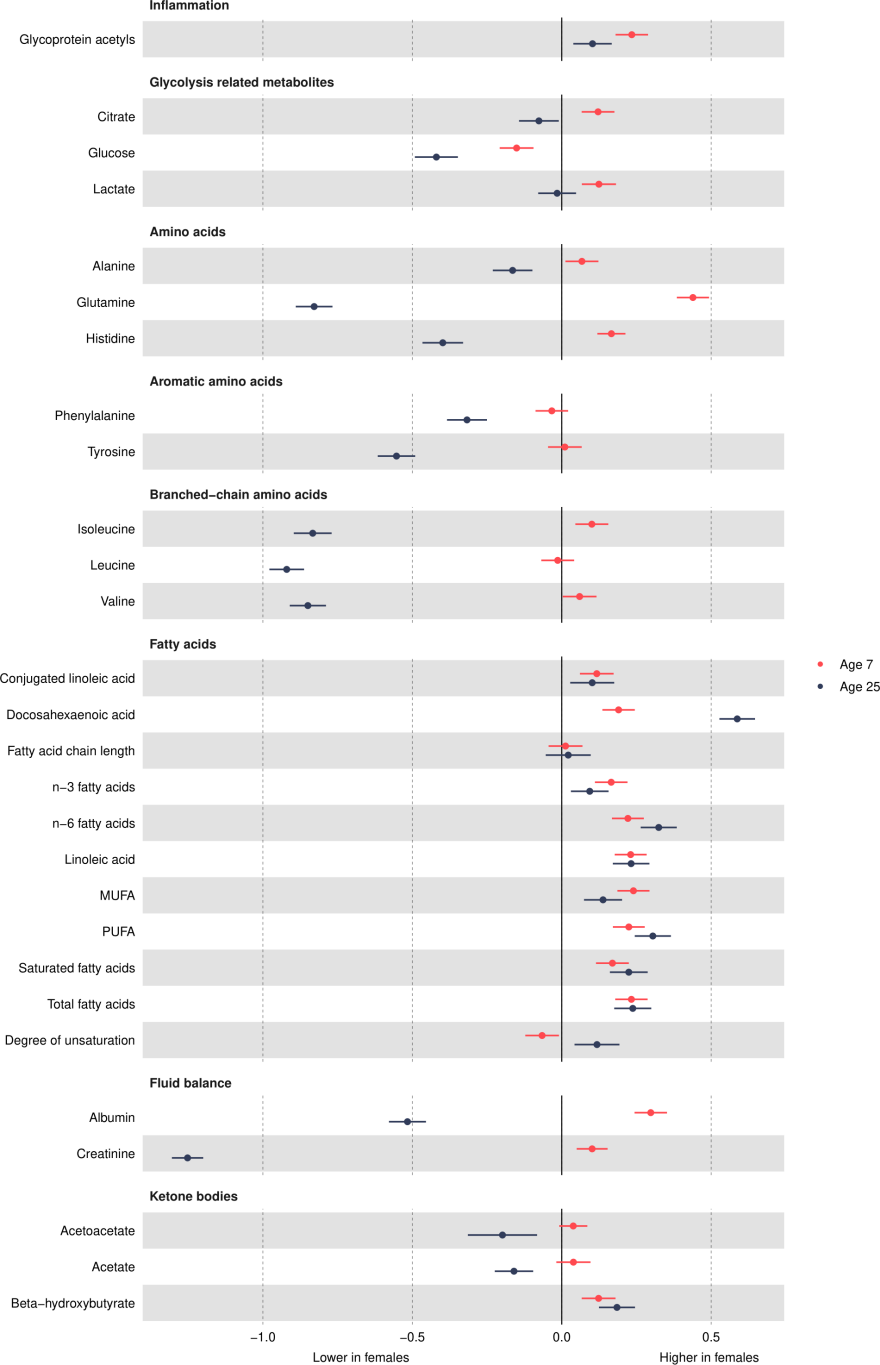
Mean sex difference in other trait concentrations in SD units at 7 and 25 years, estimated from multilevel models. Differences shown are for females compared with males. Note that conjugated linoleic acid, fatty acid chain length and estimated degree of unsaturation are measured only up to 18 years. MUFA, monounsaturated fatty acid; PUFA, polyunsaturated fatty acid.

**Figure 7:**
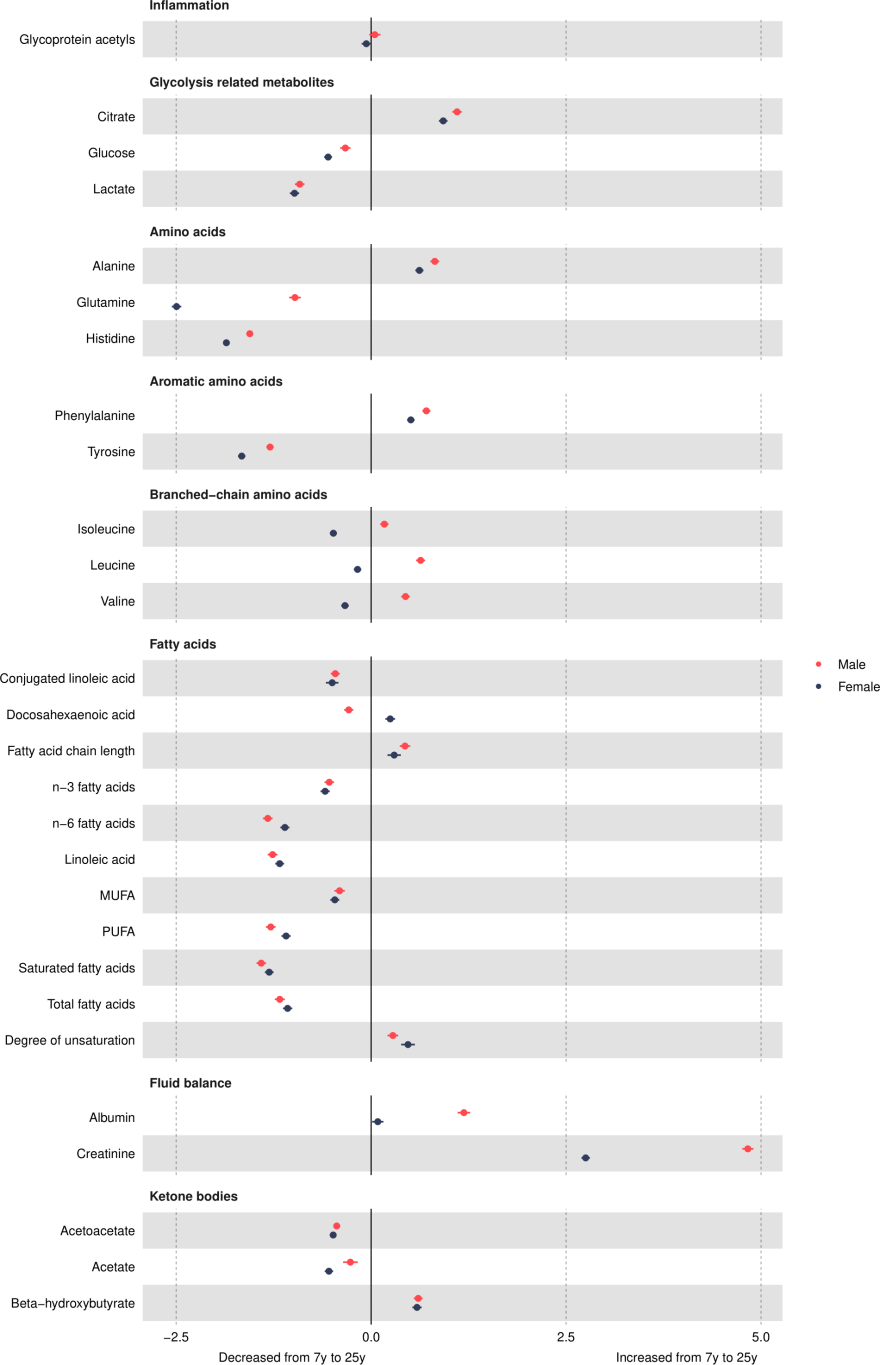
Mean sex-specific change in other trait concentrations in SD units from 7 to 25 years, estimated from multilevel models. Note that conjugated linoleic acid, fatty acid chain length and estimated degree of unsaturation are measured only up to 18 years. MUFA, monounsaturated fatty acid; PUFA, polyunsaturated fatty acid.

Glycoprotein acetyls was higher in females at 7 years (figure 6 and online supplemental table S3) and decreased in females and increased in males from 7 years to 25 years (figure 7 and online supplemental table S4), such that concentrations remained higher in females at 25 years, with a smaller difference than that observed at age 7 years. Citrate and lactate were higher in females at 7 years and lower (citrate) in females or similar between the sexes (lactate) at 25 years. Glucose was lower in females at 7 years, and this difference widened at 25 years, driven by larger decreases in females from 7 years to 25 years compared with males. All amino acids were higher in females at 7 years or similar between the sexes. Most amino acid concentrations decreased over time, except for alanine and phenylalanine in both sexes and branched chain amino acids in males, which increased over time. At 25 years, all amino acids were lower in females. Except for docosahexaenoic acid and degree of unsaturation, all fatty acids were higher in females at 7 years, and this difference was similar at 25 years due to similar changes from 7 years to 25 years in females and males (all decreased except for fatty acid chain length, which increased over time). Docosahexaenoic acid was higher in females at 7 years, and this difference widened over time. Degree of unsaturation was lower in females at 7 years and higher in females at 18 years (note, 1 of 4 traits only measured to 18 years). Albumin and creatinine were higher in females at 7 years and lower in females at 25 years due to smaller increases in females from 7 years to 25 years. Ketone bodies such as acetoacetate were higher in females at 7 years; this difference was similar at 25 years for beta-hydroxybutyrate but acetoacetate and acetate were lower in females at 25 years.

### Sensitivity and additional analyses

Mean rates of change in original units in each linear spline period are shown in online supplemental table S5. Sex differences in each trait at 7 years and 25 years estimated from multilevel models were very similar to those obtained from linear regression (online supplemental table S6). Our findings were also very similar in analyses weighted by the probability of being included in analyses (online supplemental figures S1–S3) and when repeated standardising by the sex-specific mean and SD at 7 years (online supplemental figures S4–S6).

## Discussion

In this prospective UK birth cohort study, we examined sex-specific trajectories of 148 molecular traits from a targeted metabolomics platform, each measured repeatedly from childhood to early adulthood. Overall, our findings suggest that childhood and adolescence are important periods for the emergence of sex differences in atherogenic lipids (apolipoprotein B containing VLDL and LDL traits) and predictive biomarkers (glucose and HDL) for cardiometabolic diseases, mostly to the detriment of males.

CHD risk tends to appear higher in males until mid-life, after which risk becomes more similar between females and males.^27^ In another study in the parents of participants in this cohort, absolute levels of a number of traits studied here (such as small VLDL) were shown to change little from 25 years to 50 years,^28^ suggesting that differences found here may track into mid-life. In addition, recent evidence from a Mendelian randomisation (MR) study^11^ of the causal effect of these traits on CHD risk in adults suggests our findings are likely to be clinically meaningful in the long term. For example, in our study, apolipoprotein B decreased by 0.14 SDs from 7 years to 25 years in males compared with a far larger decrease of 0.7 SDs in females during the same time. In the aforementioned MR study,^11^ higher genetically predicted apolipoprotein B was associated with 1.68 higher odds (95% CI 1.54 to 1.85) of CHD in mid-life. In our study, apolipoprotein A1 decreased by 1 SD among males compared with an increase of 0.2 SDs in females from 7 years to 25 years; the same MR study showed that higher genetically predicted apolipoprotein A1 was associated with 0.83 lower odds (95% CI 0.77 to 0.89) of CHD in mid-life. This provides indicative evidence of the potential clinical significance of the large, standardised changes and sex differences observed here in early life, particularly as these standardised differences are likely to persist or widen into mid-life as demonstrated in the previous ALSPAC study including parents of the offspring.^29^ However, further work is required to study the tracking of early adulthood levels of traits and sex differences in traits into mid-life and to formally quantify the potential clinical significance of these early life trajectories in relation to later life atherosclerotic risk using cohorts with long prospective follow-up and measurement of clinical events. In addition, longitudinal mediation studies investigating multiple mediators related to late childhood and adolescence such as adiposity, puberty timing and other health behaviours are required, given that our study identified that higher levels of causal and predictive cardiometabolic traits were not evident among males at 7 years but emerged in males in late childhood and adolescence.

While few studies to date have quantified change in molecular cardiometabolic traits from childhood to early adulthood, our results are comparable with longitudinal analyses of conventional cardiometabolic risk factors. In the Bogalusa Heart Study (n=4321) which included white and black participants aged 5–26 years,^30^ females had higher LDL cholesterol from 5 years to 10 years; however, a male–female cross-over arose for LDL cholesterol during adolescence.^30^ Though LDL cholesterol in that study was measured using the Friedwald equation and thus more prone to measurement error due to inability to exclude intermediate-denisty lipoprotein (IDL) particles from measurement; the general pattern of higher levels in females in early childhood and movement toward a cross-over in the sex difference was observed. In addition, males in that study also had higher HDL cholesterol from age 5 years to 10 years; similar to our study, HDL cholesterol decreased over time in both sexes but to a greater degree in males leading to a male–female cross-over in HDL cholesterol at age ~13 or 14, resulting in higher HDL cholesterol in females after this age, which persisted into early adulthood. The male–female cross-over from higher levels of HDL cholesterol in males in childhood to higher levels in females from adolescence/early adulthood was also observed in the Minneapolis Cohort Study^4^ and Project Heartbeat!^6^ Similar to our study, the Minneapolis Cohort Study demonstrated no strong sex differences in LDL cholesterol at 18 years, while in Project Heartbeat! a sex difference to the disadvantage of females emerged between childhood and age 18 years, which contrasts the narrowing of the sex difference that we observed switching from higher levels of LDL cholesterol at 7 years in females to similar levels between females and males at 25 years. The results in both studies for triglycerides, however, were generally compatible with ours, demonstrating no strong sex differences in early childhood and the emergence of higher levels of triglycerides in males around age 14, which widened and persisted until the end of follow-up at age 18/19 years.

### Strengths and limitations

There are several strengths to our study including the use of 148 molecular cardiometabolic trait concentrations from a targeted metabolomics platform measured on 4 occasions across the early life course. We used multilevel models which take account of clustering of repeated measures within individuals and the correlation between measures over time. Multilevel models also allow inclusion of all participants with at least 1 measure of a risk factor, thereby minimising selection bias compared with complete case approaches. Limitations include the sparsity of measures for the purposes of trajectory modelling, which meant we were not able to explore non-linear patterns of change. While our modelling approach did minimise bias driven by use of complete case approaches for our outcome, bias may still be introduced due to exclusion of participants who did not attend any NMR clinic. Participants included in our analyses were more advantaged than those excluded from analyses. However, our sensitivity analyses weighted by the probability of inclusion in analyses did not differ from our main analyses, suggesting our results are unlikely to be strongly driven by selection into our analyses. Finally, participants were predominantly of white ethnicity and more socially advantaged, and thus, our results may not be generalisable to other populations.

## Conclusion

Childhood and adolescence are important periods for the emergence of sex differences in causal atherogenic lipids and predictive biomarkers for cardiometabolic disease, mostly to the detriment of males. Replication in larger independent studies with more repeated measures is required.

## Data Availability

Data are available upon reasonable request. Data are available upon submission and approval of a research proposal to the ALSPAC Executive. Further information can be found online (https://proposals.epi.bristol.ac.uk/) and by contacting alspac-exec@bristol.ac.uk.
