# Supplementary material for: Sex-specific trajectories of molecular cardiometabolic traits from childhood to young adulthood
Source: Heart. 2023 Mar 13;109(9):674–85. doi: 10.1136/heartjnl-2022-321347 (PMC10176380; doi:10.1136/heartjnl-2022-321347)
Supplement: Supplementary data [file heartjnl-2022-321347supp001.pdf]

SUPPORTING INFORMATION

Sex-specific trajectories of molecular cardiometabolic trait concentrations through childhood, adolescence and young adulthood

Linda M. O'Keeffe [1, 2, 3], Kate Tilling [2, 3], Joshua A. Bell [2, 3], Patrick T. Walsh [1], Matthew A. Lee [2, 3], Deborah A. Lawlor[2,3], George Davey Smith [2, 3,] Patricia M. Kearney [1]

[1] School of Public Health, University College Cork, Ireland
[2] MRC Integrative Epidemiology Unit at the University of Bristol, Bristol, UK
[3] Population Health Sciences, Bristol Medical School, University of Bristol, Bristol, UK

Corresponding author: Dr Linda M. O'Keeffe, School of Public Health, 4th Floor Western Gateway Building, University College Cork, Ireland

Email
linda.okeeffe@bristol.ac.uk;
linda.okeeffe@ucc.ie

O'Keeffe LM, *et al. Heart* 2023; 109:674–685. doi: 10.1136/heartjnl-2022-321347

S1 Appendix Details of quality control

Laboratory

For each sample, the nuclear magnetic resonance (NMR) spectra were analysed for absolute metabolite quantification (molar concentration) in automated fashion. A ridge regression model was applied for quantification of each metabolite to overcome the problems of heavily overlapping spectral data. Quantification of lipoprotein lipid data was performed by calibrating against high performance liquid chromatography methods, and then individually cross-validated against NMR-independent lipid data. Low-molecular-weight metabolites, as well as lipid extract measures, were quantified as mmol/l based on regression modelling calibrated against a set of manually fitted metabolite measures. The calibration data was quantified based on iterative line-shape fitting analysis using PERCH NMR software (PERCH Solutions Ltd., Kuopio, Finland). Absolute quantification could not be directly established for the lipid extract measures due to experimental variation in the lipid extraction protocol. Therefore, serum extract metabolites have been scaled via the total cholesterol as quantified from the native serum LIPO spectrum. We have previously shown strong correlation between the NMR and clinical chemistry measures that are available from both methods.

Data preparation

Prior to statistical analysis, preparation of metabolomics data was performed for each occasion separately using the R package metaboprep (https://github.com/MRCIEU/metaboprep) (version 0.0.1)[1]. Quality control was performed excluding the derived metabolomics measures from missingness and clustering. Briefly, individuals, and then metabolites, with high missingness (>=80%) were removed. Missingness was then re-calculated for individuals and metabolites, with removal based on >=20% missingness. Individuals were then removed based

on total sum abundance, considering outliers as > 5 standard deviations (SDs) away from the mean.

O'Keeffe LM, *et al. Heart* 2023; 109:674–685. doi: 10.1136/heartjnl-2022-321347

S2 Appendix

Model selection

To select the optimal linear spline model for 144 trait concentrations measured from 7y to 25 years (y), we ran a series of models including; model 1: a model with two linear spline periods (7y to 18y and 18y to 25y) model 2: a second model with 2 linear spline periods (7y to 15y and 15y to 25y) and model 3: a single slope model (a single age term which assumed constant change from 7y to 25y). Linear spline periods were chosen to reflect ages in whole years that were closest to mean age at clinics and hence where the density of measures was greatest; note that the same process was carried out to select models for the four traits with measures only available to 18y with a model with two linear spline periods (7y to 15y and 15y to 18y) and single slope (7y to 18y) being compared. For each trait and model, we examined Akaike's Information Criterion (AIC) as an indicator of model fit with lower AIC values indicative of better model fit. Upon selection of the best fitting model based on AIC, we examined, observed and predicted values of models to further assess model fit. Model selection was carried out in both sexes combined to select an optimal model for each trait that would be comparable between the sexes. However, all trajectories were allowed to vary by sex in our final model for each trait by including an interaction term between the linear spline periods/age and sex. S1 Table shows a complete list of all 148 outcomes (144 measured up to 25y and four measured to 18y) and the model details for each outcome). Following the above process of model comparisons, 68 of the 144 outcomes measured to 25y had two linear spline periods from 7-15y and 15-25y, 75 of the 144 outcomes had two linear spline periods from 7-18y and 18-25y and one of the 144 outcomes (acetoacetate) was a single slope model from 7y to 25y. The final models selected for the four outcomes measured only up to 18y had two linear spline periods from 7-15y and 15-18y.

O'Keeffe LM, *et al. Heart* 2023; 109:674–685. doi: 10.1136/heartjnl-2022-321347

Age (in years) was centred at the first available measure (7y). Models were estimated with robust standard errors for both fixed effects and individual level random effects to account for skewed distributions in some traits. Unstructured variance-covariance matrices for the individual level random effects were used to estimate most trajectories; the optimal linear spline model selected and other model details including details of the variance-covariance matrix for each trait are shown in S1 Table. All models included individual level random effects for the intercept and each linear spline period selected. For 35 of 148 outcomes modelled (S1 Table) the covariance of the individual level random effects (level 2) were set to zero for some parameters to improve model convergence. Models allowing occasion level measurement error to vary with age (level 1 random effects for the slopes) were also explored for each risk factor. However, due to difficulties with convergence given sparsity of measures, our models included only a random effect for the intercept at level 1. The model for each outcome took the form of $metabolite_{ij} = \beta_0 + \beta_1 + u_{0j} + (\beta_2 + u_{1j})s_{ij1} + (\beta_3 + u_{2j})s_{ij2} + (\beta_4 + u_{1j})s_{ij1} + (\beta_5 + u_{2j})s_{ij2} + e_{ij}$ where for person j at measurement occasion i; β_0 represents the fixed effect coefficient for the average intercept in males, β_1 represents the difference between the intercept for females compared with males, β_2 and β_3 represent fixed effect coefficients for the average linear slopes of each linear spline in males, β_4 and β_5 represent the difference in the fixed effect coefficients for the average linear slopes of each linear spline in females compared with males,u_{0j} to u_{2j} indicate person-specific (or individual level/level 2) random effects for the intercept and slopes respectively, and e_{ij} represents the occasion-specific residuals or measurement error which was allowed to vary by the intercept.

For each sex, models directly estimate mean predicted level of each metabolite at 7y (the intercept) and mean predicted slopes in original units (mostly mmol/l), with slopes interpreted as change per year in each metabolite in the respective spline period/age period. Following

O'Keeffe LM, *et al. Heart* 2023; 109:674–685. doi: 10.1136/heartjnl-2022-321347

analysis, these estimates were then used to calculate mean predicted absolute change in each trait level from 7y to 25y using the slopes given by each model. The mean predicted level of each trait at 25y was also estimated. Post-analysis, all the above estimates were then converted to SD units by dividing by the sex-combined SD of the observed metabolite at 7y, to aid comparison of results between metabolites.

S1 Table: Metabolic trait subclass, name, units and multilevel model details for each

Molecular class	Lipid, lipoprotein or metabolite name	Units	Knot	Level 2 variance	Level 1 variance
**Extremely large VLDL**	Concentration of chylomicrons and extremely large VLDL particles	mol/l	15	Intercept & splines; unstructured	Intercept
	Total lipids in chylomicrons and extremely large VLDL	mmol/l	15	Intercept & splines; matrix a = (1, 1, 1, 1, 1, 0)	Intercept
	Phospholipids in chylomicrons and extremely large VLDL	mmol/l	15	Intercept & splines; unstructured	Intercept
	Total cholesterol in chylomicrons and extremely large VLDL	mmol/l	18	Intercept & splines; matrix a = (1, 1, 1, 1, 1, 0)	Intercept
	Cholesterol esters in chylomicrons and extremely large VLDL	mmol/l	18	Intercept & splines; matrix a = (1, 1, 1, 1, 1, 0)	Intercept
	Free cholesterol in chylomicrons and extremely large VLDL	mmol/l	15	Intercept & splines; matrix a = (1, 1, 1, 1, 1, 0)	Intercept
	Triglycerides in chylomicrons and extremely large VLDL	mmol/l	15	Intercept & splines; matrix a = (1, 1, 1, 1, 1, 0)	Intercept
**Very large VLDL**	Concentration of very large VLDL particles	mol/l	15	Intercept & splines; unstructured	Intercept
	Total lipids in very large VLDL	mmol/l	15	Intercept & splines; unstructured	Intercept
	Phospholipids in very large VLDL	mmol/l	15	Intercept & splines; unstructured	Intercept
	Total cholesterol in very large VLDL	mmol/l	15	Intercept & splines; matrix a = (1, 1, 1, 1, 1, 0)	Intercept
	Cholesterol esters in very large VLDL	mmol/l	18	Intercept & splines; unstructured	Intercept
	Free cholesterol in very large VLDL	mmol/l	15	Intercept & splines; matrix a = (1, 1, 1, 1, 1, 0)	Intercept
	Triglycerides in very large VLDL	mmol/l	15	Intercept & splines; unstructured	Intercept
**Large VLDL**	Concentration of large VLDL particles	mol/l	15	Intercept & splines; unstructured	Intercept
	Total lipids in large VLDL	mmol/l	15	Intercept & splines; unstructured	Intercept
	Phospholipids in large VLDL	mmol/l	15	Intercept & splines; unstructured	Intercept
	Total cholesterol in large VLDL	mmol/l	15	Intercept & splines; unstructured	Intercept
	Cholesterol esters in large VLDL	mmol/l	15	Intercept & splines; unstructured	Intercept
	Free cholesterol in large VLDL	mmol/l	15	Intercept & splines; unstructured	Intercept
	Triglycerides in large VLDL	mmol/l	15	Intercept & splines; unstructured	Intercept
**Medium VLDL**	Concentration of large VLDL particles	mol/l	15	Intercept & splines; unstructured	Intercept
	Total lipids in small VLDL	mmol/l	15	Intercept & splines; unstructured	Intercept
	Phospholipids in small VLDL	mmol/l	15	Intercept & splines; unstructured	Intercept
	Total cholesterol in small VLDL	mmol/l	15	Intercept & splines; unstructured	Intercept
	Cholesterol esters in small VLDL	mmol/l	15	Intercept & splines; unstructured	Intercept
	Free cholesterol in small VLDL	mmol/l	15	Intercept & splines; unstructured	Intercept
	Triglycerides in small VLDL	mmol/l	15	Intercept & splines; unstructured	Intercept
**Small VLDL**	Concentration of small VLDL particles	mol/l	15	Intercept & splines; unstructured	Intercept
	Total lipids in small VLDL	mmol/l	18	Intercept & splines; unstructured	Intercept
	Phospholipids in small VLDL	mmol/l	15	Intercept & splines; matrix a = (1, 1, 0, 1, 1, 1)	Intercept
	Total cholesterol in small VLDL	mmol/l	18	Intercept & splines; unstructured	Intercept
	Cholesterol esters in small VLDL	mmol/l	18	Intercept & splines; unstructured	Intercept

O'Keeffe LM, *et al. Heart* 2023; 109:674–685. doi: 10.1136/heartjnl-2022-321347

	Free cholesterol in small VLDL	mmol/l	18	Intercept & splines; unstructured	Intercept
	Triglycerides in small VLDL	mmol/l	15	Intercept & splines; unstructured	Intercept
**Very small VLDL**	Concentration of very small VLDL particles	mmol/l	15	Intercept & splines; matrix a = (1, 1, 0, 1, 1, 1)	Intercept
	Total lipids in very small VLDL	mmol/l	18	Intercept & splines; unstructured	Intercept
	Phospholipids in very small VLDL	mmol/l	15	Intercept & splines; matrix a = (1, 1, 0, 1, 1, 1)	Intercept
	Total cholesterol in very small VLDL	mmol/l	18	Intercept & splines; unstructured	Intercept
	Cholesterol esters in very small VLDL	mmol/l	18	Intercept & splines; unstructured	Intercept
	Free cholesterol in very small VLDL	mmol/l	18	Intercept & splines; unstructured	Intercept
	Triglycerides in very small VLDL	mmol/l	18	Intercept & splines; unstructured	Intercept
**IDl**	Concentration of IDL particles	mol/l	18	Intercept & splines; unstructured	Intercept
	Total lipids in IDL	mmol/l	18	Intercept & splines; unstructured	Intercept
	Phospholipids in IDL	mmol/l	18	Intercept & splines; unstructured	Intercept
	Total cholesterol in IDL	mmol/l	18	Intercept & splines; unstructured	Intercept
	Cholesterol esters in IDL	mmol/l	18	Intercept & splines; unstructured	Intercept
	Free cholesterol in IDL	mmol/l	18	Intercept & splines; unstructured	Intercept
	Triglycerides in IDL	mmol/l	18	Intercept & splines; unstructured	Intercept
**Large LDL**	Concentration of large LDL particles	mol/l	18	Intercept & splines; unstructured	Intercept
	Total lipids in large LDL	mmol/l	18	Intercept & splines; unstructured	Intercept
	Phospholipids in large LDL	mmol/l	18	Intercept & splines; unstructured	Intercept
	Total cholesterol in large LDL	mmol/l	18	Intercept & splines; unstructured	Intercept
	Cholesterol esters in large LDL	mmol/l	18	Intercept & splines; unstructured	Intercept
	Free cholesterol in large LDL	mmol/l	18	Intercept & splines; unstructured	Intercept
	Triglycerides in large LDL	mmol/l	18	Intercept & splines; unstructured	Intercept
**Medium LDL**	Concentration of medium LDL particles	mol/l	18	Intercept & splines; unstructured	Intercept
	Total lipids in medium LDL	mmol/l	18	Intercept & splines; unstructured	Intercept
	Phospholipids in medium LDL	mmol/l	15	Intercept & splines; matrix a = (1, 1, 0, 1, 1, 1)	Intercept
	Total cholesterol in medium LDL	mmol/l	18	Intercept & splines; unstructured	Intercept
	Cholesterol esters in medium LDL	mmol/l	18	Intercept & splines; unstructured	Intercept
	Free cholesterol in medium LDL	mmol/l	18	Intercept & splines; unstructured	Intercept
	Triglycerides in medium LDL	mmol/l	18	Intercept & splines; matrix a = (1, 1, 1, 1, 1, 0)	Intercept
**Small LDL**	Concentration of small LDL particles	mol/l	18	Intercept & splines; unstructured	Intercept
	Total lipids in small LDL	mmol/l	18	Intercept & splines; unstructured	Intercept
	Phospholipids in small LDL	mmol/l	15	Intercept & splines; matrix a = (1, 1, 0, 1, 1, 1)	Intercept
	Total cholesterol in small LDL	mmol/l	18	Intercept & splines; unstructured	Intercept
	Cholesterol esters in small LDL	mmol/l	18	Intercept & splines; unstructured	Intercept

	Free cholesterol in small LDL	mmol/l	18	Intercept & splines; unstructured	Intercept
	Triglycerides in small LDL	mmol/l	15	Intercept & splines; matrix a = (1, 1, 1, 1, 1, 0)	Intercept
**Very large HDL**	Concentration of very large HDL particles	mol/l	15	Intercept & splines; unstructured	Intercept
	Total lipids in very large HDL	mmol/l	15	Intercept & splines; unstructured	Intercept
	Phospholipids in very large HDL	mmol/l	15	Intercept & splines; unstructured	Intercept
	Total cholesterol in very large HDL	mmol/l	18	Intercept & splines; unstructured	Intercept
	Cholesterol esters in very large HDL	mmol/l	18	Intercept & splines; unstructured	Intercept
	Free cholesterol in very large HDL	mmol/l	15	Intercept & splines; unstructured	Intercept
	Triglycerides in very large HDL	mmol/l	15	Intercept & splines; unstructured	Intercept
**Large HDL**	Concentration of large HDL particles	mol/l	18	Intercept & splines; unstructured	Intercept
	Total lipids in large HDL	mmol/l	18	Intercept & splines; unstructured	Intercept
	Phospholipids in large HDL	mmol/l	18	Intercept & splines; unstructured	Intercept
	Total cholesterol in large HDL	mmol/l	18	Intercept & splines; unstructured	Intercept
	Cholesterol esters in large HDL	mmol/l	18	Intercept & splines; unstructured	Intercept
	Free cholesterol in large HDL	mmol/l	18	Intercept & splines; unstructured	Intercept
	Triglycerides in large HDL	mmol/l	18	Intercept & splines; unstructured	Intercept
**Medium HDL**	Concentration of medium HDL particles	mol/l	18	Intercept & splines; unstructured	Intercept
	Total lipids in medium HDL	mmol/l	15	Intercept & splines; matrix a = (1, 1, 0, 1, 1, 1)	Intercept
	Phospholipids in medium HDL	mmol/l	15	Intercept & splines; matrix a = (1, 1, 0, 1, 1, 1)	Intercept
	Total cholesterol in medium HDL	mmol/l	18	Intercept & splines; unstructured	Intercept
	Cholesterol esters in medium HDL	mmol/l	18	Intercept & splines; unstructured	Intercept
	Free cholesterol in medium HDL	mmol/l	18	Intercept & splines; unstructured	Intercept
	Triglycerides in medium HDL	mmol/l	15	Intercept & splines; matrix a = (1, 1, 0, 1, 1, 1)	Intercept
**Small HDL**	Concentration of small HDL particles	mol/l	15	Intercept & splines; matrix a = (0, 1, 0, 1, 1, 1)	Intercept
	Total lipids in small HDL	mmol/l	15	Intercept & splines; matrix a = (1, 1, 0, 1, 1, 0)	Intercept
	Phospholipids in small HDL	mmol/l	15	Intercept & splines; matrix a = (1, 1, 0, 1, 1, 1)	Intercept
	Total cholesterol in small HDL	mmol/l	15	Intercept & splines; matrix a = (1, 1, 0, 1, 1, 1)	Intercept
	Cholesterol esters in small HDL	mmol/l	15	Intercept & splines; matrix a = (1, 1, 0, 1, 1, 1)	Intercept
	Free cholesterol in small HDL	mmol/l	18	Intercept & splines; unstructured	Intercept
	Triglycerides in small HDL	mmol/l	15	Intercept & splines; unstructured	Intercept
**Lipoprotein particle size**	Mean diameter for VLDL particles	nm	15	Intercept & splines; unstructured	Intercept
	Mean diameter for LDL particles	nm	15	Intercept & splines; matrix a = (1, 1, 0, 1, 1, 1)	Intercept
	Mean diameter for HDL particles	nm	15	Intercept & splines; unstructured	Intercept
**Cholesterol concentrations**	Total cholesterol	mmol/l	18	Intercept & splines; unstructured	Intercept
	Total cholesterol in VLDL	mmol/l	18	Intercept & splines; unstructured	Intercept

O'Keeffe LM, *et al. Heart* 2023; 109:674–685. doi: 10.1136/heartjnl-2022-321347

	Remnant cholesterol (non-HDL and non-LDL cholesterol)	mmol/l	18	Intercept & splines; unstructured	Intercept
	Total cholesterol in LDL	mmol/l	18	Intercept & splines; unstructured	Intercept
	Total cholesterol in HDL	mmol/l	18	Intercept & splines; unstructured	Intercept
	Total cholesterol in HDL2	mmol/l	18	Intercept & splines; unstructured	Intercept
	Total cholesterol in HDL3	mmol/l	18	Intercept & splines; unstructured	Intercept
	Esterified cholesterol	mmol/l	15	Intercept & splines; matrix a = (1, 1, 0, 1, 1, 1)	Intercept
	Free cholesterol	mmol/l	18	Intercept & splines; unstructured	Intercept
**Glycerides and phospholipid concentrations**	Total triglycerides	mmol/l	15	Intercept & splines; unstructured	Intercept
	Triglycerides in VLDL	mmol/l	15	Intercept & splines; unstructured	Intercept
	Triglycerides in LDL	mmol/l	18	Intercept & splines; matrix a = (1, 1, 1, 1, 1, 0)	Intercept
	Triglycerides in HDL	mmol/l	15	Intercept & splines; unstructured	Intercept
	Diacylglycerol*	mmol/l	15	Intercept & splines; matrix a = (1, 1, 1, 1, 1, 0)	Intercept
	Total phosphoglycerides (mmol/l)	mmol/l	18	Intercept & splines; matrix a = (1, 1, 0, 1, 1, 1)	Intercept
	Phosphatidylcholine and other cholines (mmol/l)	mmol/l	18	Intercept & splines; unstructured	Intercept
	Total cholines (mmol/l)	mmol/l	15	Intercept & splines; unstructured	Intercept
**Apolipoprotein concentrations**	Apolipoprotein A-1	g/l	18	Intercept & splines; unstructured	Intercept
	Apolipoprotein B	g/l	15	Intercept & splines; unstructured	Intercept
**Fatty acid concentrations**	Total fatty acids	mmol/l	15	Intercept & splines; unstructured	Intercept
	Fatty acid length*		15	Intercept & splines; unstructured	Intercept
	Estimated degree of saturation*		15	Intercept & splines; unstructured	Intercept
	22:6, docosahexaenoic acid	mmol/l	15	Intercept & splines; matrix a = (1, 1, 0, 1, 1, 1)	Intercept
	18:2 linoleic acid	mmol/l	15	Intercept & splines; unstructured	Intercept
	Conjugated linoleic acid*	mmol/l	15	Intercept & splines; unstructured	Intercept
	Omega-3 fatty acids	mmol/l	15	Intercept & splines; matrix a = (1, 1, 0, 1, 1, 1)	Intercept
	Omega-6 fatty acids	mmol/l	15	Intercept & splines; unstructured	Intercept
	Polyunsaturated fatty acids	mmol/l	15	Intercept & splines; unstructured	Intercept
	Monounsaturated fatty acids; 16:1, 18:1	mmol/l	18	Intercept & splines; unstructured	Intercept
	Saturated fatty acids	mmol/l	15	Intercept & splines; unstructured	Intercept
**Glycolysis related metabolite**	Glucose	mmol/l	15	Intercept & splines; matrix a = (1, 1, 1, 1, 1, 0)	Intercept
	Lactate	mmol/l	15	Intercept & splines; unstructured	Intercept
	Citrate	mmol/l	18	Intercept & splines; unstructured	Intercept
**Amino acid concentrations**	Alanine	mmol/l	18	Intercept & splines; unstructured	Intercept
	Glutamine	mmol/l	18	Intercept & splines; unstructured	Intercept

O'Keeffe LM, *et al. Heart* 2023; 109:674–685. doi: 10.1136/heartjnl-2022-321347

	Histidine	mmol/l	18	Intercept & splines; matrix a = (1, 1, 1, 1, 1, 0)	Intercept
branched	Isoleucine	mmol/l	18	Intercept & splines; matrix a = (1, 1, 1, 1, 1, 0)	Intercept
branched	Leucine	mmol/l	18	Intercept & splines; unstructured	Intercept
branched	Valine	mmol/l	15	Intercept & splines; unstructured	Intercept
aromatic	Phenylalanine	mmol/l	18	Intercept & splines; unstructured	Intercept
aromatic	Tyrosine	mmol/l	15	Intercept & splines; matrix a = (1, 1, 1, 1, 1, 0)	Intercept
	Acetate	mmol/l	15	Intercept & splines; matrix a = (1, 1, 0, 1, 1, 1)	Intercept
**Ketone body concentrations**	Acetoacetate	mmol/l	Linear	Intercept & slope; unstructured	Intercept
	3-hydroxybutyrate	mmol/l	18	Intercept & splines; matrix a = (0, 1, 1, 1, 1, 1)	Intercept
**Fluid balance marker**	Creatinine	mmol/l	18	Intercept & splines; unstructured	Intercept
	Albumin	mmol/l	18	Intercept & splines; matrix a = (1, 1, 0, 1, 1, 1)	Intercept
**Inflammation marker**	Glycoprotein acetyls, mainly a1-acid glycoprotein	mmol/l	18	Intercept & splines; unstructured	Intercept

*These metabolites were not measured at 25y; all models include data only up to aged 18y. HDL: high-density lipoprotein; IDL: intermediate-density lipoprotein; LDL: low-density lipoprotein; VLDL: very-low-density lipoprotein.

O'Keeffe LM, *et al. Heart* 2023; 109:674–685. doi: 10.1136/heartjnl-2022-321347

S3 Appendix

Characteristics of included vs. included participants

We examined characteristics associated with not being included in our analyses due to missing data on sex or molecular traits. To do this, we compared the socio-demographic characteristics at birth of mothers and partners of participants included in the analyses compared to those excluded from the analyses. All characteristics were measured during pregnancy or at birth through questionnaires or from routine health records.

Marital status was obtained from antenatal questionnaires and classified as never married, widowed, divorced, separated, first marriage, marriage 2 or 3. Household social class was measured as the highest of the mother's or her partner's occupational social class using data on job title and details of occupation collected about the mother and her partner from the mother's questionnaire at 32 weeks gestation. Social class was derived using the standard occupational classification (SOC) codes developed by the United Kingdom Office of Population Census and Surveys and classified as I professional, II managerial and technical, IIINM non-manual, IIIM manual, and IV&V part skilled occupations and unskilled occupations. A questionnaire at 32 weeks gestation asked mothers to report their educational attainment, which was categorized as below O-Level (Ordinary Level; exams taken in different subjects usually at age 15-16 at the completion of legally required school attendance, equivalent to today's UK General Certificate of Secondary Education), O-Level only, A-Level (Advanced-Level; exams taken in different subjects usually at age 18), or university degree or above. A questionnaire at 32 weeks gestation asked partners to report their educational attainment, which was categorized as below O-Level (Ordinary Level; exams taken in different subjects usually at age 15-16 at the completion of legally required school attendance, equivalent to today's UK General Certificate of Secondary Education), O-Level only, A-Level (Advanced-Level; exams taken in different

O'Keeffe LM, *et al. Heart* 2023; 109:674–685. doi: 10.1136/heartjnl-2022-321347

subjects usually at age 18), or university degree or above. Smoking in the first trimester of pregnancy was self-reported by mothers at 18 weeks gestation. Birthweight and gestational age were derived from clinical records. Maternal age was reported in the mother's antenatal questionnaires. Maternal pre-pregnancy weight and height were self-reported in antenatal questionnaires.

Sensitivity analyses

We compared sex differences in metabolic traits at 7y and 25y from the multilevel models with the same differences generated from linear regression analyses at each age separately. This was done to explore the appropriateness of our modelling strategy, compared with more conventional analytic approaches. As outlined, participants included in our analyses required data on sex and at least one measure of each metabolite from 7y to 25y. Mothers of participants included in the analyses tended be of higher household social class and more educated than mothers of participants excluded due to missing data and these differences were similar between females and males (S2 Table). Thus, we performed sensitivity analyses weighted by the probability of being included in our analyses to account for the higher probability of being included due to greater social advantage. The participant level weights were estimated using logistic regression using all socio-demographic characteristics listed above with the addition of sex and were subsequently incorporated into the multilevel models as level two weights which adjust for the unequal probability of selection of the participants. We repeated all SD unit analyses standardising with the sex-specific SD of each metabolite at 7y to examine whether our main results (standardised with sex-combined SDs) were similar.

O'Keeffe LM, *et al. Heart* 2023; 109:674–685. doi: 10.1136/heartjnl-2022-321347

S2 Table: Characteristics of offspring included in analyses compared to those excluded due to missing sex, cardiometabolic trait data or attrition from the cohort						
	**Female participants included n=3,909***	**Female participants excluded**	**N excluded females**	**Male participants included n=3,717***	**Male participants excluded**	**N excluded males**
	**n (%)**	**n (%)**	**n**	**n (%)**	**n (%)**	**n**
**Non-white ethnicity**	75 (2.2)	77 (2.99)	2573	60 (1.8)	112 (3.7)	3050
**Maternal marital status**			2887			3433
Never married	527 (15.1)	680 (23.6)		467 (13.7)	853 (24.9)	
Widowed	<5	6 (0.2)		8 (0.2)	<5	
Divorced	110 (3.2)	143 (5.0)		118 (3.5)	188 (5.5)	
Separated	50 (1.4)	58 (2.0)		36 (1.1)	70 (2.0)	
1st Marriage	2581 (73.9)	1832 (63.5)		2544 (74.8)	2086 (60.8)	
Marriage 2 or 3	224 (6.4)	168 (5.8)		229 (6.7)	234 (6.8)	
**Household social class †**			2337			2758
Professional	517 (15.8)	213 (9.1)		532 (16.7)	275 (10.0)	
Managerial & Technical	1463 (44.6)	893 (38.2)		1404 (44.1)	1062 (38.5)	
Non-Manual	793 (24.2)	637 (27.3)		792 (24.9)	723 (26.2)	
Manual	343 (10.5)	411 (17.6)		328 (10.3)	479 (17.4)	
Part Skilled & Unskilled	161 (4.9)	183 (7.8)		128 (4.0)	219 (7.9)	
**Maternal education**			2605			3088
Less than O level	761 (22.2)	1025 (39.4)		724 (21.6)	1238 (40.1)	
O level	1166 (34.1)	920 (35.3)		1195 (35.7)	1036 (33.6)	
A level	926 (27.1)	435 (16.7)		883 (26.4)	550 (17.8)	
Degree or above	569 (16.6)	225 (8.6)		548 (16.4)	264 (8.6)	
**Mother's Partner's highest educational qualification**			2479			2915
Less than O level	965 (28.9)	1056 (42.6)		863 (26.5)	1258 (43.2)	
O level	717 (21.5)	501 (20.2)		724 (22.2)	609 (20.9)	
A level	916 (27.5)	625 (25.2)		918 (28.2)	657 (22.5)	
Degree or Above	737 (22.1)	297 (12.0)		750 (23.0)	391 (13.4)	
**Maternal smoking during pregnancy**	660 (18.9)	891 (30.5)	2992	657 (19.2)	1126 (32.8)	3438
	Mean (SD)	***Mean (SD)***		***Mean (SD)***	***Mean (SD)***	
**Birthweight (g)**	3370 (512)	3283 (579)	3158	3469 (578)	3395 (631)	3723
**Gestational age (weeks)**	39.6 (1.8)	39.2 (2.9)	3230	39.3 (1.9)	39.0 (3.0)	3812
**Maternal age (years)**	28.8 (4.6)	26.8 (5.0)	3198	29.1 (4.7)	27.1 (5.1)	3778
**Maternal pre-pregnancy BMI (kg/m²)**	22.8 (3.6)	23.1 (4.1)	2448	22.9 (3.8)	23.0 (3.9)	2845

*Represents participants included in models of 144 concentrations with data at all four time points; exact denominators in this table will vary due to missing data for characteristics which were not required for inclusion in analyses.

O'Keeffe LM, *et al. Heart* 2023; 109:674–685. doi: 10.1136/heartjnl-2022-321347

S3 Table: Mean difference in traits at 7y and 25y comparing females with males

	Original units		SD units	
	**Difference (95% CI) in trait at 7y comparing females to males**	**Difference (95% CI) in trait at 25y comparing females to males**	**Difference (95% CI) in trait at 7y comparing females to males**	**Difference (95% CI) in trait at 25y comparing females to males**
Concentration of chylomicrons and extremely large VLDL particles (mol/l)	1.68E-11 (1.11E-11,2.26E-11)	-2.63E-11 (-3.10E-11,-2.16E-11)	0.16(0.11,0.21)	-0.39(-0.45,-0.32)
Total lipids in chylomicrons and extremely large VLDL (mmol/l)	3.51E-03 (2.22E-03,4.79E-03)	-5.65E-03 (-6.65E-03,-4.64E-03)	0.15(0.09,0.2)	-0.39(-0.46,-0.32)
Phospholipids in chylomicrons and extremely large VLDL (mmol/l)	4.25E-04 (2.68E-04,5.81E-04)	-6.51E-04 (-7.77E-04,-5.25E-04)	0.15(0.09,0.2)	-0.36(-0.43,-0.29)
Total cholesterol in chylomicrons and extremely large VLDL (mmol/l)	5.91E-04 (3.97E-04,7.84E-04)	-7.50E-04 (-9.22E-04,-5.77E-04)	0.16(0.11,0.22)	-0.3(-0.37,-0.23)
Cholesterol esters in chylomicrons and extremely large VLDL (mmol/l)	2.97E-04 (2.01E-04,3.94E-04)	-3.59E-04 (-4.52E-04,-2.65E-04)	0.16(0.11,0.22)	-0.26(-0.33,-0.19)
Free cholesterol in chylomicrons and extremely large VLDL (mmol/l)	2.84E-04 (1.79E-04,3.88E-04)	-4.12E-04 (-4.94E-04,-3.31E-04)	0.15(0.09,0.2)	-0.35(-0.42,-0.28)
Triglycerides in chylomicrons and extremely large VLDL (mmol/l)	2.51E-03 (1.57E-03,3.45E-03)	-4.20E-03 (-4.90E-03,-3.49E-03)	0.15(0.09,0.2)	-0.41(-0.48,-0.34)
Concentration of very large VLDL particles (mol/l)	7.96E-11 (4.77E-11,1.11E-10)	-1.66E-10 (-1.94E-10,-1.37E-10)	0.14(0.08,0.19)	-0.4(-0.47,-0.33)
Total lipids in very large VLDL (mmol/l)	7.62E-03 (4.55E-03,1.07E-02)	-1.59E-02 (-1.86E-02,-1.32E-02)	0.14(0.08,0.19)	-0.4(-0.46,-0.33)
Phospholipids in very large VLDL (mmol/l)	1.34E-03 (8.22E-04,1.85E-03)	-2.33E-03 (-2.78E-03,-1.88E-03)	0.14(0.09,0.2)	-0.35(-0.42,-0.28)
Total cholesterol in very large VLDL (mmol/l)	1.79E-03 (1.20E-03,2.38E-03)	-2.82E-03 (-3.33E-03,-2.30E-03)	0.17(0.11,0.22)	-0.38(-0.45,-0.31)
Cholesterol esters in very large VLDL (mmol/l)	9.61E-04 (6.68E-04,1.25E-03)	-1.51E-03 (-1.79E-03,-1.24E-03)	0.18(0.12,0.23)	-0.38(-0.44,-0.31)
Free cholesterol in very large VLDL (mmol/l)	8.59E-04 (5.66E-04,1.15E-03)	-1.21E-03 (-1.45E-03,-9.74E-04)	0.16(0.11,0.21)	-0.35(-0.42,-0.28)
Triglycerides in very large VLDL (mmol/l)	4.49E-03 (2.52E-03,6.46E-03)	-1.07E-02 (-1.25E-02,-8.97E-03)	0.12(0.07,0.18)	-0.41(-0.48,-0.34)
Concentration of large VLDL particles (mol/l)	3.61E-10 (2.16E-10,5.06E-10)	-1.11E-09 (-1.28E-09,-9.44E-10)	0.14(0.08,0.19)	-0.43(-0.5,-0.37)
Total lipids in large VLDL (mmol/l)	2.25E-02 (1.38E-02,3.12E-02)	-6.39E-02 (-7.37E-02,-5.41E-02)	0.14(0.09,0.2)	-0.43(-0.5,-0.37)
Phospholipids in large VLDL (mmol/l)	4.22E-03 (2.66E-03,5.78E-03)	-1.08E-02 (-1.26E-02,-9.04E-03)	0.15(0.09,0.2)	-0.4(-0.47,-0.34)
Total cholesterol in large VLDL (mmol/l)	5.87E-03 (3.91E-03,7.83E-03)	-1.32E-02 (-1.54E-02,-1.10E-02)	0.16(0.11,0.22)	-0.4(-0.47,-0.34)
Cholesterol esters in large VLDL (mmol/l)	3.05E-03 (2.14E-03,3.96E-03)	-6.89E-03 (-7.97E-03,-5.82E-03)	0.18(0.13,0.24)	-0.42(-0.49,-0.36)
Free cholesterol in large VLDL (mmol/l)	2.83E-03 (1.76E-03,3.89E-03)	-6.30E-03 (-7.41E-03,-5.19E-03)	0.15(0.09,0.2)	-0.38(-0.45,-0.31)
Triglycerides in large VLDL (mmol/l)	1.24E-02 (7.18E-03,1.76E-02)	-3.99E-02 (-4.58E-02,-3.41E-02)	0.13(0.08,0.18)	-0.45(-0.52,-0.38)
Concentration of medium VLDL particles (mol/l)	1.05E-09 (7.37E-10,1.37E-09)	-3.34E-09 (-3.78E-09,-2.90E-09)	0.18(0.13,0.23)	-0.49(-0.56,-0.43)
Total lipids in medium VLDL (mmol/l)	3.73E-02 (2.66E-02,4.80E-02)	-1.09E-01 (-1.23E-01,-9.44E-02)	0.19(0.14,0.24)	-0.49(-0.55,-0.42)
Phospholipids in medium VLDL (mmol/l)	7.60E-03 (5.56E-03,9.65E-03)	-1.97E-02 (-2.25E-02,-1.70E-02)	0.2(0.15,0.26)	-0.46(-0.52,-0.39)
Total cholesterol in medium VLDL (mmol/l)	1.19E-02 (9.16E-03,1.46E-02)	-2.07E-02 (-2.44E-02,-1.70E-02)	0.23(0.18,0.29)	-0.36(-0.43,-0.3)
Cholesterol esters in medium VLDL (mmol/l)	7.01E-03 (5.52E-03,8.49E-03)	-8.63E-03 (-1.06E-02,-6.66E-03)	0.25(0.2,0.31)	-0.28(-0.35,-0.22)
Free cholesterol in medium VLDL (mmol/l)	4.91E-03 (3.54E-03,6.27E-03)	-1.20E-02 (-1.38E-02,-1.02E-02)	0.2(0.14,0.25)	-0.43(-0.5,-0.37)
Triglycerides in medium VLDL (mmol/l)	1.78E-02 (1.18E-02,2.39E-02)	-6.84E-02 (-7.65E-02,-6.04E-02)	0.16(0.11,0.21)	-0.55(-0.61,-0.48)
Concentration of small VLDL particles (mol/l)	1.46E-09 (1.15E-09,1.77E-09)	-3.08E-09 (-3.55E-09,-2.62E-09)	0.25(0.2,0.31)	-0.42(-0.48,-0.35)
Total lipids in small VLDL (mmol/l)	3.40E-02 (2.79E-02,4.01E-02)	-5.84E-02 (-6.74E-02,-4.94E-02)	0.29(0.24,0.35)	-0.41(-0.47,-0.35)
Phospholipids in small VLDL (mmol/l)	6.19E-03 (4.97E-03,7.42E-03)	-8.95E-03 (-1.08E-02,-7.14E-03)	0.27(0.22,0.32)	-0.31(-0.37,-0.25)
Total cholesterol in small VLDL (mmol/l)	1.58E-02 (1.36E-02,1.80E-02)	-1.56E-02 (-1.88E-02,-1.23E-02)	0.37(0.32,0.42)	-0.3(-0.36,-0.23)
Cholesterol esters in small VLDL (mmol/l)	1.12E-02 (9.71E-03,1.27E-02)	-9.38E-03 (-1.15E-02,-7.23E-03)	0.39(0.34,0.44)	-0.27(-0.33,-0.21)

O'Keeffe LM, *et al. Heart* 2023; 109:674–685. doi: 10.1136/heartjnl-2022-321347

S3 Table: Mean difference in traits at 7y and 25y comparing females with males

	Original units		SD units	
	**Difference (95% CI) in trait at 7y comparing females to males**	**Difference (95% CI) in trait at 25y comparing females to males**	**Difference (95% CI) in trait at 7y comparing females to males**	**Difference (95% CI) in trait at 25y comparing females to males**
Free cholesterol in small VLDL (mmol/l)	4.61E-03 (3.80E-03,5.43E-03)	-6.15E-03 (-7.37E-03,-4.93E-03)	0.3(0.24,0.35)	-0.32(-0.38,-0.25)
Triglycerides in small VLDL (mmol/l)	1.21E-02 (8.97E-03,1.53E-02)	-3.32E-02 (-3.75E-02,-2.89E-02)	0.21(0.15,0.26)	-0.49(-0.56,-0.43)
Concentration of very small VLDL particles (mol/l)	1.79E-09 (1.55E-09,2.03E-09)	5.40E-10 (1.04E-10,9.77E-10)	0.39(0.34,0.44)	0.08(0.01,0.14)
Total lipids in very small VLDL (mmol/l)	2.84E-02 (2.47E-02,3.21E-02)	2.98E-03 (-2.65E-03,8.60E-03)	0.39(0.34,0.44)	0.03(-0.03,0.09)
Phospholipids in very small VLDL (mmol/l)	8.72E-03 (7.46E-03,9.98E-03)	5.91E-03 (4.08E-03,7.74E-03)	0.36(0.31,0.41)	0.2(0.14,0.26)
Total cholesterol in very small VLDL (mmol/l)	1.43E-02 (1.20E-02,1.66E-02)	7.17E-04 (-2.18E-03,3.61E-03)	0.32(0.27,0.37)	0.02(-0.05,0.08)
Cholesterol esters in very small VLDL (mmol/l)	1.06E-02 (9.07E-03,1.22E-02)	7.42E-04 (-1.26E-03,2.74E-03)	0.35(0.3,0.4)	0.02(-0.04,0.09)
Free cholesterol in very small VLDL (mmol/l)	3.65E-03 (2.84E-03,4.47E-03)	4.21E-05 (-9.04E-04,9.88E-04)	0.23(0.18,0.28)	0.02(-0.06,0.07)
Triglycerides in very small VLDL (mmol/l)	5.41E-03 (4.39E-03,6.43E-03)	-2.49E-03 (-3.89E-03,-1.10E-03)	0.28(0.22,0.33)	-0.11(-0.18,-0.05)
Concentration of IDL particles (mol/l)	4.19E-09 (3.47E-09,4.90E-09)	4.78E-09 (3.52E-09,6.03E-09)	0.3(0.25,0.36)	0.23(0.17,0.29)
Total lipids in IDL (mmol/l)	5.60E-02 (4.69E-02,6.51E-02)	4.73E-02 (3.44E-02,6.02E-02)	0.32(0.27,0.37)	0.22(0.16,0.28)
Phospholipids in IDL (mmol/l)	1.28E-02 (1.04E-02,1.53E-02)	1.41E-02 (1.09E-02,1.74E-02)	0.27(0.22,0.32)	0.26(0.2,0.32)
Total cholesterol in IDL (mmol/l)	3.79E-02 (3.18E-02,4.41E-02)	2.44E-02 (1.55E-02,3.32E-02)	0.32(0.27,0.37)	0.17(0.11,0.23)
Cholesterol esters in IDL (mmol/l)	2.82E-02 (2.38E-02,3.25E-02)	1.27E-02 (6.27E-03,1.91E-02)	0.33(0.28,0.38)	0.12(0.06,0.18)
Free cholesterol in IDL (mmol/l)	9.70E-03 (7.77E-03,1.16E-02)	1.17E-02 (9.21E-03,1.42E-02)	0.26(0.21,0.31)	0.28(0.22,0.34)
Triglycerides in IDL (mmol/l)	5.19E-03 (4.10E-03,6.28E-03)	8.87E-03 (7.62E-03,1.01E-02)	0.25(0.2,0.3)	0.43(0.37,0.49)
Concentration of large LDL particles (mol/l)	6.66E-09 (5.32E-09,8.00E-09)	6.20E-09 (3.89E-09,8.50E-09)	0.26(0.21,0.31)	0.17(0.1,0.23)
Total lipids in large LDL (mmol/l)	6.39E-02 (5.23E-02,7.55E-02)	4.14E-02 (2.50E-02,5.78E-02)	0.28(0.23,0.34)	0.15(0.09,0.21)
Phospholipids in large LDL (mmol/l)	1.32E-02 (1.08E-02,1.55E-02)	9.27E-03 (5.85E-03,1.27E-02)	0.29(0.24,0.34)	0.16(0.1,0.23)
Total cholesterol in large LDL (mmol/l)	4.66E-02 (3.81E-02,5.50E-02)	1.95E-02 (7.36E-03,3.17E-02)	0.28(0.23,0.34)	0.1(0.04,0.16)
Cholesterol esters in large LDL (mmol/l)	3.58E-02 (2.95E-02,4.22E-02)	9.62E-03 (2.99E-04,1.89E-02)	0.29(0.24,0.34)	0.06(0.01,0.12)
Free cholesterol in large LDL (mmol/l)	1.07E-02 (8.59E-03,1.29E-02)	9.91E-03 (7.02E-03,1.28E-02)	0.26(0.21,0.31)	0.21(0.15,0.27)
Triglycerides in large LDL (mmol/l)	4.09E-03 (2.88E-03,5.31E-03)	1.26E-02 (1.14E-02,1.37E-02)	0.18(0.12,0.23)	0.63(0.57,0.69)
Concentration of medium LDL particles (mol/l)	5.57E-09 (4.39E-09,6.76E-09)	2.46E-09 (3.41E-10,4.59E-09)	0.24(0.19,0.3)	0.07(0.01,0.13)
Total lipids in medium LDL (mmol/l)	3.81E-02 (3.09E-02,4.54E-02)	9.72E-03 (-1.04E-03,2.05E-02)	0.27(0.22,0.33)	0.06(-0.01,0.12)
Phospholipids in medium LDL (mmol/l)	8.01E-03 (6.64E-03,9.38E-03)	4.33E-03 (2.19E-03,6.47E-03)	0.3(0.25,0.36)	0.12(0.06,0.18)
Total cholesterol in medium LDL (mmol/l)	2.83E-02 (2.29E-02,3.37E-02)	-5.03E-04 (-8.72E-03,7.72E-03)	0.27(0.22,0.32)	-0.01(-0.07,0.06)
Cholesterol esters in medium LDL (mmol/l)	2.31E-02 (1.86E-02,2.75E-02)	-1.68E-03 (-8.26E-03,4.91E-03)	0.27(0.22,0.32)	-0.02(-0.08,0.05)
Free cholesterol in medium LDL (mmol/l)	5.22E-03 (4.22E-03,6.22E-03)	1.17E-03 (-5.04E-04,2.85E-03)	0.27(0.22,0.32)	0.04(-0.02,0.1)
Triglycerides in medium LDL (mmol/l)	1.77E-03 (9.68E-04,2.57E-03)	7.42E-03 (6.80E-03,8.05E-03)	0.11(0.06,0.17)	0.7(0.64,0.75)
Concentration of small LDL particles (mol/l)	5.42E-09 (4.13E-09,6.72E-09)	2.37E-09 (-1.85E-10,4.93E-09)	0.22(0.17,0.27)	0.06(0.002,0.12)
Total lipids in small LDL (mmol/l)	2.27E-02 (1.81E-02,2.73E-02)	5.67E-03 (-1.54E-03,1.29E-02)	0.26(0.2,0.31)	0.05(-0.01,0.11)
Phospholipids in small LDL (mmol/l)	4.50E-03 (3.54E-03,5.46E-03)	4.06E-03 (2.39E-03,5.72E-03)	0.25(0.19,0.3)	0.15(0.09,0.21)

S3 Table: Mean difference in traits at 7y and 25y comparing females with males

	Original units		SD units	
	**Difference (95% CI) in trait at 7y comparing females to males**	**Difference (95% CI) in trait at 25y comparing females to males**	**Difference (95% CI) in trait at 7y comparing females to males**	**Difference (95% CI) in trait at 25y comparing females to males**
Total cholesterol in small LDL (mmol/l)	1.67E-02 (1.34E-02,2.01E-02)	2.15E-04 (-5.06E-03,5.49E-03)	0.26(0.2,0.31)	0.02(-0.06,0.06)
Cholesterol esters in small LDL (mmol/l)	1.40E-02 (1.11E-02,1.68E-02)	-9.53E-06 (-4.15E-03,4.13E-03)	0.25(0.2,0.3)	0.02(-0.06,0.06)
Free cholesterol in small LDL (mmol/l)	2.77E-03 (2.15E-03,3.39E-03)	2.32E-04 (-9.59E-04,1.42E-03)	0.23(0.18,0.28)	0.01(-0.05,0.07)
Triglycerides in small LDL (mmol/l)	1.59E-03 (1.10E-03,2.09E-03)	2.97E-03 (2.52E-03,3.42E-03)	0.17(0.12,0.22)	0.42(0.35,0.48)
Concentration of very large HDL particles (mol/l)	-1.16E-08 (-1.77E-08,-5.51E-09)	1.53E-07 (1.44E-07,1.63E-07)	-0.1(-0.15,-0.05)	0.82(0.77,0.87)
Total lipids in very large HDL (mmol/l)	-1.58E-02 (-2.31E-02,-8.47E-03)	1.51E-01 (1.41E-01,1.61E-01)	-0.11(-0.17,-0.06)	0.8(0.75,0.85)
Phospholipids in very large HDL (mmol/l)	-8.35E-03 (-1.23E-02,-4.43E-03)	9.26E-02 (8.71E-02,9.81E-02)	-0.11(-0.16,-0.06)	0.87(0.81,0.92)
Total cholesterol in very large HDL (mmol/l)	-8.26E-03 (-1.17E-02,-4.78E-03)	5.78E-02 (5.33E-02,6.24E-02)	-0.13(-0.18,-0.07)	0.72(0.67,0.78)
Cholesterol esters in very large HDL (mmol/l)	-6.22E-03 (-8.75E-03,-3.68E-03)	3.80E-02 (3.47E-02,4.13E-02)	-0.13(-0.18,-0.08)	0.68(0.62,0.74)
Free cholesterol in very large HDL (mmol/l)	-2.03E-03 (-3.03E-03,-1.03E-03)	1.98E-02 (1.85E-02,2.11E-02)	-0.11(-0.16,-0.05)	0.8(0.75,0.85)
Triglycerides in very large HDL (mmol/l)	5.59E-04 (2.76E-04,8.41E-04)	2.46E-03 (2.11E-03,2.81E-03)	0.11(0.05,0.16)	0.42(0.36,0.48)
Concentration of large HDL particles (mol/l)	-4.38E-08 (-5.58E-08,-3.18E-08)	4.22E-07 (3.96E-07,4.48E-07)	-0.19(-0.24,-0.14)	0.86(0.8,0.91)
Total lipids in large HDL (mmol/l)	-3.19E-02 (-4.10E-02,-2.28E-02)	2.69E-01 (2.53E-01,2.86E-01)	-0.18(-0.24,-0.13)	0.85(0.8,0.91)
Phospholipids in large HDL (mmol/l)	-1.33E-02 (-1.73E-02,-9.29E-03)	1.22E-01 (1.14E-01,1.29E-01)	-0.17(-0.23,-0.12)	0.86(0.81,0.92)
Total cholesterol in large HDL (mmol/l)	-1.88E-02 (-2.39E-02,-1.38E-02)	1.39E-01 (1.30E-01,1.48E-01)	-0.2(-0.25,-0.14)	0.83(0.78,0.89)
Cholesterol esters in large HDL (mmol/l)	-1.53E-02 (-1.92E-02,-1.13E-02)	1.06E-01 (9.90E-02,1.12E-01)	-0.2(-0.25,-0.15)	0.83(0.78,0.89)
Free cholesterol in large HDL (mmol/l)	-3.58E-03 (-4.67E-03,-2.50E-03)	3.31E-02 (3.10E-02,3.52E-02)	-0.17(-0.22,-0.12)	0.83(0.78,0.89)
Triglycerides in large HDL (mmol/l)	2.59E-04 (7.13E-05,4.47E-04)	8.79E-03 (8.16E-03,9.42E-03)	0.07(0.02,0.13)	0.76(0.71,0.81)
Concentration of medium HDL particles (mol/l)	-2.98E-08 (-3.90E-08,-2.06E-08)	2.75E-07 (2.48E-07,3.01E-07)	-0.18(-0.23,-0.12)	0.62(0.56,0.68)
Total lipids in medium HDL (mmol/l)	-1.49E-02 (-1.96E-02,-1.02E-02)	1.25E-01 (1.14E-01,1.36E-01)	-0.17(-0.23,-0.12)	0.64(0.59,0.7)
Phospholipids in medium HDL (mmol/l)	-8.09E-03 (-1.07E-02,-5.51E-03)	6.09E-02 (5.60E-02,6.58E-02)	-0.17(-0.23,-0.12)	0.7(0.64,0.75)
Total cholesterol in medium HDL (mmol/l)	-8.22E-03 (-1.04E-02,-6.02E-03)	5.73E-02 (5.10E-02,6.36E-02)	-0.2(-0.26,-0.15)	0.54(0.48,0.6)
Cholesterol esters in medium HDL (mmol/l)	-7.33E-03 (-9.14E-03,-5.52E-03)	4.33E-02 (3.84E-02,4.83E-02)	-0.22(-0.27,-0.16)	0.52(0.46,0.58)
Free cholesterol in medium HDL (mmol/l)	-9.05E-04 (-1.31E-03,-4.99E-04)	1.39E-02 (1.26E-02,1.53E-02)	-0.12(-0.18,-0.07)	0.6(0.54,0.66)
Triglycerides in medium HDL (mmol/l)	1.26E-03 (8.34E-04,1.70E-03)	2.73E-03 (2.03E-03,3.43E-03)	0.16(0.11,0.21)	0.24(0.18,0.31)
Concentration of small HDL particles (mol/l)	-2.83E-08 (-4.23E-08,-1.43E-08)	1.87E-07 (1.46E-07,2.29E-07)	-0.11(-0.17,-0.06)	0.26(0.2,0.32)
Total lipids in small HDL (mmol/l)	-9.61E-03 (-1.36E-02,-5.65E-03)	4.13E-02 (3.16E-02,5.10E-02)	-0.14(-0.19,-0.08)	0.25(0.19,0.31)
Phospholipids in small HDL (mmol/l)	-7.62E-03 (-1.01E-02,-5.15E-03)	2.02E-02 (1.57E-02,2.46E-02)	-0.17(-0.22,-0.11)	0.27(0.21,0.33)
Total cholesterol in small HDL (mmol/l)	-3.28E-03 (-5.76E-03,-8.05E-04)	2.45E-02 (1.84E-02,3.05E-02)	-0.07(-0.13,-0.02)	0.24(0.18,0.3)
Cholesterol esters in small HDL (mmol/l)	-8.07E-04 (-3.02E-03,1.41E-03)	2.08E-02 (1.54E-02,2.61E-02)	-0.02(-0.08,0.04)	0.23(0.17,0.29)
Free cholesterol in small HDL (mmol/l)	-2.55E-03 (-3.07E-03,-2.02E-03)	3.45E-03 (2.50E-03,4.41E-03)	-0.27(-0.32,-0.21)	0.22(0.16,0.28)
Triglycerides in small HDL (mmol/l)	1.34E-03 (8.78E-04,1.81E-03)	-1.30E-03 (-1.90E-03,-6.97E-04)	0.16(0.1,0.21)	-0.14(-0.2,-0.07)
Mean diameter for VLDL particles (mm)	1.28E-01 (5.69E-02,2.00E-01)	-7.44E-01 (-8.16E-01,-6.72E-01)	0.1(0.04,0.15)	-0.64(-0.71,-0.58)

O'Keeffe LM, *et al. Heart* 2023; 109:674–685. doi: 10.1136/heartjnl-2022-321347

S3 Table: Mean difference in traits at 7y and 25y comparing females with males

	Original units		SD units	
	**Difference (95% CI) in trait at 7y comparing females to males**	**Difference (95% CI) in trait at 25y comparing females to males**	**Difference (95% CI) in trait at 7y comparing females to males**	**Difference (95% CI) in trait at 25y comparing females to males**
Mean diameter for LDL particles (mm)	9.55E-03 (3.94E-03,1.52E-02)	4.54E-02 (3.45E-02,5.63E-02)	0.09(0.04,0.15)	0.25(0.19,0.31)
Mean diameter for HDL particles (mm)	-1.77E-02 (-2.57E-02,-9.76E-03)	2.05E-01 (1.92E-01,2.18E-01)	-0.12(-0.17,-0.06)	0.84(0.78,0.89)
Serum total cholesterol (mmol/l)	1.41E-01 (1.10E-01,1.72E-01)	2.57E-01 (2.08E-01,3.05E-01)	0.24(0.18,0.29)	0.32(0.26,0.38)
Total cholesterol in VLDL (mmol/l)	5.08E-02 (4.27E-02,5.88E-02)	-5.20E-02 (-6.35E-02,-4.04E-02)	0.33(0.28,0.38)	-0.29(-0.35,-0.22)
Remnant cholesterol (non-HDL, non-LDL -cholesterol) (mmol/l)	8.86E-02 (7.64E-02,1.01E-01)	-2.78E-02 (-4.65E-02,-9.20E-03)	0.37(0.32,0.42)	-0.09(-0.15,-0.03)
Total cholesterol in LDL (mmol/l)	9.17E-02 (7.45E-02,1.09E-01)	1.92E-02 (-6.21E-03,4.47E-02)	0.28(0.22,0.33)	0.05(-0.01,0.11)
Total cholesterol in HDL (mmol/l)	-3.90E-02 (-5.00E-02,-2.81E-02)	2.67E-01 (2.48E-01,2.85E-01)	-0.19(-0.24,-0.14)	0.78(0.73,0.84)
Total cholesterol in HDL2 (mmol/l)	-2.97E-02 (-3.70E-02,-2.25E-02)	2.48E-01 (2.31E-01,2.65E-01)	-0.21(-0.27,-0.16)	0.8(0.75,0.85)
Total cholesterol in HDL3 (mmol/l)	-9.15E-03 (-1.31E-02,-5.19E-03)	2.17E-02 (1.93E-02,2.41E-02)	-0.12(-0.18,-0.07)	0.61(0.55,0.68)
Esterified cholesterol (mmol/l)	9.74E-02 (7.49E-02,1.20E-01)	1.88E-01 (1.54E-01,2.22E-01)	0.23(0.18,0.28)	0.33(0.27,0.39)
Free cholesterol (mmol/l)	4.48E-02 (3.54E-02,5.43E-02)	8.63E-02 (7.24E-02,1.00E-01)	0.25(0.19,0.3)	0.37(0.31,0.43)
Serum total triglycerides (mmol/l)	7.20E-02 (5.14E-02,9.26E-02)	-1.11E-01 (-1.36E-01,-8.56E-02)	0.19(0.14,0.24)	-0.29(-0.36,-0.22)
Triglycerides in VLDL (mmol/l)	5.49E-02 (3.70E-02,7.29E-02)	-1.59E-01 (-1.81E-01,-1.37E-01)	0.17(0.11,0.22)	-0.47(-0.54,-0.41)
Triglycerides in LDL (mmol/l)	7.31E-03 (4.85E-03,9.78E-03)	2.27E-02 (2.05E-02,2.49E-02)	0.16(0.1,0.21)	0.61(0.55,0.67)
Triglycerides in HDL (mmol/l)	3.49E-03 (2.36E-03,4.62E-03)	1.26E-02 (1.09E-02,1.44E-02)	0.17(0.11,0.22)	0.45(0.38,0.51)
Diacylglycerol (mmol/l)*	1.00E-03 (3.85E-04,1.62E-03)	9.52E-04 (2.71E-04,1.63E-03)	0.09(0.04,0.15)	0.1(0.03,0.18)
Total phosphoglycerides (mmol/l)	2.27E-02 (7.82E-03,3.75E-02)	2.15E-01 (1.97E-01,2.34E-01)	0.08(0.03,0.14)	0.65(0.6,0.71)
Phosphatidylcholine and other cholines (mmol/l)	1.31E-02 (-1.70E-03,2.78E-02)	1.65E-01 (1.44E-01,1.86E-01)	0.05(-0.01,0.1)	0.47(0.41,0.53)
Total cholines (mmol/l)	2.95E-02 (1.34E-02,4.56E-02)	2.40E-01 (2.17E-01,2.62E-01)	0.1(0.05,0.15)	0.61(0.56,0.67)
Apolipoprotein A-I (g/l)	-8.28E-03 (-1.42E-02,-2.36E-03)	1.40E-01 (1.28E-01,1.51E-01)	-0.07(-0.13,-0.02)	0.68(0.62,0.73)
Apolipoprotein B (g/l)	4.42E-02 (3.77E-02,5.06E-02)	-2.25E-02 (-3.21E-02,-1.29E-02)	0.36(0.31,0.41)	-0.15(-0.21,-0.08)
Total fatty acids (mmol/l)	3.72E-01 (2.86E-01,4.58E-01)	4.61E-01 (3.41E-01,5.81E-01)	0.23(0.18,0.29)	0.24(0.18,0.3)
Fatty acid length*	4.51E-03 (-1.56E-02,2.46E-02)	7.86E-03 (-1.94E-02,3.51E-02)	0.01(-0.04,0.07)	0.02(-0.05,0.1)
Estimated degree of unsaturation*	-3.97E-03 (-7.38E-03,-5.72E-04)	7.57E-03 (2.75E-03,1.24E-02)	-0.07(-0.12,-0.01)	0.12(0.04,0.19)
22:6, docosahexaenoic acid (mmol/l)	5.08E-03 (3.64E-03,6.53E-03)	1.91E-02 (1.72E-02,2.11E-02)	0.19(0.14,0.24)	0.59(0.53,0.65)
18:2, linoleic acid (mmol/l)	1.02E-01 (7.83E-02,1.26E-01)	1.25E-01 (9.22E-02,1.58E-01)	0.23(0.18,0.28)	0.23(0.17,0.29)
Conjugated linoleic acid (mmol/l)*	2.09E-03 (1.08E-03,3.09E-03)	1.42E-03 (3.96E-04,2.45E-03)	0.12(0.06,0.17)	0.1(0.03,0.18)
Omega-3 fatty acids (mmol/l)	1.13E-02 (7.59E-03,1.50E-02)	7.34E-03 (2.41E-03,1.23E-02)	0.17(0.11,0.22)	0.09(0.03,0.16)
Omega-6 fatty acids (mmol/l)	1.09E-01 (8.28E-02,1.35E-01)	1.98E-01 (1.61E-01,2.35E-01)	0.22(0.17,0.27)	0.32(0.26,0.38)
Polyunsaturated fatty acids (mmol/l)	1.20E-01 (9.18E-02,1.49E-01)	2.06E-01 (1.65E-01,2.46E-01)	0.22(0.17,0.28)	0.3(0.24,0.37)
Monounsaturated fatty acids; 16:1, 18:1 (mmol/l)	1.34E-01 (1.04E-01,1.64E-01)	8.93E-02 (4.82E-02,1.30E-01)	0.24(0.19,0.29)	0.14(0.07,0.2)
Saturated fatty acids (mmol/l)	1.14E-01 (7.69E-02,1.50E-01)	1.59E-01 (1.15E-01,2.04E-01)	0.17(0.11,0.22)	0.22(0.16,0.29)
Glucose (mmol/l)	-7.74E-02 (-1.06E-01,-4.84E-02)	-1.92E-01 (-2.25E-01,-1.59E-01)	-0.15(-0.21,-0.09)	-0.42(-0.49,-0.35)

O'Keeffe LM, *et al. Heart* 2023; 109:674–685. doi: 10.1136/heartjnl-2022-321347

S3 Table: Mean difference in traits at 7y and 25y comparing females with males

	Original units		SD units	
	**Difference (95% CI) in trait at 7y comparing females to males**	**Difference (95% CI) in trait at 25y comparing females to males**	**Difference (95% CI) in trait at 7y comparing females to males**	**Difference (95% CI) in trait at 25y comparing females to males**
Lactate (mmol/l)	5.99E-02 (3.24E-02,8.73E-02)	-7.08E-03 (-3.62E-02,2.20E-02)	0.12(0.07,0.18)	-0.02(-0.08,0.05)
Citrate (mmol/l)	3.05E-03 (1.67E-03,4.42E-03)	-1.88E-03 (-3.51E-03,-2.46E-04)	0.12(0.07,0.18)	-0.08(-0.14,-0.01)
Alanine (mmol/l)	4.33E-03 (7.96E-04,7.87E-03)	-9.16E-03 (-1.29E-02,-5.47E-03)	0.07(0.01,0.12)	-0.16(-0.23,-0.1)
Glutamine (mmol/l)	2.54E-02 (2.23E-02,2.86E-02)	-6.36E-02 (-6.83E-02,-5.89E-02)	0.44(0.39,0.49)	-0.83(-0.89,-0.77)
Histidine (mmol/l)	2.38E-03 (1.71E-03,3.05E-03)	-3.20E-03 (-3.75E-03,-2.66E-03)	0.17(0.12,0.21)	-0.4(-0.47,-0.33)
Isoleucine (mmol/l)	1.81E-03 (8.22E-04,2.81E-03)	-9.90E-03 (-1.07E-02,-9.15E-03)	0.1(0.05,0.16)	-0.83(-0.9,-0.77)
Leucine (mmol/l)	-2.03E-04 (-1.03E-03,6.23E-04)	-1.22E-02 (-1.30E-02,-1.14E-02)	-0.01(-0.07,0.04)	-0.92(-0.98,-0.86)
Valine (mmol/l)	2.09E-03 (1.20E-04,4.06E-03)	-2.49E-02 (-2.67E-02,-2.32E-02)	0.06(-0.004,0.12)	-0.85(-0.91,-0.79)
Phenylalanine (mmol/l)	-2.86E-04 (-7.56E-04,1.84E-04)	-2.00E-03 (-2.42E-03,-1.58E-03)	-0.03(-0.09,0.02)	-0.32(-0.38,-0.25)
Tyrosine (mmol/l)	1.67E-04 (-7.27E-04,1.06E-03)	-5.41E-03 (-6.02E-03,-4.80E-03)	0.01(-0.05,0.07)	-0.55(-0.62,-0.49)
Acetate (mmol/l)	9.42E-04 (-4.36E-04,2.32E-03)	-5.65E-03 (-7.92E-03,-3.38E-03)	0.04(-0.02,0.1)	-0.16(-0.22,-0.1)
Acetoacetate (mmol/l)	1.82E-03 (-3.99E-04,4.05E-03)	-2.05E-03 (-3.25E-03,-8.54E-04)	0.04(-0.01,0.09)	-0.2(-0.31,-0.08)
3-hydroxybutyrate (mmol/l)	1.21E-02 (6.54E-03,1.76E-02)	1.61E-02 (1.09E-02,2.14E-02)	0.12(0.07,0.18)	0.18(0.12,0.25)
Creatinine (mmol/l)	6.22E-04 (3.06E-04,9.38E-04)	-1.17E-02 (-1.22E-02,-1.12E-02)	0.1(0.05,0.15)	-1.25(-1.3,-1.2)
Albumin (mmol/l)	1.08E-03 (8.79E-04,1.27E-03)	-2.89E-03 (-3.24E-03,-2.54E-03)	0.3(0.24,0.35)	-0.52(-0.58,-0.45)
Glycoprotein acetyls, mainly a1-acid glycoprotein (mmol/l)	3.23E-02 (2.48E-02,3.98E-02)	1.75E-02 (6.60E-03,2.85E-02)	0.23(0.18,0.29)	0.1(0.04,0.17)

*These metabolites (diacylglycerol, fatty acid chain length, estimated degree of saturation and conjugated linoleic acid) were not measured at 25y; all models include data only up to aged 18y and values in this table for these traits are at 7y and 18y respectively. HDL: high-density lipoprotein; IDL: intermediate-density lipoprotein; LDL: low-density lipoprotein; VLDL: very-low-density lipoprotein.

O'Keeffe LM, *et al. Heart* 2023; 109:674–685. doi: 10.1136/heartjnl-2022-321347

S4 Table: Mean absolute difference in traits between 7y and 25y in males and females

	Original units		SD units	
	Mean absolute difference between 7y and 25y (95% CI)		Mean absolute difference between 7y and 25y (95% CI)	
	Males	Females	Males	Females
	Beta	Beta		
Concentration of chylomicrons and extremely large VLDL particles (mol/l)	-2.95E-11 (-3.48E-11,-2.41E-11)	-7.26E-11 (-7.71E-11,-6.81E-11)	-0.28(-0.33,-0.23)	-0.69(-0.73,-0.65)
Total lipids in chylomicrons and extremely large VLDL (mmol/l)	-1.02E-02 (-1.13E-02,-8.99E-03)	-1.93E-02 (-2.03E-02,-1.83E-02)	-0.43(-0.48,-0.38)	-0.82(-0.86,-0.78)
Phospholipids in chylomicrons and extremely large VLDL (mmol/l)	-1.37E-03 (-1.52E-03,-1.23E-03)	-2.45E-03 (-2.57E-03,-2.32E-03)	-0.48(-0.53,-0.43)	-0.85(-0.9,-0.81)
Total cholesterol in chylomicrons and extremely large VLDL (mmol/l)	-1.53E-03 (-1.72E-03,-1.34E-03)	-2.87E-03 (-3.02E-03,-2.71E-03)	-0.42(-0.47,-0.37)	-0.79(-0.84,-0.75)
Cholesterol esters in chylomicrons and extremely large VLDL (mmol/l)	-6.10E-04 (-7.10E-04,-5.11E-04)	-1.27E-03 (-1.35E-03,-1.19E-03)	-0.34(-0.39,-0.28)	-0.7(-0.74,-0.66)
Free cholesterol in chylomicrons and extremely large VLDL (mmol/l)	-9.13E-04 (-1.01E-03,-8.18E-04)	-1.61E-03 (-1.69E-03,-1.53E-03)	-0.48(-0.53,-0.43)	-0.84(-0.89,-0.8)
Triglycerides in chylomicrons and extremely large VLDL (mmol/l)	-7.28E-03 (-8.13E-03,-6.44E-03)	-1.40E-02 (-1.47E-02,-1.33E-02)	-0.43(-0.47,-0.38)	-0.82(-0.86,-0.78)
Concentration of very large VLDL particles (mol/l)	-1.35E-10 (-1.66E-10,-1.04E-10)	-3.80E-10 (-4.05E-10,-3.54E-10)	-0.23(-0.28,-0.18)	-0.65(-0.7,-0.61)
Total lipids in very large VLDL (mmol/l)	-1.53E-02 (-1.82E-02,-1.23E-02)	-3.88E-02 (-4.12E-02,-3.63E-02)	-0.27(-0.33,-0.22)	-0.69(-0.73,-0.65)
Phospholipids in very large VLDL (mmol/l)	-2.62E-03 (-3.12E-03,-2.12E-03)	-6.29E-03 (-6.70E-03,-5.88E-03)	-0.28(-0.33,-0.23)	-0.67(-0.71,-0.62)
Total cholesterol in very large VLDL (mmol/l)	-4.30E-03 (-4.87E-03,-3.73E-03)	-8.90E-03 (-9.37E-03,-8.44E-03)	-0.4(-0.45,-0.35)	-0.82(-0.87,-0.78)
Cholesterol esters in very large VLDL (mmol/l)	-2.05E-03 (-2.35E-03,-1.75E-03)	-4.52E-03 (-4.76E-03,-4.29E-03)	-0.37(-0.43,-0.32)	-0.82(-0.87,-0.78)
Free cholesterol in very large VLDL (mmol/l)	-2.30E-03 (-2.57E-03,-2.02E-03)	-4.37E-03 (-4.60E-03,-4.14E-03)	-0.43(-0.48,-0.38)	-0.81(-0.86,-0.77)
Triglycerides in very large VLDL (mmol/l)	-8.33E-03 (-1.03E-02,-6.40E-03)	-2.36E-02 (-2.52E-02,-2.20E-02)	-0.23(-0.28,-0.18)	-0.65(-0.7,-0.61)
Concentration of large VLDL particles (mol/l)	1.90E-10 (2.41E-11,3.57E-10)	-1.28E-09 (-1.41E-09,-1.16E-09)	0.07(0.01,0.13)	-0.48(-0.53,-0.43)
Total lipids in large VLDL (mmol/l)	7.76E-04 (-8.96E-03,1.05E-02)	-8.56E-02 (-9.32E-02,-7.81E-02)	0.004(-0.06,0.07)	-0.54(-0.58,-0.49)
Phospholipids in large VLDL (mmol/l)	1.36E-03 (-4.06E-04,3.12E-03)	-1.37E-02 (-1.51E-02,-1.23E-02)	0.05(-0.01,0.11)	-0.48(-0.53,-0.43)
Total cholesterol in large VLDL (mmol/l)	-3.42E-03 (-5.59E-03,-1.25E-03)	-2.25E-02 (-2.42E-02,-2.08E-02)	-0.1(-0.16,-0.03)	-0.63(-0.67,-0.58)
Cholesterol esters in large VLDL (mmol/l)	4.91E-05 (-9.97E-04,1.09E-03)	-9.89E-03 (-1.07E-02,-9.10E-03)	0.002(-0.06,0.07)	-0.59(-0.64,-0.54)
Free cholesterol in large VLDL (mmol/l)	-3.47E-03 (-4.60E-03,-2.33E-03)	-1.26E-02 (-1.35E-02,-1.17E-02)	-0.18(-0.24,-0.12)	-0.65(-0.69,-0.6)
Triglycerides in large VLDL (mmol/l)	2.82E-03 (-3.00E-03,8.64E-03)	-4.95E-02 (-5.40E-02,-4.50E-02)	0.03(-0.03,0.09)	-0.52(-0.57,-0.47)
Concentration of medium VLDL particles (mol/l)	2.06E-09 (1.66E-09,2.47E-09)	-2.33E-09 (-2.63E-09,-2.03E-09)	0.35(0.28,0.42)	-0.4(-0.45,-0.35)
Total lipids in medium VLDL (mmol/l)	4.81E-02 (3.46E-02,6.15E-02)	-9.81E-02 (-1.08E-01,-8.82E-02)	0.24(0.18,0.31)	-0.5(-0.55,-0.45)
Phospholipids in medium VLDL (mmol/l)	8.05E-03 (5.47E-03,1.06E-02)	-1.93E-02 (-2.12E-02,-1.74E-02)	0.21(0.14,0.28)	-0.51(-0.56,-0.46)
Total cholesterol in medium VLDL (mmol/l)	7.18E-03 (3.77E-03,1.06E-02)	-2.54E-02 (-2.80E-02,-2.28E-02)	0.14(0.07,0.21)	-0.5(-0.55,-0.45)
Cholesterol esters in medium VLDL (mmol/l)	6.21E-03 (4.41E-03,8.01E-03)	-9.43E-03 (-1.08E-02,-8.05E-03)	0.22(0.16,0.29)	-0.34(-0.39,-0.29)
Free cholesterol in medium VLDL (mmol/l)	9.50E-04 (-7.32E-04,2.63E-03)	-1.60E-02 (-1.73E-02,-1.47E-02)	0.04(-0.03,0.1)	-0.64(-0.69,-0.59)
Triglycerides in medium VLDL (mmol/l)	3.28E-02 (2.53E-02,4.04E-02)	-5.35E-02 (-5.90E-02,-4.79E-02)	0.29(0.23,0.36)	-0.48(-0.53,-0.43)
Concentration of small VLDL particles (mol/l)	-3.56E-10 (-7.61E-10,4.85E-11)	-4.90E-09 (-5.21E-09,-4.59E-09)	-0.06(-0.13,0.01)	-0.85(-0.9,-0.79)
Total lipids in small VLDL (mmol/l)	-5.76E-02 (-6.55E-02,-4.98E-02)	-1.50E-01 (-1.56E-01,-1.44E-01)	-0.5(-0.57,-0.43)	-1.3(-1.35,-1.24)
Phospholipids in small VLDL (mmol/l)	-1.27E-02 (-1.42E-02,-1.12E-02)	-2.78E-02 (-2.91E-02,-2.66E-02)	-0.55(-0.62,-0.49)	-1.21(-1.27,-1.16)
Total cholesterol in small VLDL (mmol/l)	-4.64E-02 (-4.91E-02,-4.37E-02)	-7.78E-02 (-8.00E-02,-7.56E-02)	-1.09(-1.15,-1.03)	-1.83(-1.88,-1.77)
Cholesterol esters in small VLDL (mmol/l)	-3.29E-02 (-3.47E-02,-3.11E-02)	-5.35E-02 (-5.50E-02,-5.20E-02)	-1.14(-1.2,-1.08)	-1.85(-1.9,-1.8)
Free cholesterol in small VLDL (mmol/l)	-1.35E-02 (-1.46E-02,-1.25E-02)	-2.43E-02 (-2.51E-02,-2.35E-02)	-0.87(-0.94,-0.8)	-1.56(-1.61,-1.51)
Triglycerides in small VLDL (mmol/l)	1.85E-03 (-2.08E-03,5.78E-03)	-4.35E-02 (-4.65E-02,-4.05E-02)	0.03(-0.04,0.1)	-0.74(-0.79,-0.69)
Concentration of very small VLDL particles (mol/l)	-2.00E-09 (-2.33E-09,-1.67E-09)	-3.25E-09 (-3.54E-09,-2.95E-09)	-0.43(-0.51,-0.36)	-0.7(-0.77,-0.64)
Total lipids in very small VLDL (mmol/l)	-1.05E-01 (-1.09E-01,-1.00E-01)	-1.30E-01 (-1.34E-01,-1.26E-01)	-1.44(-1.51,-1.38)	-1.79(-1.85,-1.74)
Phospholipids in very small VLDL (mmol/l)	-1.20E-02 (-1.34E-02,-1.06E-02)	-1.48E-02 (-1.61E-02,-1.35E-02)	-0.49(-0.55,-0.44)	-0.61(-0.67,-0.56)
Total cholesterol in very small VLDL (mmol/l)	-7.94E-02 (-8.18E-02,-7.69E-02)	-9.30E-02 (-9.52E-02,-9.08E-02)	-1.77(-1.83,-1.72)	-2.08(-2.13,-2.03)
Cholesterol esters in very small VLDL (mmol/l)	-5.88E-02 (-6.05E-02,-5.71E-02)	-6.87E-02 (-7.02E-02,-6.72E-02)	-1.92(-1.97,-1.86)	-2.24(-2.29,-2.19)
Free cholesterol in very small VLDL (mmol/l)	-2.06E-02 (-2.14E-02,-1.97E-02)	-2.42E-02 (-2.50E-02,-2.34E-02)	-1.31(-1.36,-1.25)	-1.54(-1.58,-1.49)
Triglycerides in very small VLDL (mmol/l)	-1.44E-02 (-1.56E-02,-1.32E-02)	-2.23E-02 (-2.34E-02,-2.12E-02)	-0.74(-0.8,-0.68)	-1.14(-1.2,-1.09)
Concentration of IDL particles (mol/l)	2.41E-09 (1.46E-09,3.36E-09)	3.00E-09 (2.13E-09,3.87E-09)	0.18(0.11,0.24)	0.22(0.16,0.28)
Total lipids in IDL (mmol/l)	-1.22E-01 (-1.32E-01,-1.12E-01)	-1.31E-01 (-1.40E-01,-1.22E-01)	-0.69(-0.75,-0.64)	-0.74(-0.8,-0.69)

S4 Table: Mean absolute difference in traits between 7y and 25y in males and females

	Original units		SD units	
	Mean absolute difference between 7y and 25y (95% CI)		Mean absolute difference between 7y and 25y (95% CI)	
	Males	Females	Males	Females
Phospholipids in IDL (mmol/l)	-2.06E-02 (-2.32E-02,-1.80E-02)	-1.93E-02 (-2.17E-02,-1.68E-02)	-0.43(-0.49,-0.38)	-0.41(-0.46,-0.35)
Total cholesterol in IDL (mmol/l)	-8.18E-02 (-8.87E-02,-7.50E-02)	-9.53E-02 (-1.02E-01,-8.91E-02)	-0.68(-0.74,-0.63)	-0.8(-0.85,-0.74)
Cholesterol esters in IDL (mmol/l)	-6.02E-02 (-6.52E-02,-5.52E-02)	-7.57E-02 (-8.02E-02,-7.13E-02)	-0.71(-0.77,-0.65)	-0.89(-0.94,-0.84)
Free cholesterol in IDL (mmol/l)	-2.16E-02 (-2.36E-02,-1.96E-02)	-1.96E-02 (-2.15E-02,-1.77E-02)	-0.58(-0.64,-0.53)	-0.53(-0.58,-0.48)
Triglycerides in IDL (mmol/l)	-1.99E-02 (-2.09E-02,-1.88E-02)	-1.62E-02 (-1.73E-02,-1.51E-02)	-0.96(-1.01,-0.9)	-0.78(-0.83,-0.73)
Concentration of large LDL particles (mol/l)	2.21E-08 (2.04E-08,2.39E-08)	2.17E-08 (2.01E-08,2.33E-08)	0.86(0.79,0.92)	0.84(0.78,0.9)
Total lipids in large LDL (mmol/l)	-2.86E-02 (-4.16E-02,-1.57E-02)	-5.12E-02 (-6.31E-02,-3.93E-02)	-0.13(-0.18,-0.07)	-0.23(-0.28,-0.17)
Phospholipids in large LDL (mmol/l)	4.26E-04 (-2.24E-03,3.09E-03)	-3.47E-03 (-5.94E-03,-1.01E-03)	0.01(-0.05,0.07)	-0.08(-0.13,-0.02)
Total cholesterol in large LDL (mmol/l)	-6.51E-03 (-1.61E-02,3.06E-03)	-3.35E-02 (-4.22E-02,-2.49E-02)	-0.04(-0.1,0.02)	-0.2(-0.26,-0.15)
Cholesterol esters in large LDL (mmol/l)	6.79E-04 (-6.64E-03,8.00E-03)	-2.55E-02 (-3.21E-02,-1.90E-02)	0.01(-0.05,0.06)	-0.21(-0.26,-0.15)
Free cholesterol in large LDL (mmol/l)	-7.20E-03 (-9.50E-03,-4.90E-03)	-8.02E-03 (-1.01E-02,-5.90E-03)	-0.17(-0.23,-0.12)	-0.19(-0.25,-0.14)
Triglycerides in large LDL (mmol/l)	-2.24E-02 (-2.34E-02,-2.13E-02)	-1.39E-02 (-1.51E-02,-1.27E-02)	-0.97(-1.01,-0.92)	-0.6(-0.65,-0.55)
Concentration of medium LDL particles (mol/l)	2.30E-08 (2.13E-08,2.46E-08)	1.99E-08 (1.84E-08,2.14E-08)	1.01(0.93,1.08)	0.87(0.81,0.94)
Total lipids in medium LDL (mmol/l)	1.63E-02 (7.76E-03,2.48E-02)	-1.21E-02 (-1.99E-02,-4.39E-03)	0.12(0.06,0.18)	-0.09(-0.14,-0.03)
Phospholipids in medium LDL (mmol/l)	-1.31E-03 (-2.94E-03,3.21E-04)	-4.99E-03 (-6.49E-03,-3.49E-03)	-0.05(-0.11,0.01)	-0.19(-0.25,-0.13)
Total cholesterol in medium LDL (mmol/l)	2.11E-02 (1.46E-02,2.76E-02)	-7.76E-03 (-1.36E-02,-1.92E-03)	0.2(0.14,0.26)	-0.07(-0.13,-0.02)
Cholesterol esters in medium LDL (mmol/l)	2.64E-02 (2.12E-02,3.17E-02)	1.68E-03 (-3.04E-03,6.41E-03)	0.31(0.25,0.37)	0.02(-0.04,0.07)
Free cholesterol in medium LDL (mmol/l)	-5.38E-03 (-6.67E-03,-4.10E-03)	-9.43E-03 (-1.06E-02,-8.27E-03)	-0.28(-0.35,-0.21)	-0.49(-0.55,-0.43)
Triglycerides in medium LDL (mmol/l)	-5.39E-03 (-6.04E-03,-4.75E-03)	2.65E-04 (-4.62E-04,9.91E-04)	-0.35(-0.39,-0.31)	0.02(-0.03,0.06)
Concentration of small LDL particles (mol/l)	1.60E-08 (1.40E-08,1.79E-08)	1.29E-08 (1.12E-08,1.47E-08)	0.64(0.56,0.72)	0.52(0.45,0.59)
Total lipids in small LDL (mmol/l)	-6.26E-03 (-1.19E-02,-6.28E-04)	-2.33E-02 (-2.85E-02,-1.81E-02)	-0.07(-0.13,-0.01)	-0.26(-0.32,-0.2)
Phospholipids in small LDL (mmol/l)	-7.73E-03 (-8.97E-03,-6.49E-03)	-8.17E-03 (-9.36E-03,-6.98E-03)	-0.42(-0.49,-0.35)	-0.45(-0.51,-0.38)
Total cholesterol in small LDL (mmol/l)	2.74E-03 (-1.41E-03,6.88E-03)	-1.38E-02 (-1.76E-02,-1.00E-02)	0.04(-0.02,0.11)	-0.21(-0.27,-0.15)
Cholesterol esters in small LDL (mmol/l)	1.11E-02 (7.80E-03,1.45E-02)	-2.83E-03 (-5.88E-03,2.26E-04)	0.2(0.14,0.26)	-0.05(-0.11,0.004)
Free cholesterol in small LDL (mmol/l)	-8.42E-03 (-9.32E-03,-7.52E-03)	-1.10E-02 (-1.18E-02,-1.01E-02)	-0.7(-0.78,-0.63)	-0.92(-0.98,-0.85)
Triglycerides in small LDL (mmol/l)	-3.06E-03 (-3.49E-03,-2.62E-03)	-1.68E-03 (-2.14E-03,-1.22E-03)	-0.33(-0.37,-0.28)	-0.18(-0.23,-0.13)
Concentration of very large HDL particles (mol/l)	-2.24E-07 (-2.30E-07,-2.17E-07)	-5.89E-08 (-6.64E-08,-5.13E-08)	-1.94(-2,-1.88)	-0.51(-0.58,-0.45)
Total lipids in very large HDL (mmol/l)	-3.56E-01 (-3.63E-01,-3.49E-01)	-1.89E-01 (-1.97E-01,-1.81E-01)	-2.57(-2.62,-2.52)	-1.37(-1.42,-1.31)
Phospholipids in very large HDL (mmol/l)	-1.21E-01 (-1.25E-01,-1.17E-01)	-1.96E-02 (-2.40E-02,-1.53E-02)	-1.61(-1.66,-1.56)	-0.26(-0.32,-0.2)
Total cholesterol in very large HDL (mmol/l)	-2.32E-01 (-2.36E-01,-2.29E-01)	-1.66E-01 (-1.70E-01,-1.62E-01)	-3.54(-3.59,-3.49)	-2.53(-2.59,-2.47)
Cholesterol esters in very large HDL (mmol/l)	-1.80E-01 (-1.83E-01,-1.78E-01)	-1.36E-01 (-1.39E-01,-1.33E-01)	-3.81(-3.86,-3.75)	-2.88(-2.93,-2.82)
Free cholesterol in very large HDL (mmol/l)	-5.09E-02 (-5.19E-02,-4.99E-02)	-2.90E-02 (-3.01E-02,-2.80E-02)	-2.68(-2.74,-2.63)	-1.53(-1.59,-1.47)
Triglycerides in very large HDL (mmol/l)	-9.31E-03 (-9.61E-03,-9.01E-03)	-7.41E-03 (-7.70E-03,-7.12E-03)	-1.8(-1.86,-1.75)	-1.44(-1.49,-1.38)
Concentration of large HDL particles (mol/l)	-2.15E-07 (-2.33E-07,-1.98E-07)	2.50E-07 (2.31E-07,2.69E-07)	-0.94(-1.02,-0.86)	1.09(1.01,1.17)
Total lipids in large HDL (mmol/l)	-2.35E-01 (-2.47E-01,-2.23E-01)	6.63E-02 (5.39E-02,7.88E-02)	-1.35(-1.42,-1.28)	0.38(0.31,0.45)
Phospholipids in large HDL (mmol/l)	-8.91E-02 (-9.43E-02,-8.39E-02)	4.60E-02 (4.04E-02,5.16E-02)	-1.16(-1.23,-1.09)	0.6(0.53,0.67)
Total cholesterol in large HDL (mmol/l)	-1.42E-01 (-1.48E-01,-1.36E-01)	1.59E-02 (9.30E-03,2.25E-02)	-1.47(-1.53,-1.4)	0.16(0.1,0.23)
Cholesterol esters in large HDL (mmol/l)	-1.10E-01 (-1.15E-01,-1.05E-01)	1.08E-02 (5.75E-03,1.59E-02)	-1.45(-1.52,-1.39)	0.14(0.08,0.21)
Free cholesterol in large HDL (mmol/l)	-3.16E-02 (-3.31E-02,-3.02E-02)	5.09E-03 (3.55E-03,6.63E-03)	-1.52(-1.59,-1.44)	0.24(0.17,0.32)
Triglycerides in large HDL (mmol/l)	-3.93E-03 (-4.32E-03,-3.54E-03)	4.60E-03 (4.10E-03,5.11E-03)	-1.13(-1.24,-1.02)	1.32(1.18,1.47)
Concentration of medium HDL particles (mol/l)	3.76E-07 (3.58E-07,3.94E-07)	6.80E-07 (6.60E-07,7.00E-07)	2.23(2.13,2.34)	4.04(3.92,4.16)
Total lipids in medium HDL (mmol/l)	7.71E-04 (-6.79E-03,8.33E-03)	1.40E-01 (1.32E-01,1.49E-01)	0.01(-0.08,0.1)	1.64(1.54,1.74)
Phospholipids in medium HDL (mmol/l)	-2.09E-02 (-2.43E-02,-1.74E-02)	4.81E-02 (4.41E-02,5.22E-02)	-0.45(-0.52,-0.37)	1.03(0.94,1.12)
Total cholesterol in medium HDL (mmol/l)	1.80E-02 (1.36E-02,2.25E-02)	8.35E-02 (7.89E-02,8.82E-02)	0.44(0.34,0.55)	2.06(1.94,2.17)
Cholesterol esters in medium HDL (mmol/l)	1.41E-02 (1.06E-02,1.76E-02)	6.48E-02 (6.11E-02,6.85E-02)	0.42(0.32,0.53)	1.93(1.82,2.04)

O'Keeffe LM, *et al. Heart* 2023; 109:674–685. doi: 10.1136/heartjnl-2022-321347

S4 Table: Mean absolute difference in traits between 7y and 25y in males and females

	Original units		SD units	
	Mean absolute difference between 7y and 25y (95% CI)		Mean absolute difference between 7y and 25y (95% CI)	
	Males	Females	Males	Females
Free cholesterol in medium HDL (mmol/l)	3.93E-03 (2.99E-03,4.87E-03)	1.88E-02 (1.77E-02,1.98E-02)	0.53(0.4,0.65)	2.51(2.37,2.64)
Triglycerides in medium HDL (mmol/l)	9.68E-03 (9.12E-03,1.02E-02)	1.11E-02 (1.06E-02,1.17E-02)	1.22(1.15,1.3)	1.41(1.34,1.48)
Concentration of small HDL particles (mol/l)	6.77E-07 (6.49E-07,7.04E-07)	8.92E-07 (8.60E-07,9.24E-07)	2.68(2.57,2.79)	3.53(3.4,3.66)
Total lipids in small HDL (mmol/l)	5.14E-02 (4.48E-02,5.80E-02)	1.02E-01 (9.47E-02,1.10E-01)	0.73(0.63,0.82)	1.44(1.34,1.55)
Phospholipids in small HDL (mmol/l)	-1.43E-02 (-1.75E-02,-1.11E-02)	1.35E-02 (9.84E-03,1.71E-02)	-0.32(-0.39,-0.25)	0.3(0.22,0.38)
Total cholesterol in small HDL (mmol/l)	6.17E-02 (5.74E-02,6.61E-02)	8.95E-02 (8.48E-02,9.42E-02)	1.39(1.29,1.49)	2.01(1.91,2.12)
Cholesterol esters in small HDL (mmol/l)	7.68E-02 (7.29E-02,8.07E-02)	9.84E-02 (9.43E-02,1.02E-01)	1.93(1.83,2.02)	2.47(2.36,2.57)
Free cholesterol in small HDL (mmol/l)	-1.69E-02 (-1.76E-02,-1.62E-02)	-1.09E-02 (-1.17E-02,-1.01E-02)	-1.77(-1.84,-1.69)	-1.14(-1.22,-1.06)
Triglycerides in small HDL (mmol/l)	7.00E-04 (1.63E-04,1.24E-03)	-1.94E-03 (-2.42E-03,-1.47E-03)	0.08(0.02,0.14)	-0.23(-0.28,-0.17)
Mean diameter for VLDL particles (mm)	2.84E-01 (2.12E-01,3.57E-01)	-5.88E-01 (-6.48E-01,-5.27E-01)	0.22(0.16,0.27)	-0.45(-0.5,-0.41)
Mean diameter for LDL particles (mm)	-5.89E-02 (-6.73E-02,-5.05E-02)	-2.31E-02 (-3.13E-02,-1.49E-02)	-0.57(-0.65,-0.48)	-0.22(-0.3,-0.14)
Mean diameter for HDL particles (mm)	-2.80E-01 (-2.90E-01,-2.70E-01)	-5.72E-02 (-6.65E-02,-4.79E-02)	-1.84(-1.91,-1.78)	-0.38(-0.44,-0.32)
Serum total cholesterol (mmol/l)	-4.74E-01 (-5.11E-01,-4.38E-01)	-3.59E-01 (-3.94E-01,-3.24E-01)	-0.79(-0.85,-0.73)	-0.6(-0.66,-0.54)
Total cholesterol in VLDL (mmol/l)	-1.27E-01 (-1.37E-01,-1.17E-01)	-2.30E-01 (-2.38E-01,-2.22E-01)	-0.83(-0.89,-0.76)	-1.49(-1.54,-1.44)
Remnant cholesterol (non-HDL, non-LDL -cholesterol) (mmol/l)	-2.09E-01 (-2.24E-01,-1.94E-01)	-3.25E-01 (-3.38E-01,-3.13E-01)	-0.87(-0.93,-0.81)	-1.36(-1.41,-1.31)
Total cholesterol in LDL (mmol/l)	1.73E-02 (-2.74E-03,3.74E-02)	-5.51E-02 (-7.32E-02,-3.71E-02)	0.05(-0.01,0.11)	-0.17(-0.22,-0.11)
Total cholesterol in HDL (mmol/l)	-2.85E-01 (-2.99E-01,-2.71E-01)	2.09E-02 (6.63E-03,3.51E-02)	-1.37(-1.44,-1.31)	0.1(0.03,0.17)
Total cholesterol in HDL2 (mmol/l)	-1.30E-01 (-1.41E-01,-1.18E-01)	1.48E-01 (1.36E-01,1.61E-01)	-0.94(-1.02,-0.85)	1.07(0.99,1.16)
Total cholesterol in HDL3 (mmol/l)	-1.54E-01 (-1.57E-01,-1.51E-01)	-1.23E-01 (-1.26E-01,-1.20E-01)	-2.07(-2.11,-2.03)	-1.65(-1.7,-1.61)
Esterified cholesterol (mmol/l)	-3.52E-01 (-3.78E-01,-3.27E-01)	-2.62E-01 (-2.87E-01,-2.37E-01)	-0.82(-0.88,-0.76)	-0.61(-0.67,-0.55)
Free cholesterol (mmol/l)	-1.39E-01 (-1.50E-01,-1.29E-01)	-9.78E-02 (-1.08E-01,-8.75E-02)	-0.77(-0.83,-0.71)	-0.54(-0.6,-0.48)
Serum total triglycerides (mmol/l)	-4.75E-02 (-7.13E-02,-2.37E-02)	-2.30E-01 (-2.49E-01,-2.11E-01)	-0.12(-0.19,-0.06)	-0.61(-0.65,-0.56)
Triglycerides in VLDL (mmol/l)	7.82E-03 (-1.31E-02,2.87E-02)	-2.06E-01 (-2.22E-01,-1.90E-01)	0.02(-0.04,0.09)	-0.62(-0.67,-0.57)
Triglycerides in LDL (mmol/l)	-3.06E-02 (-3.27E-02,-2.85E-02)	-1.52E-02 (-1.75E-02,-1.29E-02)	-0.65(-0.69,-0.6)	-0.32(-0.37,-0.27)
Triglycerides in HDL (mmol/l)	-2.98E-03 (-4.35E-03,-1.61E-03)	6.17E-03 (4.76E-03,7.57E-03)	-0.14(-0.21,-0.08)	0.3(0.23,0.37)
Diacylglycerol (mmol/l)*	-1.97E-03 (-2.57E-03,-1.37E-03)	-2.40E-03 (-3.11E-03,-1.68E-03)	-0.18(-0.24,-0.13)	-0.22(-0.29,-0.16)
Total phosphoglycerides (mmol/l)	-3.76E-01 (-3.91E-01,-3.62E-01)	-1.84E-01 (-2.00E-01,-1.67E-01)	-1.39(-1.44,-1.33)	-0.68(-0.74,-0.61)
Phosphatidylcholine and other cholines (mmol/l)	-3.62E-01 (-3.79E-01,-3.45E-01)	-2.10E-01 (-2.27E-01,-1.93E-01)	-1.32(-1.38,-1.26)	-0.77(-0.83,-0.7)
Total cholines (mmol/l)	-3.85E-01 (-4.02E-01,-3.68E-01)	-1.75E-01 (-1.94E-01,-1.56E-01)	-1.29(-1.35,-1.23)	-0.59(-0.65,-0.52)
Apolipoprotein A-I (g/l)	-1.22E-01 (-1.30E-01,-1.13E-01)	2.63E-02 (1.75E-02,3.52E-02)	-1.09(-1.16,-1.01)	0.23(0.16,0.31)
Apolipoprotein B (g/l)	-1.72E-02 (-2.50E-02,-9.40E-03)	-8.39E-02 (-9.04E-02,-7.73E-02)	-0.14(-0.2,-0.08)	-0.68(-0.73,-0.63)
Total fatty acids (mmol/l)	-1.80E+00 (-1.90E+00,-1.70E+00)	-1.71E+00 (-1.80E+00,-1.61E+00)	-1.13(-1.19,-1.07)	-1.07(-1.13,-1.01)
Fatty acid length*	1.57E-01 (1.33E-01,1.81E-01)	1.05E-01 (7.44E-02,1.35E-01)	0.44(0.38,0.51)	0.3(0.21,0.38)
Estimated degree of unsaturation*	1.72E-02 (1.30E-02,2.15E-02)	2.86E-02 (2.33E-02,3.39E-02)	0.28(0.21,0.36)	0.47(0.39,0.56)
22:6, docosahexaenoic acid (mmol/l)	-7.50E-03 (-9.04E-03,-5.95E-03)	6.54E-03 (4.87E-03,8.21E-03)	-0.28(-0.34,-0.22)	0.24(0.18,0.31)
18:2, linoleic acid (mmol/l)	-5.42E-01 (-5.68E-01,-5.15E-01)	-5.18E-01 (-5.43E-01,-4.93E-01)	-1.23(-1.29,-1.16)	-1.17(-1.23,-1.12)
Conjugated linoleic acid (mmol/l)*	-7.74E-03 (-8.71E-03,-6.78E-03)	-8.89E-03 (-1.03E-02,-7.46E-03)	-0.44(-0.49,-0.38)	-0.5(-0.58,-0.42)
Omega-3 fatty acids (mmol/l)	-3.63E-02 (-4.05E-02,-3.22E-02)	-4.03E-02 (-4.43E-02,-3.63E-02)	-0.53(-0.59,-0.47)	-0.59(-0.65,-0.53)
Omega-6 fatty acids (mmol/l)	-6.34E-01 (-6.63E-01,-6.04E-01)	-5.44E-01 (-5.73E-01,-5.16E-01)	-1.29(-1.35,-1.23)	-1.11(-1.16,-1.05)
Polyunsaturated fatty acids (mmol/l)	-6.70E-01 (-7.02E-01,-6.38E-01)	-5.85E-01 (-6.16E-01,-5.53E-01)	-1.25(-1.31,-1.19)	-1.09(-1.15,-1.03)
Monounsaturated fatty acids; 16:1, 18:1 (mmol/l)	-2.16E-01 (-2.51E-01,-1.80E-01)	-2.61E-01 (-2.93E-01,-2.28E-01)	-0.39(-0.45,-0.32)	-0.47(-0.52,-0.41)
Saturated fatty acids (mmol/l)	-9.22E-01 (-9.61E-01,-8.82E-01)	-8.76E-01 (-9.13E-01,-8.38E-01)	-1.38(-1.43,-1.32)	-1.31(-1.36,-1.25)
Glucose (mmol/l)	-1.68E-01 (-2.02E-01,-1.34E-01)	-2.82E-01 (-3.08E-01,-2.57E-01)	-0.33(-0.39,-0.26)	-0.55(-0.6,-0.5)
Lactate (mmol/l)	-4.06E-01 (-4.33E-01,-3.79E-01)	-4.73E-01 (-5.01E-01,-4.44E-01)	-0.84(-0.9,-0.79)	-0.98(-1.04,-0.92)
Citrate (mmol/l)	2.81E-02 (2.66E-02,2.96E-02)	2.32E-02 (2.18E-02,2.45E-02)	1.12(1.06,1.18)	0.92(0.87,0.98)

S4 Table: Mean absolute difference in traits between 7y and 25y in males and females

	Original units		SD units	
	Mean absolute difference between 7y and 25y (95% CI)		Mean absolute difference between 7y and 25y (95% CI)	
	Males	Females	Males	Females
Alanine (mmol/l)	5.32E-02 (4.95E-02,5.69E-02)	3.97E-02 (3.64E-02,4.30E-02)	0.83(0.77,0.89)	0.62(0.57,0.67)
Glutamine (mmol/l)	-5.55E-02 (-5.97E-02,-5.12E-02)	-1.45E-01 (-1.48E-01,-1.41E-01)	-0.96(-1.03,-0.88)	-2.49(-2.56,-2.43)
Histidine (mmol/l)	-2.10E-02 (-2.16E-02,-2.04E-02)	-2.66E-02 (-2.72E-02,-2.60E-02)	-1.47(-1.51,-1.42)	-1.86(-1.9,-1.81)
Isoleucine (mmol/l)	3.01E-03 (2.08E-03,3.94E-03)	-8.71E-03 (-9.52E-03,-7.90E-03)	0.17(0.12,0.22)	-0.48(-0.53,-0.44)
Leucine (mmol/l)	9.37E-03 (8.52E-03,1.02E-02)	-2.61E-03 (-3.33E-03,-1.89E-03)	0.62(0.57,0.68)	-0.17(-0.22,-0.13)
Valine (mmol/l)	1.53E-02 (1.34E-02,1.73E-02)	-1.17E-02 (-1.34E-02,-9.99E-03)	0.44(0.38,0.49)	-0.33(-0.38,-0.29)
Phenylalanine (mmol/l)	6.10E-03 (5.65E-03,6.55E-03)	4.39E-03 (3.98E-03,4.80E-03)	0.71(0.66,0.76)	0.51(0.46,0.56)
Tyrosine (mmol/l)	-2.07E-02 (-2.15E-02,-2.00E-02)	-2.63E-02 (-2.70E-02,-2.55E-02)	-1.31(-1.35,-1.26)	-1.66(-1.71,-1.61)
Acetate (mmol/l)	-6.45E-03 (-8.73E-03,-4.17E-03)	-1.30E-02 (-1.43E-02,-1.17E-02)	-0.27(-0.36,-0.17)	-0.54(-0.59,-0.49)
Acetoacetate (mmol/l)	-1.91E-02 (-2.10E-02,-1.72E-02)	-2.30E-02 (-2.50E-02,-2.10E-02)	-0.4(-0.44,-0.36)	-0.49(-0.53,-0.44)
3-hydroxybutyrate (mmol/l)	5.34E-02 (4.84E-02,5.84E-02)	5.75E-02 (5.17E-02,6.33E-02)	0.55(0.5,0.6)	0.59(0.53,0.65)
Creatinine (mmol/l)	2.92E-02 (2.87E-02,2.96E-02)	1.68E-02 (1.65E-02,1.71E-02)	4.77(4.7,4.84)	2.75(2.7,2.81)
Albumin (mmol/l)	4.28E-03 (3.99E-03,4.57E-03)	3.09E-04 (4.88E-05,5.70E-04)	1.18(1.1,1.27)	0.09(0.01,0.16)
Glycoprotein acetyls, mainly a1-acid glycoprotein (mmol/l)	6.33E-03 (-3.30E-03,1.60E-02)	-8.46E-03 (-1.67E-02,-1.73E-04)	0.05(-0.02,0.12)	-0.06(-0.12,-0.001)

*These metabolites (diacylglycerol, fatty acid chain length, estimated degree of saturation and conjugated linoleic acid) were not measured at 25y; all models include data only up to aged 18y and values in this table for these traits are at 7y and 18y respectively. HDL: high-density lipoprotein; IDL: intermediate-density lipoprotein; LDL: low-density lipoprotein; VLDL: very-low-density lipoprotein.

O'Keeffe LM, *et al. Heart* 2023; 109:674–685. doi: 10.1136/heartjnl-2022-321347

S5 Table: Mean rate of change in concentration per year in males and females

	Males		Females	
	**Mean change in concentration per year from 7 to 15y/ 18y(95%CI)**	**Mean change in concentration per year from 15y/18y to 25y (95%CI)**	**Mean change in concentration per year from 7 to 15y/ 18y(95%CI)**	**Mean change in concentration per year from 15y/18y to 25y (95%CI)**
Concentration of chylomicrons and extremely large VLDL particles (mol/l)	-3.48E-12 (-4.11E-12,-2.85E-12)	-1.80E-13 (-7.41E-13,3.81E-13)	-6.18E-12 (-6.76E-12,-5.59E-12)	-2.58E-12 (-2.93E-12,-2.22E-12)
Total lipids in chylomicrons and extremely large VLDL (mmol/l)	-7.84E-04 (-9.25E-04,-6.43E-04)	-4.32E-04 (-5.55E-04,-3.10E-04)	-1.35E-03 (-1.49E-03,-1.22E-03)	-9.43E-04 (-1.02E-03,-8.65E-04)
Phospholipids in chylomicrons and extremely large VLDL (mmol/l)	-1.01E-04 (-1.18E-04,-8.35E-05)	-6.28E-05 (-7.81E-05,-4.76E-05)	-1.65E-04 (-1.81E-04,-1.49E-04)	-1.25E-04 (-1.35E-04,-1.16E-04)
Total cholesterol in chylomicrons and extremely large VLDL (mmol/l)	-8.79E-05 (-1.05E-04,-7.06E-05)	-9.34E-05 (-1.21E-04,-6.54E-05)	-1.69E-04 (-1.85E-04,-1.53E-04)	-1.68E-04 (-1.87E-04,-1.50E-04)
Cholesterol esters in chylomicrons and extremely large VLDL (mmol/l)	-3.52E-05 (-4.42E-05,-2.63E-05)	-3.71E-05 (-5.21E-05,-2.21E-05)	-7.15E-05 (-7.97E-05,-6.33E-05)	-8.00E-05 (-9.02E-05,-6.97E-05)
Free cholesterol in chylomicrons and extremely large VLDL (mmol/l)	-6.13E-05 (-7.28E-05,-4.98E-05)	-4.70E-05 (-5.70E-05,-3.70E-05)	-1.03E-04 (-1.14E-04,-9.27E-05)	-8.69E-05 (-9.33E-05,-8.05E-05)
Triglycerides in chylomicrons and extremely large VLDL (mmol/l)	-5.68E-04 (-6.70E-04,-4.65E-04)	-3.05E-04 (-3.92E-04,-2.18E-04)	-1.00E-03 (-1.10E-03,-9.07E-04)	-6.64E-04 (-7.19E-04,-6.08E-04)
Concentration of very large VLDL particles (mol/l)	-1.82E-11 (-2.17E-11,-1.47E-11)	1.20E-12 (-2.10E-12,4.50E-12)	-3.37E-11 (-3.69E-11,-3.05E-11)	-1.22E-11 (-1.43E-11,-1.02E-11)
Total lipids in very large VLDL (mmol/l)	-1.83E-03 (-2.17E-03,-1.49E-03)	-7.20E-05 (-3.93E-04,2.49E-04)	-3.26E-03 (-3.57E-03,-2.94E-03)	-1.41E-03 (-1.61E-03,-1.21E-03)
Phospholipids in very large VLDL (mmol/l)	-3.47E-04 (-4.03E-04,-2.90E-04)	1.69E-05 (-3.64E-05,7.01E-05)	-5.48E-04 (-6.01E-04,-4.96E-04)	-2.12E-04 (-2.45E-04,-1.79E-04)
Total cholesterol in very large VLDL (mmol/l)	-3.16E-04 (-3.82E-04,-2.50E-04)	-1.97E-04 (-2.58E-04,-1.35E-04)	-5.91E-04 (-6.52E-04,-5.30E-04)	-4.64E-04 (-5.03E-04,-4.25E-04)
Cholesterol esters in very large VLDL (mmol/l)	-1.03E-04 (-1.29E-04,-7.68E-05)	-1.53E-04 (-1.97E-04,-1.10E-04)	-2.70E-04 (-2.94E-04,-2.46E-04)	-2.59E-04 (-2.87E-04,-2.31E-04)
Free cholesterol in very large VLDL (mmol/l)	-1.81E-04 (-2.13E-04,-1.48E-04)	-9.48E-05 (-1.24E-04,-6.57E-05)	-2.96E-04 (-3.26E-04,-2.65E-04)	-2.23E-04 (-2.41E-04,-2.04E-04)
Triglycerides in very large VLDL (mmol/l)	-1.16E-03 (-1.38E-03,-9.44E-04)	1.08E-04 (-9.96E-05,3.15E-04)	-2.12E-03 (-2.32E-03,-1.92E-03)	-7.36E-04 (-8.66E-04,-6.06E-04)
Concentration of large VLDL particles (mol/l)	-9.61E-11 (-1.13E-10,-7.95E-11)	1.07E-10 (8.79E-11,1.25E-10)	-1.62E-10 (-1.77E-10,-1.47E-10)	1.72E-12 (-1.01E-11,1.35E-11)
Total lipids in large VLDL (mmol/l)	-5.64E-03 (-6.63E-03,-4.65E-03)	5.10E-03 (4.01E-03,6.19E-03)	-9.56E-03 (-1.05E-02,-8.66E-03)	-1.02E-03 (-1.71E-03,-3.25E-04)
Phospholipids in large VLDL (mmol/l)	-1.14E-03 (-1.31E-03,-9.59E-04)	1.16E-03 (9.63E-04,1.36E-03)	-1.77E-03 (-1.93E-03,-1.61E-03)	5.08E-05 (-7.57E-05,1.77E-04)
Total cholesterol in large VLDL (mmol/l)	-1.40E-03 (-1.62E-03,-1.17E-03)	8.61E-04 (6.18E-04,1.10E-03)	-2.14E-03 (-2.35E-03,-1.94E-03)	-5.93E-04 (-7.48E-04,-4.39E-04)
Cholesterol esters in large VLDL (mmol/l)	-6.43E-04 (-7.48E-04,-5.38E-04)	5.77E-04 (4.59E-04,6.95E-04)	-9.71E-04 (-1.07E-03,-8.74E-04)	-2.36E-04 (-3.13E-04,-1.59E-04)
Free cholesterol in large VLDL (mmol/l)	-7.51E-04 (-8.71E-04,-6.32E-04)	2.82E-04 (1.57E-04,4.08E-04)	-1.17E-03 (-1.28E-03,-1.06E-03)	-3.57E-04 (-4.36E-04,-2.78E-04)
Triglycerides in large VLDL (mmol/l)	-3.11E-03 (-3.70E-03,-2.51E-03)	3.08E-03 (2.43E-03,3.73E-03)	-5.65E-03 (-6.19E-03,-5.11E-03)	-4.76E-04 (-8.90E-04,-6.28E-05)
Concentration of medium VLDL particles (mol/l)	-1.98E-10 (-2.34E-10,-1.61E-10)	4.05E-10 (3.58E-10,4.52E-10)	-3.58E-10 (-3.91E-10,-3.24E-10)	5.89E-11 (2.86E-11,8.91E-11)
Total lipids in medium VLDL (mmol/l)	-6.58E-03 (-7.83E-03,-5.33E-03)	1.12E-02 (9.64E-03,1.27E-02)	-1.17E-02 (-1.28E-02,-1.05E-02)	-5.47E-04 (-1.56E-03,4.70E-04)
Phospholipids in medium VLDL (mmol/l)	-1.58E-03 (-1.81E-03,-1.34E-03)	2.29E-03 (2.00E-03,2.59E-03)	-2.37E-03 (-2.59E-03,-2.14E-03)	-4.23E-05 (-2.39E-04,1.55E-04)
Total cholesterol in medium VLDL (mmol/l)	-2.62E-03 (-2.94E-03,-2.30E-03)	3.13E-03 (2.73E-03,3.52E-03)	-3.21E-03 (-3.51E-03,-2.91E-03)	2.97E-05 (-2.42E-04,3.01E-04)
Cholesterol esters in medium VLDL (mmol/l)	-1.57E-03 (-1.74E-03,-1.40E-03)	2.09E-03 (1.88E-03,2.30E-03)	-1.70E-03 (-1.87E-03,-1.54E-03)	4.65E-04 (3.15E-04,6.14E-04)
Free cholesterol in medium VLDL (mmol/l)	-1.04E-03 (-1.20E-03,-8.85E-04)	1.03E-03 (8.39E-04,1.23E-03)	-1.50E-03 (-1.65E-03,-1.36E-03)	-4.41E-04 (-5.69E-04,-3.12E-04)
Triglycerides in medium VLDL (mmol/l)	-2.37E-03 (-3.08E-03,-1.67E-03)	5.75E-03 (4.88E-03,6.62E-03)	-6.07E-03 (-6.71E-03,-5.43E-03)	-5.43E-04 (-1.10E-03,1.79E-05)
Concentration of small VLDL particles (mol/l)	-2.93E-10 (-3.28E-10,-2.58E-10)	2.21E-10 (1.74E-10,2.68E-10)	-3.78E-10 (-4.13E-10,-3.43E-10)	-2.09E-10 (-2.43E-10,-1.74E-10)
Total lipids in small VLDL (mmol/l)	-3.67E-03 (-4.20E-03,-3.13E-03)	-2.89E-03 (-4.08E-03,-1.69E-03)	-6.28E-03 (-6.83E-03,-5.73E-03)	-1.35E-02 (-1.44E-02,-1.26E-02)
Phospholipids in small VLDL (mmol/l)	-1.38E-03 (-1.51E-03,-1.24E-03)	-1.87E-04 (-3.66E-04,-8.70E-06)	-1.46E-03 (-1.61E-03,-1.32E-03)	-1.79E-03 (-1.94E-03,-1.65E-03)
Total cholesterol in small VLDL (mmol/l)	-1.44E-03 (-1.62E-03,-1.25E-03)	-5.10E-03 (-5.52E-03,-4.68E-03)	-2.22E-03 (-2.42E-03,-2.01E-03)	-8.90E-03 (-9.26E-03,-8.55E-03)
Cholesterol esters in small VLDL (mmol/l)	-6.73E-04 (-7.98E-04,-5.48E-04)	-4.25E-03 (-4.53E-03,-3.97E-03)	-1.28E-03 (-1.42E-03,-1.14E-03)	-6.56E-03 (-6.81E-03,-6.32E-03)
Free cholesterol in small VLDL (mmol/l)	-7.66E-04 (-8.38E-04,-6.95E-04)	-8.52E-04 (-1.01E-03,-6.93E-04)	-9.33E-04 (-1.01E-03,-8.57E-04)	-2.34E-03 (-2.47E-03,-2.21E-03)
Triglycerides in small VLDL (mmol/l)	-2.16E-03 (-2.53E-03,-1.80E-03)	2.13E-03 (1.67E-03,2.58E-03)	-3.32E-03 (-3.67E-03,-2.97E-03)	-1.89E-03 (-2.20E-03,-1.57E-03)
Concentration of very small VLDL particles (mol/l)	-4.17E-10 (-4.42E-10,-3.92E-10)	1.48E-10 (1.08E-10,1.89E-10)	-2.95E-10 (-3.24E-10,-2.66E-10)	-9.82E-11 (-1.34E-10,-6.21E-11)
Total lipids in very small VLDL (mmol/l)	-3.75E-03 (-4.05E-03,-3.45E-03)	-1.06E-02 (-1.13E-02,-9.92E-03)	-3.78E-03 (-4.13E-03,-3.44E-03)	-1.48E-02 (-1.54E-02,-1.41E-02)
Phospholipids in very small VLDL (mmol/l)	-2.10E-03 (-2.22E-03,-1.97E-03)	5.32E-04 (3.61E-04,7.04E-04)	-1.21E-03 (-1.35E-03,-1.06E-03)	-5.70E-04 (-7.29E-04,-4.10E-04)
Total cholesterol in very small VLDL (mmol/l)	-1.17E-03 (-1.37E-03,-9.79E-04)	-1.11E-02 (-1.15E-02,-1.07E-02)	-1.95E-03 (-2.16E-03,-1.75E-03)	-1.19E-02 (-1.23E-02,-1.16E-02)
Cholesterol esters in very small VLDL (mmol/l)	-4.68E-04 (-6.04E-04,-3.33E-04)	-8.95E-03 (-9.21E-03,-8.68E-03)	-1.40E-03 (-1.55E-03,-1.25E-03)	-8.89E-03 (-9.14E-03,-8.64E-03)
Free cholesterol in very small VLDL (mmol/l)	-7.13E-04 (-7.79E-04,-6.46E-04)	-2.13E-03 (-2.25E-03,-2.00E-03)	-5.56E-04 (-6.28E-04,-4.84E-04)	-3.02E-03 (-3.13E-03,-2.90E-03)
Triglycerides in very small VLDL (mmol/l)	-9.96E-04 (-1.09E-03,-9.04E-04)	-5.76E-04 (-7.61E-04,-3.90E-04)	-8.71E-04 (-9.73E-04,-7.69E-04)	-2.12E-03 (-2.29E-03,-1.96E-03)
Concentration of IDL particles (mol/l)	-1.10E-09 (-1.16E-09,-1.05E-09)	2.43E-09 (2.28E-09,2.57E-09)	-6.93E-10 (-7.56E-10,-6.29E-10)	1.77E-09 (1.63E-09,1.91E-09)

O'Keeffe LM, *et al. Heart* 2023; 109:674–685. doi: 10.1136/heartjnl-2022-321347

S5 Table: Mean rate of change in concentration per year in males and females

	Males		Females	
	**Mean change in concentration per year from 7 to 15y/ 18y(95%CI)**	**Mean change in concentration per year from 15y/18y to 25y (95%CI)**	**Mean change in concentration per year from 7 to 15y/ 18y(95%CI)**	**Mean change in concentration per year from 15y/18y to 25y (95%CI)**
Total lipids in IDL (mmol/l)	-1.33E-02 (-1.40E-02,-1.26E-02)	4.04E-03 (2.48E-03,5.60E-03)	-8.22E-03 (-9.02E-03,-7.42E-03)	-6.77E-03 (-8.27E-03,-5.27E-03)
Phospholipids in IDL (mmol/l)	-3.56E-03 (-3.74E-03,-3.37E-03)	3.08E-03 (2.69E-03,3.48E-03)	-1.86E-03 (-2.08E-03,-1.64E-03)	1.97E-04 (-1.87E-04,5.81E-04)
Total cholesterol in IDL (mmol/l)	-8.18E-03 (-8.65E-03,-7.71E-03)	1.36E-03 (2.93E-04,2.43E-03)	-5.82E-03 (-6.35E-03,-5.30E-03)	-5.21E-03 (-6.22E-03,-4.20E-03)
Cholesterol esters in IDL (mmol/l)	-5.44E-03 (-5.78E-03,-5.10E-03)	-6.70E-05 (-8.45E-04,7.11E-04)	-4.35E-03 (-4.72E-03,-3.98E-03)	-4.65E-03 (-5.37E-03,-3.92E-03)
Free cholesterol in IDL (mmol/l)	-2.75E-03 (-2.89E-03,-2.60E-03)	1.44E-03 (1.13E-03,1.74E-03)	-1.47E-03 (-1.64E-03,-1.31E-03)	-5.66E-04 (-8.60E-04,-2.72E-04)
Triglycerides in IDL (mmol/l)	-1.59E-03 (-1.69E-03,-1.50E-03)	-3.91E-04 (-5.57E-04,-2.26E-04)	-5.13E-04 (-6.26E-04,-3.99E-04)	-1.76E-03 (-1.94E-03,-1.57E-03)
Concentration of large LDL particles (mol/l)	-1.98E-09 (-2.08E-09,-1.88E-09)	7.32E-09 (7.04E-09,7.60E-09)	-1.01E-09 (-1.13E-09,-8.88E-10)	5.46E-09 (5.21E-09,5.71E-09)
Total lipids in large LDL (mmol/l)	-1.80E-02 (-1.89E-02,-1.72E-02)	2.83E-02 (2.63E-02,3.03E-02)	-9.53E-03 (-1.06E-02,-8.49E-03)	8.93E-03 (7.04E-03,1.08E-02)
Phospholipids in large LDL (mmol/l)	-3.44E-03 (-3.62E-03,-3.26E-03)	6.37E-03 (5.95E-03,6.79E-03)	-1.78E-03 (-1.99E-03,-1.57E-03)	2.68E-03 (2.29E-03,3.07E-03)
Total cholesterol in large LDL (mmol/l)	-1.27E-02 (-1.34E-02,-1.21E-02)	2.23E-02 (2.08E-02,2.38E-02)	-7.23E-03 (-7.97E-03,-6.48E-03)	7.66E-03 (6.27E-03,9.04E-03)
Cholesterol esters in large LDL (mmol/l)	-9.62E-03 (-1.01E-02,-9.13E-03)	1.77E-02 (1.66E-02,1.89E-02)	-5.63E-03 (-6.19E-03,-5.07E-03)	6.07E-03 (5.02E-03,7.12E-03)
Free cholesterol in large LDL (mmol/l)	-3.13E-03 (-3.29E-03,-2.96E-03)	4.53E-03 (4.18E-03,4.89E-03)	-1.59E-03 (-1.78E-03,-1.41E-03)	1.59E-03 (1.25E-03,1.92E-03)
Triglycerides in large LDL (mmol/l)	-1.87E-03 (-1.97E-03,-1.77E-03)	-2.99E-04 (-4.57E-04,-1.40E-04)	-5.02E-04 (-6.25E-04,-3.79E-04)	-1.39E-03 (-1.58E-03,-1.21E-03)
Concentration of medium LDL particles (mol/l)	-1.68E-09 (-1.78E-09,-1.59E-09)	6.92E-09 (6.66E-09,7.18E-09)	-8.88E-10 (-9.97E-10,-7.80E-10)	4.94E-09 (4.71E-09,5.17E-09)
Total lipids in medium LDL (mmol/l)	-1.11E-02 (-1.16E-02,-1.05E-02)	2.30E-02 (2.16E-02,2.43E-02)	-6.03E-03 (-6.68E-03,-5.37E-03)	9.03E-03 (7.79E-03,1.03E-02)
Phospholipids in medium LDL (mmol/l)	-3.39E-03 (-3.53E-03,-3.24E-03)	2.86E-03 (2.66E-03,3.07E-03)	-1.98E-03 (-2.14E-03,-1.82E-03)	1.20E-03 (1.02E-03,1.39E-03)
Total cholesterol in medium LDL (mmol/l)	-7.78E-03 (-8.19E-03,-7.36E-03)	1.78E-02 (1.67E-02,1.88E-02)	-4.61E-03 (-5.08E-03,-4.13E-03)	7.15E-03 (6.22E-03,8.08E-03)
Cholesterol esters in medium LDL (mmol/l)	-6.13E-03 (-6.48E-03,-5.79E-03)	1.56E-02 (1.48E-02,1.65E-02)	-3.60E-03 (-3.99E-03,-3.20E-03)	6.88E-03 (6.13E-03,7.62E-03)
Free cholesterol in medium LDL (mmol/l)	-1.65E-03 (-1.72E-03,-1.57E-03)	2.12E-03 (1.92E-03,2.33E-03)	-1.00E-03 (-1.09E-03,-9.18E-04)	2.70E-04 (8.05E-05,4.60E-04)
Triglycerides in medium LDL (mmol/l)	-1.01E-03 (-1.08E-03,-9.46E-04)	9.56E-04 (8.66E-04,1.05E-03)	-1.26E-04 (-2.04E-04,-4.81E-05)	2.75E-04 (1.72E-04,3.78E-04)
Concentration of small LDL particles (mol/l)	-2.15E-09 (-2.25E-09,-2.05E-09)	6.61E-09 (6.29E-09,6.92E-09)	-1.30E-09 (-1.42E-09,-1.18E-09)	4.54E-09 (4.26E-09,4.82E-09)
Total lipids in small LDL (mmol/l)	-7.41E-03 (-7.77E-03,-7.06E-03)	1.25E-02 (1.17E-02,1.34E-02)	-4.45E-03 (-4.86E-03,-4.03E-03)	4.28E-03 (3.45E-03,5.10E-03)
Phospholipids in small LDL (mmol/l)	-2.55E-03 (-2.65E-03,-2.45E-03)	1.41E-03 (1.25E-03,1.57E-03)	-1.63E-03 (-1.74E-03,-1.52E-03)	5.42E-04 (3.96E-04,6.88E-04)
Total cholesterol in small LDL (mmol/l)	-4.96E-03 (-5.22E-03,-4.70E-03)	9.55E-03 (8.90E-03,1.02E-02)	-3.04E-03 (-3.34E-03,-2.74E-03)	3.27E-03 (2.67E-03,3.86E-03)
Cholesterol esters in small LDL (mmol/l)	-3.92E-03 (-4.14E-03,-3.70E-03)	9.04E-03 (8.52E-03,9.56E-03)	-2.31E-03 (-2.57E-03,-2.05E-03)	3.76E-03 (3.29E-03,4.23E-03)
Free cholesterol in small LDL (mmol/l)	-1.05E-03 (-1.09E-03,-9.98E-04)	5.14E-04 (3.69E-04,6.58E-04)	-7.20E-04 (-7.74E-04,-6.67E-04)	-5.06E-04 (-6.41E-04,-3.72E-04)
Triglycerides in small LDL (mmol/l)	-8.23E-04 (-8.79E-04,-7.68E-04)	3.92E-04 (3.44E-04,4.41E-04)	-3.99E-04 (-4.59E-04,-3.39E-04)	1.68E-04 (1.21E-04,2.14E-04)
Concentration of very large HDL particles (mol/l)	-8.69E-09 (-9.30E-09,-8.07E-09)	-1.71E-08 (-1.79E-08,-1.63E-08)	-9.70E-10 (-1.65E-09,-2.95E-10)	-5.68E-09 (-6.59E-09,-4.77E-09)
Total lipids in very large HDL (mmol/l)	-9.16E-03 (-9.91E-03,-8.41E-03)	-3.14E-02 (-3.23E-02,-3.06E-02)	-4.20E-04 (-1.24E-03,3.96E-04)	-2.06E-02 (-2.16E-02,-1.97E-02)
Phospholipids in very large HDL (mmol/l)	-5.18E-03 (-5.57E-03,-4.79E-03)	-8.79E-03 (-9.25E-03,-8.34E-03)	5.53E-04 (1.28E-04,9.77E-04)	-2.67E-03 (-3.19E-03,-2.15E-03)
Total cholesterol in very large HDL (mmol/l)	-6.97E-03 (-7.26E-03,-6.67E-03)	-2.59E-02 (-2.65E-02,-2.54E-02)	-3.79E-03 (-4.11E-03,-3.48E-03)	-2.07E-02 (-2.13E-02,-2.01E-02)
Cholesterol esters in very large HDL (mmol/l)	-5.07E-03 (-5.29E-03,-4.85E-03)	-2.08E-02 (-2.12E-02,-2.03E-02)	-3.05E-03 (-3.28E-03,-2.81E-03)	-1.71E-02 (-1.76E-02,-1.67E-02)
Free cholesterol in very large HDL (mmol/l)	-1.25E-03 (-1.35E-03,-1.15E-03)	-4.54E-03 (-4.65E-03,-4.43E-03)	-1.81E-04 (-2.92E-04,-7.01E-05)	-3.07E-03 (-3.19E-03,-2.94E-03)
Triglycerides in very large HDL (mmol/l)	-3.76E-04 (-4.08E-04,-3.44E-04)	-7.01E-04 (-7.34E-04,-6.67E-04)	-2.01E-04 (-2.34E-04,-1.69E-04)	-6.44E-04 (-6.77E-04,-6.12E-04)
Concentration of large HDL particles (mol/l)	-2.10E-08 (-2.19E-08,-2.01E-08)	2.66E-09 (5.96E-11,5.26E-09)	1.59E-09 (5.56E-10,2.62E-09)	3.88E-08 (3.58E-08,4.18E-08)
Total lipids in large HDL (mmol/l)	-1.54E-02 (-1.61E-02,-1.47E-02)	-1.09E-02 (-1.26E-02,-9.22E-03)	1.13E-03 (3.56E-04,1.90E-03)	8.99E-03 (7.06E-03,1.09E-02)
Phospholipids in large HDL (mmol/l)	-7.07E-03 (-7.37E-03,-6.77E-03)	-1.88E-03 (-2.65E-03,-1.12E-03)	7.68E-04 (4.20E-04,1.12E-03)	6.25E-03 (5.38E-03,7.13E-03)
Total cholesterol in large HDL (mmol/l)	-7.97E-03 (-8.34E-03,-7.59E-03)	-9.03E-03 (-9.94E-03,-8.13E-03)	4.95E-04 (7.30E-05,9.17E-04)	1.74E-03 (7.20E-04,2.76E-03)
Cholesterol esters in large HDL (mmol/l)	-6.23E-03 (-6.52E-03,-5.93E-03)	-6.95E-03 (-7.64E-03,-6.26E-03)	4.74E-04 (1.41E-04,8.07E-04)	9.32E-04 (1.49E-04,1.71E-03)
Free cholesterol in large HDL (mmol/l)	-1.74E-03 (-1.82E-03,-1.66E-03)	-2.08E-03 (-2.30E-03,-1.86E-03)	2.04E-05 (-6.94E-05,1.10E-04)	8.11E-04 (5.71E-04,1.05E-03)
Triglycerides in large HDL (mmol/l)	-3.73E-04 (-3.89E-04,-3.57E-04)	2.85E-05 (-3.51E-05,9.21E-05)	-1.38E-04 (-1.55E-04,-1.20E-04)	1.02E-03 (9.38E-04,1.10E-03)
Concentration of medium HDL particles (mol/l)	-1.43E-08 (-1.51E-08,-1.34E-08)	8.88E-08 (8.59E-08,9.17E-08)	5.23E-09 (4.16E-09,6.29E-09)	1.04E-07 (1.01E-07,1.07E-07)
Total lipids in medium HDL (mmol/l)	-1.09E-02 (-1.15E-02,-1.04E-02)	9.80E-03 (8.86E-03,1.07E-02)	-7.53E-04 (-1.42E-03,-9.00E-05)	1.63E-02 (1.52E-02,1.73E-02)
Phospholipids in medium HDL (mmol/l)	-5.56E-03 (-5.86E-03,-5.26E-03)	2.63E-03 (2.21E-03,3.04E-03)	2.76E-04 (-8.66E-05,6.38E-04)	5.10E-03 (4.62E-03,5.59E-03)

O'Keeffe LM, *et al. Heart* 2023; 109:674–685. doi: 10.1136/heartjnl-2022-321347

S5 Table: Mean rate of change in concentration per year in males and females

	Males		Females	
	**Mean change in concentration per year from 7 to 15y/ 18y(95%CI)**	**Mean change in concentration per year from 15y/18y to 25y (95%CI)**	**Mean change in concentration per year from 7 to 15y/ 18y(95%CI)**	**Mean change in concentration per year from 15y/18y to 25y (95%CI)**
Total cholesterol in medium HDL (mmol/l)	-3.00E-03 (-3.21E-03,-2.79E-03)	8.50E-03 (7.79E-03,9.22E-03)	1.13E-03 (8.83E-04,1.37E-03)	1.19E-02 (1.11E-02,1.26E-02)
Cholesterol esters in medium HDL (mmol/l)	-2.29E-03 (-2.47E-03,-2.12E-03)	6.55E-03 (5.99E-03,7.12E-03)	1.05E-03 (8.50E-04,1.25E-03)	8.87E-03 (8.26E-03,9.48E-03)
Free cholesterol in medium HDL (mmol/l)	-7.09E-04 (-7.47E-04,-6.71E-04)	1.95E-03 (1.80E-03,2.11E-03)	7.82E-05 (3.33E-05,1.23E-04)	2.98E-03 (2.82E-03,3.15E-03)
Triglycerides in medium HDL (mmol/l)	-5.78E-04 (-6.28E-04,-5.28E-04)	1.59E-03 (1.52E-03,1.66E-03)	-3.63E-04 (-4.16E-04,-3.11E-04)	1.56E-03 (1.50E-03,1.63E-03)
Concentration of small HDL particles (mol/l)	-3.34E-08 (-3.53E-08,-3.15E-08)	1.05E-07 (1.01E-07,1.08E-07)	-1.66E-08 (-1.87E-08,-1.44E-08)	1.14E-07 (1.10E-07,1.18E-07)
Total lipids in small HDL (mmol/l)	-8.08E-03 (-8.61E-03,-7.56E-03)	1.28E-02 (1.19E-02,1.37E-02)	3.58E-04 (-2.85E-04,1.00E-03)	1.05E-02 (9.48E-03,1.15E-02)
Phospholipids in small HDL (mmol/l)	-2.08E-03 (-2.39E-03,-1.77E-03)	2.58E-04 (-1.29E-04,6.45E-04)	5.88E-06 (-3.48E-04,3.60E-04)	1.49E-03 (1.04E-03,1.94E-03)
Total cholesterol in small HDL (mmol/l)	-5.40E-03 (-5.70E-03,-5.09E-03)	1.17E-02 (1.11E-02,1.22E-02)	6.93E-04 (3.54E-04,1.03E-03)	9.33E-03 (8.73E-03,9.92E-03)
Cholesterol esters in small HDL (mmol/l)	-4.66E-03 (-4.93E-03,-4.39E-03)	1.27E-02 (1.22E-02,1.32E-02)	5.09E-04 (2.21E-04,7.97E-04)	1.05E-02 (9.96E-03,1.10E-02)
Free cholesterol in small HDL (mmol/l)	-7.00E-04 (-7.50E-04,-6.50E-04)	-1.53E-03 (-1.65E-03,-1.42E-03)	9.54E-05 (3.21E-05,1.59E-04)	-1.99E-03 (-2.12E-03,-1.85E-03)
Triglycerides in small HDL (mmol/l)	-4.05E-04 (-4.60E-04,-3.50E-04)	4.38E-04 (3.76E-04,5.00E-04)	-2.64E-04 (-3.22E-04,-2.07E-04)	1.89E-05 (-3.56E-05,7.33E-05)
Mean diameter for VLDL particles (mm)	-1.92E-02 (-2.79E-02,-1.06E-02)	4.87E-02 (4.05E-02,5.69E-02)	-7.13E-02 (-7.90E-02,-6.37E-02)	-1.88E-03 (-8.08E-03,4.32E-03)
Mean diameter for LDL particles (mm)	1.53E-02 (1.45E-02,1.61E-02)	-2.01E-02 (-2.12E-02,-1.90E-02)	9.00E-03 (8.26E-03,9.74E-03)	-1.06E-02 (-1.16E-02,-9.52E-03)
Mean diameter for HDL particles (mm)	-1.01E-02 (-1.10E-02,-9.31E-03)	-2.21E-02 (-2.32E-02,-2.09E-02)	5.30E-04 (-3.42E-04,1.40E-03)	-6.83E-03 (-7.95E-03,-5.71E-03)
Serum total cholesterol (mmol/l)	-5.89E-02 (-6.12E-02,-5.65E-02)	2.88E-02 (2.31E-02,3.45E-02)	-2.90E-02 (-3.18E-02,-2.63E-02)	-6.59E-03 (-1.22E-02,-9.76E-04)
Total cholesterol in VLDL (mmol/l)	-5.24E-03 (-5.95E-03,-4.54E-03)	-1.16E-02 (-1.31E-02,-1.01E-02)	-9.10E-03 (-9.81E-03,-8.40E-03)	-2.17E-02 (-2.28E-02,-2.05E-02)
Remnant cholesterol (non-HDL, non-LDL -cholesterol) (mmol/l)	-1.34E-02 (-1.44E-02,-1.24E-02)	-1.02E-02 (-1.26E-02,-7.89E-03)	-1.49E-02 (-1.60E-02,-1.38E-02)	-2.69E-02 (-2.89E-02,-2.49E-02)
Total cholesterol in LDL (mmol/l)	-2.55E-02 (-2.68E-02,-2.42E-02)	4.96E-02 (4.64E-02,5.27E-02)	-1.49E-02 (-1.64E-02,-1.34E-02)	1.81E-02 (1.52E-02,2.10E-02)
Total cholesterol in HDL (mmol/l)	-2.00E-02 (-2.08E-02,-1.92E-02)	-1.09E-02 (-1.29E-02,-8.83E-03)	6.93E-04 (-2.76E-04,1.66E-03)	2.20E-03 (-2.14E-05,4.43E-03)
Total cholesterol in HDL2 (mmol/l)	-1.15E-02 (-1.21E-02,-1.10E-02)	-4.88E-04 (-2.31E-03,1.33E-03)	1.68E-03 (1.05E-03,2.32E-03)	2.17E-02 (1.97E-02,2.36E-02)
Total cholesterol in HDL3 (mmol/l)	-8.59E-03 (-8.89E-03,-8.30E-03)	-9.92E-03 (-1.04E-02,-9.48E-03)	-1.31E-03 (-1.67E-03,-9.47E-04)	-1.81E-02 (-1.87E-02,-1.76E-02)
Esterified cholesterol (mmol/l)	-5.48E-02 (-5.71E-02,-5.25E-02)	9.56E-03 (6.42E-03,1.27E-02)	-2.90E-02 (-3.15E-02,-2.64E-02)	-3.32E-03 (-6.29E-03,-3.42E-04)
Free cholesterol (mmol/l)	-1.63E-02 (-1.70E-02,-1.56E-02)	6.63E-03 (4.94E-03,8.31E-03)	-7.04E-03 (-7.89E-03,-6.20E-03)	-3.39E-03 (-5.08E-03,-1.70E-03)
Serum total triglycerides (mmol/l)	-1.84E-02 (-2.08E-02,-1.60E-02)	1.11E-02 (8.35E-03,1.38E-02)	-2.20E-02 (-2.43E-02,-1.98E-02)	-5.99E-03 (-7.88E-03,-4.10E-03)
Triglycerides in VLDL (mmol/l)	-1.06E-02 (-1.27E-02,-8.54E-03)	1.03E-02 (7.92E-03,1.27E-02)	-1.91E-02 (-2.10E-02,-1.72E-02)	-5.93E-03 (-7.48E-03,-4.37E-03)
Triglycerides in LDL (mmol/l)	-3.48E-03 (-3.69E-03,-3.28E-03)	1.29E-03 (9.87E-04,1.60E-03)	-8.64E-04 (-1.11E-03,-6.19E-04)	-9.47E-04 (-1.29E-03,-6.02E-04)
Triglycerides in HDL (mmol/l)	-1.77E-03 (-1.90E-03,-1.64E-03)	1.24E-03 (1.08E-03,1.40E-03)	-1.12E-03 (-1.26E-03,-9.79E-04)	1.68E-03 (1.51E-03,1.84E-03)
Diacylglycerol (mmol/l)*	-3.33E-04 (-4.11E-04,-2.56E-04)	2.32E-04 (6.66E-06,4.56E-04)	-4.10E-04 (-4.93E-04,-3.27E-04)	4.18E-04 (1.70E-04,6.66E-04)
Total phosphoglycerides (mmol/l)	-4.28E-02 (-4.43E-02,-4.13E-02)	-3.76E-03 (-5.47E-03,-2.05E-03)	-2.07E-02 (-2.25E-02,-1.88E-02)	-2.05E-03 (-3.99E-03,-1.04E-04)
Phosphatidylcholine and other cholines (mmol/l)	-2.65E-02 (-2.77E-02,-2.53E-02)	-1.17E-02 (-1.43E-02,-9.09E-03)	-5.41E-03 (-6.88E-03,-3.93E-03)	-2.51E-02 (-2.78E-02,-2.23E-02)
Total cholines (mmol/l)	-4.19E-02 (-4.36E-02,-4.02E-02)	-5.52E-03 (-7.60E-03,-3.45E-03)	-1.83E-02 (-2.02E-02,-1.63E-02)	-3.20E-03 (-5.43E-03,-9.79E-04)
Apolipoprotein A-I (g/l)	-1.33E-02 (-1.38E-02,-1.29E-02)	4.18E-03 (2.91E-03,5.44E-03)	-2.12E-03 (-2.65E-03,-1.58E-03)	8.27E-03 (6.85E-03,9.68E-03)
Apolipoprotein B (g/l)	-1.01E-02 (-1.08E-02,-9.34E-03)	7.02E-03 (6.09E-03,7.96E-03)	-9.85E-03 (-1.06E-02,-9.12E-03)	-5.59E-04 (-1.31E-03,1.94E-04)
Total fatty acids (mmol/l)	-2.09E-01 (-2.19E-01,-2.00E-01)	-1.36E-02 (-2.53E-02,-1.99E-03)	-1.35E-01 (-1.46E-01,-1.25E-01)	-6.94E-02 (-8.00E-02,-5.88E-02)
Fatty acid length*	3.08E-02 (2.72E-02,3.43E-02)	-2.97E-02 (-4.08E-02,-1.86E-02)	2.07E-02 (1.74E-02,2.41E-02)	-1.80E-03 (-1.19E-02,8.33E-03)
Estimated degree of unsaturation*	4.04E-03 (3.45E-03,4.62E-03)	-5.02E-03 (-6.99E-03,-3.06E-03)	5.45E-03 (4.87E-03,6.02E-03)	-4.94E-03 (-6.66E-03,-3.22E-03)
22:6, docosahexaenoic acid (mmol/l)	-3.04E-03 (-3.21E-03,-2.87E-03)	1.87E-03 (1.67E-03,2.06E-03)	-1.23E-03 (-1.43E-03,-1.02E-03)	1.82E-03 (1.61E-03,2.03E-03)
18:2, linoleic acid (mmol/l)	-6.69E-02 (-6.93E-02,-6.44E-02)	-7.25E-04 (-3.92E-03,2.47E-03)	-4.15E-02 (-4.41E-02,-3.89E-02)	-2.07E-02 (-2.35E-02,-1.79E-02)
Conjugated linoleic acid (mmol/l)*	-8.57E-04 (-1.02E-03,-6.90E-04)	-2.96E-04 (-7.78E-04,1.85E-04)	-1.03E-03 (-1.20E-03,-8.63E-04)	-5.71E-05 (-5.17E-04,4.03E-04)
Omega-3 fatty acids (mmol/l)	-7.65E-03 (-8.11E-03,-7.20E-03)	2.77E-03 (2.26E-03,3.27E-03)	-4.66E-03 (-5.14E-03,-4.18E-03)	-3.36E-04 (-8.13E-04,1.41E-04)
Omega-6 fatty acids (mmol/l)	-7.41E-02 (-7.68E-02,-7.14E-02)	-4.53E-03 (-8.04E-03,-1.03E-03)	-4.32E-02 (-4.61E-02,-4.02E-02)	-2.21E-02 (-2.53E-02,-1.88E-02)
Polyunsaturated fatty acids (mmol/l)	-8.18E-02 (-8.47E-02,-7.88E-02)	-1.77E-03 (-5.63E-03,2.09E-03)	-4.79E-02 (-5.11E-02,-4.47E-02)	-2.24E-02 (-2.60E-02,-1.88E-02)
Monounsaturated fatty acids; 16:1, 18:1 (mmol/l)	-1.73E-02 (-2.00E-02,-1.45E-02)	-4.25E-03 (-9.70E-03,1.19E-03)	-6.00E-03 (-8.91E-03,-3.09E-03)	-3.24E-02 (-3.73E-02,-2.76E-02)
Saturated fatty acids (mmol/l)	-1.04E-01 (-1.08E-01,-1.00E-01)	-9.71E-03 (-1.43E-02,-5.09E-03)	-7.91E-02 (-8.35E-02,-7.47E-02)	-2.70E-02 (-3.12E-02,-2.28E-02)

O'Keeffe LM, *et al. Heart* 2023; 109:674–685. doi: 10.1136/heartjnl-2022-321347

S5 Table: Mean rate of change in concentration per year in males and females

	Males		Females	
	**Mean change in concentration per year from 7 to 15y/ 18y(95%CI)**	**Mean change in concentration per year from 15y/18y to 25y (95%CI)**	**Mean change in concentration per year from 7 to 15y/ 18y(95%CI)**	**Mean change in concentration per year from 15y/18y to 25y (95%CI)**
Glucose (mmol/l)	2.00E-02 (1.66E-02,2.34E-02)	-3.64E-02 (-3.97E-02,-3.31E-02)	1.16E-02 (8.45E-03,1.48E-02)	-4.17E-02 (-4.40E-02,-3.94E-02)
Lactate (mmol/l)	-2.29E-02 (-2.68E-02,-1.91E-02)	-2.47E-02 (-2.84E-02,-2.10E-02)	-2.65E-02 (-3.06E-02,-2.24E-02)	-2.90E-02 (-3.25E-02,-2.54E-02)
Citrate (mmol/l)	-2.49E-03 (-2.62E-03,-2.36E-03)	9.25E-03 (9.01E-03,9.48E-03)	-3.34E-03 (-3.46E-03,-3.22E-03)	9.99E-03 (9.78E-03,1.02E-02)
Alanine (mmol/l)	-4.59E-03 (-4.91E-03,-4.27E-03)	1.73E-02 (1.68E-02,1.78E-02)	-5.20E-03 (-5.52E-03,-4.88E-03)	1.62E-02 (1.57E-02,1.66E-02)
Glutamine (mmol/l)	3.92E-03 (3.63E-03,4.21E-03)	-1.64E-02 (-1.71E-02,-1.58E-02)	-3.80E-03 (-4.12E-03,-3.48E-03)	-1.71E-02 (-1.77E-02,-1.65E-02)
Histidine (mmol/l)	-1.34E-04 (-2.01E-04,-6.67E-05)	-3.26E-03 (-3.36E-03,-3.15E-03)	-7.73E-04 (-8.44E-04,-7.02E-04)	-3.01E-03 (-3.11E-03,-2.92E-03)
Isoleucine (mmol/l)	-1.16E-04 (-2.02E-04,-2.98E-05)	7.14E-04 (5.88E-04,8.39E-04)	-1.13E-03 (-1.21E-03,-1.05E-03)	6.18E-04 (5.32E-04,7.04E-04)
Leucine (mmol/l)	1.97E-04 (1.27E-04,2.68E-04)	1.20E-03 (1.08E-03,1.32E-03)	-8.51E-04 (-9.18E-04,-7.83E-04)	1.12E-03 (1.04E-03,1.21E-03)
Valine (mmol/l)	1.99E-03 (1.76E-03,2.22E-03)	-6.33E-05 (-2.67E-04,1.41E-04)	-6.33E-04 (-8.48E-04,-4.17E-04)	-7.36E-04 (-9.00E-04,-5.72E-04)
Phenylalanine (mmol/l)	-5.33E-04 (-5.73E-04,-4.94E-04)	1.99E-03 (1.93E-03,2.06E-03)	-7.01E-04 (-7.40E-04,-6.62E-04)	2.02E-03 (1.97E-03,2.07E-03)
Tyrosine (mmol/l)	-1.07E-03 (-1.16E-03,-9.73E-04)	-1.35E-03 (-1.42E-03,-1.29E-03)	-1.52E-03 (-1.62E-03,-1.43E-03)	-1.57E-03 (-1.63E-03,-1.50E-03)
Acetate (mmol/l)	-2.12E-03 (-2.28E-03,-1.97E-03)	1.17E-03 (8.98E-04,1.44E-03)	-2.47E-03 (-2.62E-03,-2.33E-03)	7.48E-04 (6.27E-04,8.69E-04)
Acetoacetate (mmol/l)	-1.11E-03 (-1.23E-03,-9.95E-04)		-2.22E-03 (-2.46E-03,-1.99E-03)	
3-hydroxybutyrate (mmol/l)	1.17E-03 (6.35E-04,1.71E-03)	6.75E-03 (5.89E-03,7.61E-03)	2.90E-03 (2.24E-03,3.57E-03)	4.26E-03 (3.26E-03,5.26E-03)
Creatinine (mmol/l)	3.34E-03 (3.30E-03,3.38E-03)	-1.27E-03 (-1.34E-03,-1.19E-03)	2.26E-03 (2.23E-03,2.30E-03)	-1.35E-03 (-1.40E-03,-1.29E-03)
Albumin (mmol/l)	1.01E-04 (8.04E-05,1.21E-04)	5.28E-04 (4.80E-04,5.76E-04)	-1.92E-04 (-2.14E-04,-1.71E-04)	4.04E-04 (3.61E-04,4.48E-04)
Glycoprotein acetyls, mainly a1-acid glycoprotein (mmol/l)	-3.32E-03 (-4.01E-03,-2.63E-03)	7.13E-03 (5.68E-03,8.58E-03)	-5.11E-04 (-1.22E-03,2.00E-04)	-4.73E-04 (-1.68E-03,7.35E-04)

*These metabolites (diacylglycerol, fatty acid chain length, estimated degree of saturation and conjugated linoleic acid) were not measured at 25y; all models include data only up to aged 18y. HDL: high-density lipoprotein; IDL: intermediate-density lipoprotein; LDL: low-density lipoprotein; VLDL: very-low-density lipoprotein.

O'Keeffe LM, *et al. Heart* 2023; 109:674–685. doi: 10.1136/heartjnl-2022-321347

S6 Table: Mean absolute difference in traits in SD units between 7y and 25y in males and females comparing i)findings from multilevel models implemented here to ii)linear regression of traits at 7y and 25y

	7y		25y	
	Mean absolute sex difference in SD units (95% CI)		Mean absolute sex difference in SD units (95% CI)	
	MLM	Regression	MLM	Regression
Concentration of chylomicrons and extremely large VLDL particles (mol/l)	0.16(0.11,0.21)	0.14(0.09,0.2)	-0.39(-0.45,-0.32)	-0.39(-0.46,-0.31)
Total lipids in chylomicrons and extremely large VLDL (mmol/l)	0.15(0.09,0.2)	0.13(0.08,0.19)	-0.39(-0.46,-0.32)	-0.38(-0.46,-0.31)
Phospholipids in chylomicrons and extremely large VLDL (mmol/l)	0.15(0.09,0.2)	0.13(0.08,0.19)	-0.36(-0.43,-0.29)	-0.35(-0.43,-0.28)
Total cholesterol in chylomicrons and extremely large VLDL (mmol/l)	0.16(0.11,0.22)	0.14(0.09,0.2)	-0.3(-0.37,-0.23)	-0.31(-0.39,-0.24)
Cholesterol esters in chylomicrons and extremely large VLDL (mmol/l)	0.16(0.11,0.22)	0.15(0.1,0.2)	-0.26(-0.33,-0.19)	-0.28(-0.35,-0.2)
Free cholesterol in chylomicrons and extremely large VLDL (mmol/l)	0.15(0.09,0.2)	0.13(0.08,0.19)	-0.35(-0.42,-0.28)	-0.35(-0.42,-0.27)
Triglycerides in chylomicrons and extremely large VLDL (mmol/l)	0.15(0.09,0.2)	0.13(0.08,0.18)	-0.41(-0.48,-0.34)	-0.4(-0.48,-0.33)
Concentration of very large VLDL particles (mol/l)	0.14(0.08,0.19)	0.12(0.07,0.17)	-0.4(-0.47,-0.33)	-0.4(-0.47,-0.32)
Total lipids in very large VLDL (mmol/l)	0.14(0.08,0.19)	0.12(0.07,0.17)	-0.4(-0.46,-0.33)	-0.39(-0.47,-0.32)
Phospholipids in very large VLDL (mmol/l)	0.14(0.09,0.2)	0.13(0.08,0.18)	-0.35(-0.42,-0.28)	-0.36(-0.43,-0.28)
Total cholesterol in very large VLDL (mmol/l)	0.17(0.11,0.22)	0.15(0.1,0.2)	-0.38(-0.45,-0.31)	-0.37(-0.45,-0.29)
Cholesterol esters in very large VLDL (mmol/l)	0.18(0.12,0.23)	0.15(0.1,0.2)	-0.38(-0.44,-0.31)	-0.39(-0.46,-0.31)
Free cholesterol in very large VLDL (mmol/l)	0.16(0.11,0.21)	0.15(0.09,0.2)	-0.35(-0.42,-0.28)	-0.35(-0.43,-0.27)
Triglycerides in very large VLDL (mmol/l)	0.12(0.07,0.18)	0.11(0.06,0.16)	-0.41(-0.48,-0.34)	-0.41(-0.48,-0.33)
Concentration of large VLDL particles (mol/l)	0.14(0.08,0.19)	0.12(0.07,0.17)	-0.43(-0.5,-0.37)	-0.45(-0.52,-0.37)
Total lipids in large VLDL (mmol/l)	0.14(0.09,0.2)	0.12(0.07,0.18)	-0.43(-0.5,-0.37)	-0.44(-0.52,-0.37)
Phospholipids in large VLDL (mmol/l)	0.15(0.09,0.2)	0.13(0.08,0.18)	-0.4(-0.47,-0.34)	-0.41(-0.49,-0.34)
Total cholesterol in large VLDL (mmol/l)	0.16(0.11,0.22)	0.15(0.1,0.2)	-0.4(-0.47,-0.34)	-0.41(-0.49,-0.34)
Cholesterol esters in large VLDL (mmol/l)	0.18(0.13,0.24)	0.17(0.12,0.22)	-0.42(-0.49,-0.36)	-0.44(-0.51,-0.36)
Free cholesterol in large VLDL (mmol/l)	0.15(0.09,0.2)	0.13(0.08,0.18)	-0.38(-0.45,-0.31)	-0.39(-0.46,-0.31)
Triglycerides in large VLDL (mmol/l)	0.13(0.08,0.18)	0.11(0.06,0.16)	-0.45(-0.52,-0.38)	-0.46(-0.53,-0.38)
Concentration of medium VLDL particles (mol/l)	0.18(0.13,0.23)	0.16(0.11,0.21)	-0.49(-0.56,-0.43)	-0.52(-0.59,-0.44)
Total lipids in medium VLDL (mmol/l)	0.19(0.14,0.24)	0.17(0.12,0.22)	-0.49(-0.55,-0.42)	-0.51(-0.58,-0.43)
Phospholipids in medium VLDL (mmol/l)	0.2(0.15,0.26)	0.19(0.13,0.24)	-0.46(-0.52,-0.39)	-0.49(-0.56,-0.41)
Total cholesterol in medium VLDL (mmol/l)	0.23(0.18,0.29)	0.22(0.17,0.28)	-0.36(-0.43,-0.3)	-0.39(-0.46,-0.31)
Cholesterol esters in medium VLDL (mmol/l)	0.25(0.2,0.31)	0.25(0.19,0.3)	-0.28(-0.35,-0.22)	-0.31(-0.39,-0.24)
Free cholesterol in medium VLDL (mmol/l)	0.2(0.14,0.25)	0.18(0.13,0.23)	-0.43(-0.5,-0.37)	-0.46(-0.53,-0.38)
Triglycerides in medium VLDL (mmol/l)	0.16(0.11,0.21)	0.14(0.09,0.19)	-0.55(-0.61,-0.48)	-0.56(-0.63,-0.49)
Concentration of small VLDL particles (mol/l)	0.25(0.2,0.31)	0.24(0.19,0.29)	-0.42(-0.48,-0.35)	-0.45(-0.52,-0.38)
Total lipids in small VLDL (mmol/l)	0.29(0.24,0.35)	0.28(0.23,0.33)	-0.41(-0.47,-0.35)	-0.43(-0.5,-0.36)

S6 Table: Mean absolute difference in traits in SD units between 7y and 25y in males and females comparing i)findings from multilevel models implemented here to ii)linear regression of traits at 7y and 25y

	7y		25y	
	Mean absolute sex difference in SD units (95% CI)		Mean absolute sex difference in SD units (95% CI)	
	MLM	Regression	MLM	Regression
Phospholipids in small VLDL (mmol/l)	0.27(0.22,0.32)	0.26(0.21,0.31)	-0.31(-0.37,-0.25)	-0.34(-0.41,-0.27)
Total cholesterol in small VLDL (mmol/l)	0.37(0.32,0.42)	0.36(0.3,0.41)	-0.3(-0.36,-0.23)	-0.31(-0.38,-0.24)
Cholesterol esters in small VLDL (mmol/l)	0.39(0.34,0.44)	0.37(0.32,0.42)	-0.27(-0.33,-0.21)	-0.28(-0.35,-0.21)
Free cholesterol in small VLDL (mmol/l)	0.3(0.24,0.35)	0.29(0.24,0.34)	-0.32(-0.38,-0.25)	-0.34(-0.41,-0.27)
Triglycerides in small VLDL (mmol/l)	0.21(0.15,0.26)	0.19(0.14,0.24)	-0.49(-0.56,-0.43)	-0.53(-0.6,-0.46)
Concentration of very small VLDL particles (mol/l)	0.39(0.34,0.44)	0.39(0.34,0.44)	0.08(0.01,0.14)	0.03(-0.04,0.1)
Total lipids in very small VLDL (mmol/l)	0.39(0.34,0.44)	0.39(0.34,0.44)	0.03(-0.03,0.09)	0.04(-0.03,0.11)
Phospholipids in very small VLDL (mmol/l)	0.36(0.31,0.41)	0.37(0.32,0.42)	0.2(0.14,0.26)	0.15(0.08,0.22)
Total cholesterol in very small VLDL (mmol/l)	0.32(0.27,0.37)	0.31(0.25,0.36)	0.02(-0.05,0.08)	0.04(-0.03,0.12)
Cholesterol esters in very small VLDL (mmol/l)	0.35(0.3,0.4)	0.32(0.27,0.37)	0.02(-0.04,0.09)	0.06(-0.01,0.13)
Free cholesterol in very small VLDL (mmol/l)	0.23(0.18,0.28)	0.24(0.19,0.29)	0.01(-0.06,0.07)	0.01(-0.06,0.08)
Triglycerides in very small VLDL (mmol/l)	0.28(0.22,0.33)	0.29(0.24,0.34)	-0.11(-0.18,-0.05)	-0.14(-0.22,-0.07)
Concentration of IDL particles (mol/l)	0.3(0.25,0.36)	0.32(0.27,0.37)	0.23(0.17,0.29)	0.21(0.14,0.28)
Total lipids in IDL (mmol/l)	0.32(0.27,0.37)	0.33(0.28,0.39)	0.22(0.16,0.28)	0.2(0.13,0.27)
Phospholipids in IDL (mmol/l)	0.27(0.22,0.32)	0.29(0.24,0.34)	0.26(0.2,0.32)	0.24(0.17,0.31)
Total cholesterol in IDL (mmol/l)	0.32(0.27,0.37)	0.32(0.27,0.38)	0.17(0.11,0.23)	0.15(0.08,0.23)
Cholesterol esters in IDL (mmol/l)	0.33(0.28,0.38)	0.33(0.28,0.38)	0.12(0.06,0.18)	0.11(0.04,0.18)
Free cholesterol in IDL (mmol/l)	0.26(0.21,0.31)	0.28(0.23,0.33)	0.28(0.22,0.34)	0.26(0.19,0.33)
Triglycerides in IDL (mmol/l)	0.25(0.2,0.3)	0.29(0.24,0.35)	0.43(0.37,0.49)	0.4(0.34,0.47)
Concentration of large LDL particles (mol/l)	0.26(0.21,0.31)	0.28(0.23,0.33)	0.17(0.1,0.23)	0.13(0.06,0.2)
Total lipids in large LDL (mmol/l)	0.28(0.23,0.34)	0.31(0.25,0.36)	0.15(0.09,0.21)	0.12(0.04,0.19)
Phospholipids in large LDL (mmol/l)	0.29(0.24,0.34)	0.31(0.26,0.36)	0.16(0.1,0.23)	0.13(0.06,0.2)
Total cholesterol in large LDL (mmol/l)	0.28(0.23,0.34)	0.3(0.25,0.35)	0.1(0.04,0.16)	0.06(-0.01,0.13)
Cholesterol esters in large LDL (mmol/l)	0.29(0.24,0.34)	0.31(0.26,0.36)	0.06(0.01,0.12)	0.02(-0.05,0.09)
Free cholesterol in large LDL (mmol/l)	0.26(0.21,0.31)	0.28(0.23,0.33)	0.21(0.15,0.27)	0.17(0.1,0.24)
Triglycerides in large LDL (mmol/l)	0.18(0.12,0.23)	0.23(0.18,0.28)	0.63(0.57,0.69)	0.6(0.54,0.67)
Concentration of medium LDL particles (mol/l)	0.24(0.19,0.3)	0.27(0.22,0.32)	0.07(0.01,0.13)	0.03(-0.04,0.1)
Total lipids in medium LDL (mmol/l)	0.27(0.22,0.33)	0.3(0.24,0.35)	0.06(-0.01,0.12)	0.01(-0.06,0.08)
Phospholipids in medium LDL (mmol/l)	0.3(0.25,0.36)	0.32(0.27,0.37)	0.12(0.06,0.18)	0.04(-0.03,0.11)
Total cholesterol in medium LDL (mmol/l)	0.27(0.22,0.32)	0.29(0.24,0.34)	-0.05(-0.07,0.06)	-0.05(-0.12,0.02)
Cholesterol esters in medium LDL (mmol/l)	0.27(0.22,0.32)	0.29(0.23,0.34)	-0.02(-0.08,0.05)	-0.06(-0.14,0.01)

O'Keeffe LM, *et al. Heart* 2023; 109:674–685. doi: 10.1136/heartjnl-2022-321347

S6 Table: Mean absolute difference in traits in SD units between 7y and 25y in males and females comparing i)findings from multilevel models implemented here to ii)linear regression of traits at 7y and 25y

	7y		25y	
	Mean absolute sex difference in SD units (95% CI)		Mean absolute sex difference in SD units (95% CI)	
	MLM	Regression	MLM	Regression
Free cholesterol in medium LDL (mmol/l)	0.27(0.22,0.32)	0.29(0.24,0.34)	0.04(-0.02,0.1)	0.01(-0.06,0.08)
Triglycerides in medium LDL (mmol/l)	0.11(0.06,0.17)	0.17(0.12,0.22)	0.7(0.64,0.75)	0.65(0.59,0.72)
Concentration of small LDL particles (mol/l)	0.22(0.17,0.27)	0.24(0.19,0.29)	0.06(0.002,0.12)	0.02(-0.05,0.09)
Total lipids in small LDL (mmol/l)	0.26(0.2,0.31)	0.28(0.22,0.33)	0.05(-0.01,0.11)	0.01(-0.06,0.08)
Phospholipids in small LDL (mmol/l)	0.25(0.19,0.3)	0.26(0.21,0.32)	0.15(0.09,0.21)	0.08(0.01,0.15)
Total cholesterol in small LDL (mmol/l)	0.26(0.2,0.31)	0.27(0.22,0.33)	-0.04 (-0.06,0.06)	-0.04(-0.11,0.03)
Cholesterol esters in small LDL (mmol/l)	0.25(0.2,0.3)	0.27(0.22,0.32)	-0.05 (-0.06,0.06)	-0.05(-0.12,0.02)
Free cholesterol in small LDL (mmol/l)	0.23(0.18,0.28)	0.25(0.19,0.3)	0.01(-0.05,0.07)	0.01(-0.07,0.07)
Triglycerides in small LDL (mmol/l)	0.17(0.12,0.22)	0.19(0.14,0.24)	0.42(0.35,0.48)	0.33(0.26,0.4)
Concentration of very large HDL particles (mol/l)	-0.1(-0.15,-0.05)	-0.05(-0.1,0.0008)	0.82(0.77,0.87)	0.85(0.79,0.91)
Total lipids in very large HDL (mmol/l)	-0.11(-0.17,-0.06)	-0.07(-0.12,-0.02)	0.8(0.75,0.85)	0.85(0.79,0.91)
Phospholipids in very large HDL (mmol/l)	-0.11(-0.16,-0.06)	-0.06(-0.11,-0.005)	0.87(0.81,0.92)	0.88(0.81,0.94)
Total cholesterol in very large HDL (mmol/l)	-0.13(-0.18,-0.07)	-0.09(-0.14,-0.04)	0.72(0.67,0.78)	0.79(0.73,0.85)
Cholesterol esters in very large HDL (mmol/l)	-0.13(-0.18,-0.08)	-0.1(-0.15,-0.05)	0.68(0.62,0.74)	0.76(0.7,0.82)
Free cholesterol in very large HDL (mmol/l)	-0.11(-0.16,-0.05)	-0.07(-0.12,-0.01)	0.8(0.75,0.85)	0.86(0.8,0.92)
Triglycerides in very large HDL (mmol/l)	0.11(0.05,0.16)	0.13(0.08,0.18)	0.42(0.36,0.48)	0.44(0.37,0.51)
Concentration of large HDL particles (mol/l)	-0.19(-0.24,-0.14)	-0.12(-0.17,-0.07)	0.86(0.8,0.91)	0.89(0.82,0.95)
Total lipids in large HDL (mmol/l)	-0.18(-0.24,-0.13)	-0.12(-0.17,-0.06)	0.85(0.8,0.91)	0.88(0.82,0.94)
Phospholipids in large HDL (mmol/l)	-0.17(-0.23,-0.12)	-0.1(-0.16,-0.05)	0.86(0.81,0.92)	0.89(0.83,0.95)
Total cholesterol in large HDL (mmol/l)	-0.2(-0.25,-0.14)	-0.13(-0.19,-0.08)	0.83(0.78,0.89)	0.87(0.81,0.93)
Cholesterol esters in large HDL (mmol/l)	-0.2(-0.25,-0.15)	-0.14(-0.19,-0.09)	0.83(0.78,0.89)	0.87(0.8,0.93)
Free cholesterol in large HDL (mmol/l)	-0.17(-0.22,-0.12)	-0.11(-0.17,-0.06)	0.83(0.78,0.89)	0.87(0.81,0.93)
Triglycerides in large HDL (mmol/l)	0.07(0.02,0.13)	0.12(0.07,0.17)	0.76(0.71,0.81)	0.8(0.74,0.86)
Concentration of medium HDL particles (mol/l)	-0.18(-0.23,-0.12)	-0.1(-0.15,-0.05)	0.62(0.56,0.68)	0.61(0.55,0.68)
Total lipids in medium HDL (mmol/l)	-0.17(-0.23,-0.12)	-0.11(-0.16,-0.06)	0.64(0.59,0.7)	0.61(0.54,0.67)
Phospholipids in medium HDL (mmol/l)	-0.17(-0.23,-0.12)	-0.11(-0.16,-0.05)	0.7(0.64,0.75)	0.66(0.6,0.73)
Total cholesterol in medium HDL (mmol/l)	-0.2(-0.26,-0.15)	-0.14(-0.19,-0.09)	0.54(0.48,0.6)	0.54(0.48,0.61)
Cholesterol esters in medium HDL (mmol/l)	-0.22(-0.27,-0.16)	-0.16(-0.21,-0.11)	0.52(0.46,0.58)	0.53(0.46,0.59)
Free cholesterol in medium HDL (mmol/l)	-0.12(-0.18,-0.07)	-0.06(-0.11,-0.004)	0.6(0.54,0.66)	0.61(0.54,0.67)
Triglycerides in medium HDL (mmol/l)	0.16(0.11,0.21)	0.17(0.12,0.23)	0.24(0.18,0.31)	0.19(0.12,0.26)
Concentration of small HDL particles (mol/l)	-0.11(-0.17,-0.06)	-0.08(-0.13,-0.03)	0.26(0.2,0.32)	0.16(0.1,0.23)

O'Keeffe LM, *et al. Heart* 2023; 109:674–685. doi: 10.1136/heartjnl-2022-321347

S6 Table: Mean absolute difference in traits in SD units between 7y and 25y in males and females comparing i)findings from multilevel models implemented here to ii)linear regression of traits at 7y and 25y

	7y		25y	
	Mean absolute sex difference in SD units (95% CI)		Mean absolute sex difference in SD units (95% CI)	
	MLM	Regression	MLM	Regression
Total lipids in small HDL (mmol/l)	-0.14(-0.19,-0.08)	-0.08(-0.13,-0.02)	0.25(0.19,0.31)	0.17(0.1,0.23)
Phospholipids in small HDL (mmol/l)	-0.17(-0.22,-0.11)	-0.15(-0.2,-0.09)	0.27(0.21,0.33)	0.27(0.2,0.33)
Total cholesterol in small HDL (mmol/l)	-0.07(-0.13,-0.02)	-0.01(-0.06,0.05)	0.24(0.18,0.3)	0.09(0.02,0.16)
Cholesterol esters in small HDL (mmol/l)	-0.02(-0.08,0.04)	0.04(-0.01,0.09)	0.23(0.17,0.29)	0.06(-0.01,0.13)
Free cholesterol in small HDL (mmol/l)	-0.27(-0.32,-0.21)	-0.2(-0.26,-0.15)	0.22(0.16,0.28)	0.22(0.16,0.29)
Triglycerides in small HDL (mmol/l)	0.16(0.1,0.21)	0.16(0.11,0.21)	-0.14(-0.2,-0.07)	-0.21(-0.28,-0.14)
Mean diameter for VLDL particles (mm)	0.1(0.04,0.15)	0.07(0.02,0.13)	-0.64(-0.71,-0.58)	-0.65(-0.72,-0.58)
Mean diameter for LDL particles (mm)	0.09(0.04,0.15)	0.06(0.01,0.12)	0.25(0.19,0.31)	0.36(0.29,0.42)
Mean diameter for HDL particles (mm)	-0.12(-0.17,-0.06)	-0.07(-0.12,-0.01)	0.84(0.78,0.89)	0.86(0.8,0.92)
Serum total cholesterol (mmol/l)	0.24(0.18,0.29)	0.27(0.21,0.32)	0.32(0.26,0.38)	0.31(0.24,0.37)
Total cholesterol in VLDL (mmol/l)	0.33(0.28,0.38)	0.31(0.26,0.36)	-0.29(-0.35,-0.22)	-0.29(-0.37,-0.22)
Remnant cholesterol (non-HDL, non-LDL -cholesterol) (mmol/l)	0.37(0.32,0.42)	0.36(0.31,0.41)	-0.09(-0.15,-0.03)	-0.1(-0.17,-0.03)
Total cholesterol in LDL (mmol/l)	0.28(0.22,0.33)	0.29(0.24,0.35)	0.05(-0.01,0.11)	0.05(-0.07,0.08)
Total cholesterol in HDL (mmol/l)	-0.19(-0.24,-0.14)	-0.12(-0.17,-0.07)	0.78(0.73,0.84)	0.81(0.75,0.87)
Total cholesterol in HDL2 (mmol/l)	-0.21(-0.27,-0.16)	-0.15(-0.2,-0.1)	0.8(0.75,0.85)	0.83(0.76,0.89)
Total cholesterol in HDL3 (mmol/l)	-0.12(-0.18,-0.07)	-0.06(-0.11,-0.005)	0.61(0.55,0.68)	0.51(0.44,0.57)
Esterified cholesterol (mmol/l)	0.23(0.18,0.28)	0.25(0.2,0.31)	0.33(0.27,0.39)	0.28(0.21,0.35)
Free cholesterol (mmol/l)	0.25(0.19,0.3)	0.28(0.22,0.33)	0.37(0.31,0.43)	0.35(0.29,0.42)
Serum total triglycerides (mmol/l)	0.19(0.14,0.24)	0.18(0.13,0.23)	-0.29(-0.36,-0.22)	-0.32(-0.4,-0.25)
Triglycerides in VLDL (mmol/l)	0.17(0.11,0.22)	0.15(0.1,0.2)	-0.47(-0.54,-0.41)	-0.49(-0.56,-0.42)
Triglycerides in LDL (mmol/l)	0.16(0.1,0.21)	0.2(0.15,0.26)	0.61(0.55,0.67)	0.57(0.51,0.64)
Triglycerides in HDL (mmol/l)	0.17(0.11,0.22)	0.19(0.13,0.24)	0.45(0.38,0.51)	0.42(0.36,0.49)
Diacylglycerol (mmol/l)*	0.09(0.04,0.15)	0.08(0.03,0.13)	0.1(0.03,0.18)	0.11(0.04,0.18)
Total phosphoglycerides (mmol/l)	0.08(0.03,0.14)	0.12(0.07,0.18)	0.65(0.6,0.71)	0.63(0.57,0.7)
Phosphatidylcholine and other cholines (mmol/l)	0.05(-0.01,0.1)	0.1(0.05,0.16)	0.47(0.41,0.53)	0.46(0.4,0.53)
Total cholines (mmol/l)	0.1(0.05,0.15)	0.14(0.09,0.19)	0.61(0.56,0.67)	0.57(0.51,0.63)
Apolipoprotein A-I (g/l)	-0.07(-0.13,-0.02)	-0.01(-0.06,0.04)	0.68(0.62,0.73)	0.69(0.62,0.75)
Apolipoprotein B (g/l)	0.36(0.31,0.41)	0.35(0.3,0.4)	-0.15(-0.21,-0.08)	-0.19(-0.26,-0.12)
Total fatty acids (mmol/l)	0.23(0.18,0.29)	0.25(0.2,0.31)	0.24(0.18,0.3)	0.2(0.13,0.27)
Fatty acid length*	0.01(-0.04,0.07)	-0.01(-0.06,0.04)	0.02(-0.05,0.1)	0.01(-0.06,0.08)
Estimated degree of unsaturation*	-0.07(-0.12,-0.01)	-0.05(-0.1,0.01)	0.12(0.04,0.19)	0.12(0.05,0.19)

S6 Table: Mean absolute difference in traits in SD units between 7y and 25y in males and females comparing i)findings from multilevel models implemented here to ii)linear regression of traits at 7y and 25y

	7y		25y	
	Mean absolute sex difference in SD units (95% CI)		Mean absolute sex difference in SD units (95% CI)	
	MLM	Regression	MLM	Regression
22:6, docosahexaenoic acid (mmol/l)	0.19(0.14,0.24)	0.23(0.18,0.28)	0.59(0.53,0.65)	0.54(0.48,0.61)
18:2, linoleic acid (mmol/l)	0.23(0.18,0.28)	0.26(0.21,0.31)	0.23(0.17,0.29)	0.17(0.1,0.24)
Conjugated linoleic acid (mmol/l)*	0.12(0.06,0.17)	0.12(0.07,0.17)	0.1(0.03,0.18)	0.1(0.03,0.17)
Omega-3 fatty acids (mmol/l)	0.17(0.11,0.22)	0.19(0.14,0.24)	0.09(0.03,0.16)	0.06(-0.01,0.13)
Omega-6 fatty acids (mmol/l)	0.22(0.17,0.27)	0.25(0.2,0.31)	0.32(0.26,0.38)	0.28(0.21,0.35)
Polyunsaturated fatty acids (mmol/l)	0.22(0.17,0.28)	0.26(0.2,0.31)	0.3(0.24,0.37)	0.26(0.19,0.33)
Monounsaturated fatty acids; 16:1, 18:1 (mmol/l)	0.24(0.19,0.29)	0.25(0.2,0.31)	0.14(0.07,0.2)	0.13(0.05,0.2)
Saturated fatty acids (mmol/l)	0.17(0.11,0.22)	0.19(0.14,0.24)	0.22(0.16,0.29)	0.19(0.12,0.26)
Glucose (mmol/l)	-0.15(-0.21,-0.09)	-0.15(-0.21,-0.1)	-0.42(-0.49,-0.35)	-0.41(-0.48,-0.33)
Lactate (mmol/l)	0.12(0.07,0.18)	0.12(0.07,0.17)	-0.02(-0.08,0.05)	0.01(-0.05,0.08)
Citrate (mmol/l)	0.12(0.07,0.18)	0.12(0.07,0.18)	-0.08(-0.14,-0.01)	-0.1(-0.17,-0.03)
Alanine (mmol/l)	0.07(0.01,0.12)	0.03(-0.02,0.09)	-0.16(-0.23,-0.1)	-0.19(-0.26,-0.12)
Glutamine (mmol/l)	0.44(0.39,0.49)	0.35(0.3,0.4)	-0.83(-0.89,-0.77)	-0.8(-0.87,-0.74)
Histidine (mmol/l)	0.17(0.12,0.21)	0.14(0.09,0.18)	-0.4(-0.47,-0.33)	-0.33(-0.4,-0.26)
Isoleucine (mmol/l)	0.1(0.05,0.16)	0.06(0.01,0.11)	-0.83(-0.9,-0.77)	-0.83(-0.9,-0.77)
Leucine (mmol/l)	-0.01(-0.07,0.04)	-0.06(-0.11,-0.004)	-0.92(-0.98,-0.86)	-0.93(-0.99,-0.86)
Valine (mmol/l)	0.06(-0.004,0.12)	0.02(-0.03,0.08)	-0.85(-0.91,-0.79)	-0.83(-0.89,-0.76)
Phenylalanine (mmol/l)	-0.03(-0.09,0.02)	-0.05(-0.1,0.01)	-0.32(-0.38,-0.25)	-0.35(-0.41,-0.28)
Tyrosine (mmol/l)	0.01(-0.05,0.07)	-0.01(-0.06,0.05)	-0.55(-0.62,-0.49)	-0.52(-0.58,-0.45)
Acetate (mmol/l)	0.04(-0.02,0.1)	0.03(-0.02,0.08)	-0.16(-0.22,-0.1)	-0.17(-0.24,-0.1)
Acetoacetate (mmol/l)	0.04(-0.01,0.09)	0.08(0.03,0.13)	-0.2(-0.31,-0.08)	-0.11(-0.18,-0.04)
3-hydroxybutyrate (mmol/l)	0.12(0.07,0.18)	0.14(0.09,0.2)	0.18(0.12,0.25)	0.16(0.1,0.22)
Creatinine (mmol/l)	0.1(0.05,0.15)	-0.02(-0.07,0.03)	-1.25(-1.3,-1.2)	-1.24(-1.3,-1.18)
Albumin (mmol/l)	0.3(0.24,0.35)	0.23(0.18,0.28)	-0.52(-0.58,-0.45)	-0.53(-0.6,-0.46)
Glycoprotein acetyls, mainly a1-acid glycoprotein (mmol/l)	0.23(0.18,0.29)	0.24(0.19,0.29)	0.1(0.04,0.17)	0.08(0.01,0.15)

*These metabolites (diacylglycerol, fatty acid chain length, estimated degree of saturation and conjugated linoleic acid) were not measured at 25y; all models include data only up to aged 18y and values in this table for these traits are at 7y and 18y respectively. HDL: high-density lipoprotein; IDL: intermediate-density lipoprotein; LDL: low-density lipoprotein; MLM; multilevel model; VLDL: very-low-density lipoprotein.

O'Keeffe LM, *et al. Heart* 2023; 109:674–685. doi: 10.1136/heartjnl-2022-321347

S2 Table: Characteristics of offspring included in analyses compared to those excluded due to missing sex, cardiometabolic trait data or attrition from the cohort

	Female participants included n=3,909*	Female participants excluded	N excluded females	Male participants included n=3,717*	Male participants excluded	N excluded males
	n (%)	n (%)	n	n (%)	n (%)	n
**Non-white ethnicity**	75 (2.2)	77 (2.99)	2573	60 (1.8)	112 (3.7)	3050
**Maternal marital status**			2887			3433
Never married	527 (15.1)	680 (23.6)		467 (13.7)	853 (24.9)	
Widowed	<5	6 (0.2)		8 (0.2)	<5	
Divorced	110 (3.2)	143 (5.0)		118 (3.5)	188 (5.5)	
Separated	50 (1.4)	58 (2.0)		36 (1.1)	70 (2.0)	
1st Marriage	2581 (73.9)	1832 (63.5)		2544 (74.8)	2086 (60.8)	
Marriage 2 or 3	224 (6.4)	168 (5.8)		229 (6.7)	234 (6.8)	
**Household social class †**			2337			2758
Professional	517 (15.8)	213 (9.1)		532 (16.7)	275 (10.0)	
Managerial & Technical	1463 (44.6)	893 (38.2)		1404 (44.1)	1062 (38.5)	
Non-Manual	793 (24.2)	637 (27.3)		792 (24.9)	723 (26.2)	
Manual	343 (10.5)	411 (17.6)		328 (10.3)	479 (17.4)	
Part Skilled & Unskilled	161 (4.9)	183 (7.8)		128 (4.0)	219 (7.9)	
**Maternal education**			2605			3088
Less than O level	761 (22.2)	1025 (39.4)		724 (21.6)	1238 (40.1)	
O level	1166 (34.1)	920 (35.3)		1195 (35.7)	1036 (33.6)	
A level	926 (27.1)	435 (16.7)		883 (26.4)	550 (17.8)	
Degree or above	569 (16.6)	225 (8.6)		548 (16.4)	264 (8.6)	
**Mother's Partner's highest educational qualification**			2479			2915
Less than O level	965 (28.9)	1056 (42.6)		863 (26.5)	1258 (43.2)	
O level	717 (21.5)	501 (20.2)		724 (22.2)	609 (20.9)	
A level	916 (27.5)	625 (25.2)		918 (28.2)	657 (22.5)	
Degree or Above	737 (22.1)	297 (12.0)		750 (23.0)	391 (13.4)	
**Maternal smoking during pregnancy**	660 (18.9)	891 (30.5)	2992	657 (19.2)	1126 (32.8)	3438
	Mean (SD)	***Mean (SD)***		***Mean (SD)***	***Mean (SD)***	
**Birthweight (g)**	3370 (512)	3283 (579)	3158	3469 (578)	3395 (631)	3723
**Gestational age (weeks)**	39.6 (1.8)	39.2 (2.9)	3230	39.3 (1.9)	39.0 (3.0)	3812
**Maternal age (years)**	28.8 (4.6)	26.8 (5.0)	3198	29.1 (4.7)	27.1 (5.1)	3778
**Maternal pre-pregnancy BMI (kg/m²)**	22.8 (3.6)	23.1 (4.1)	2448	22.9 (3.8)	23.0 (3.9)	2845

*Represents participants included in models of 144 concentrations with data at all four time points; exact denominators in this table will vary due to missing data for characteristics which were not required for inclusion in analyses.

O'Keeffe LM, *et al. Heart* 2023; 109:674–685. doi: 10.1136/heartjnl-2022-321347

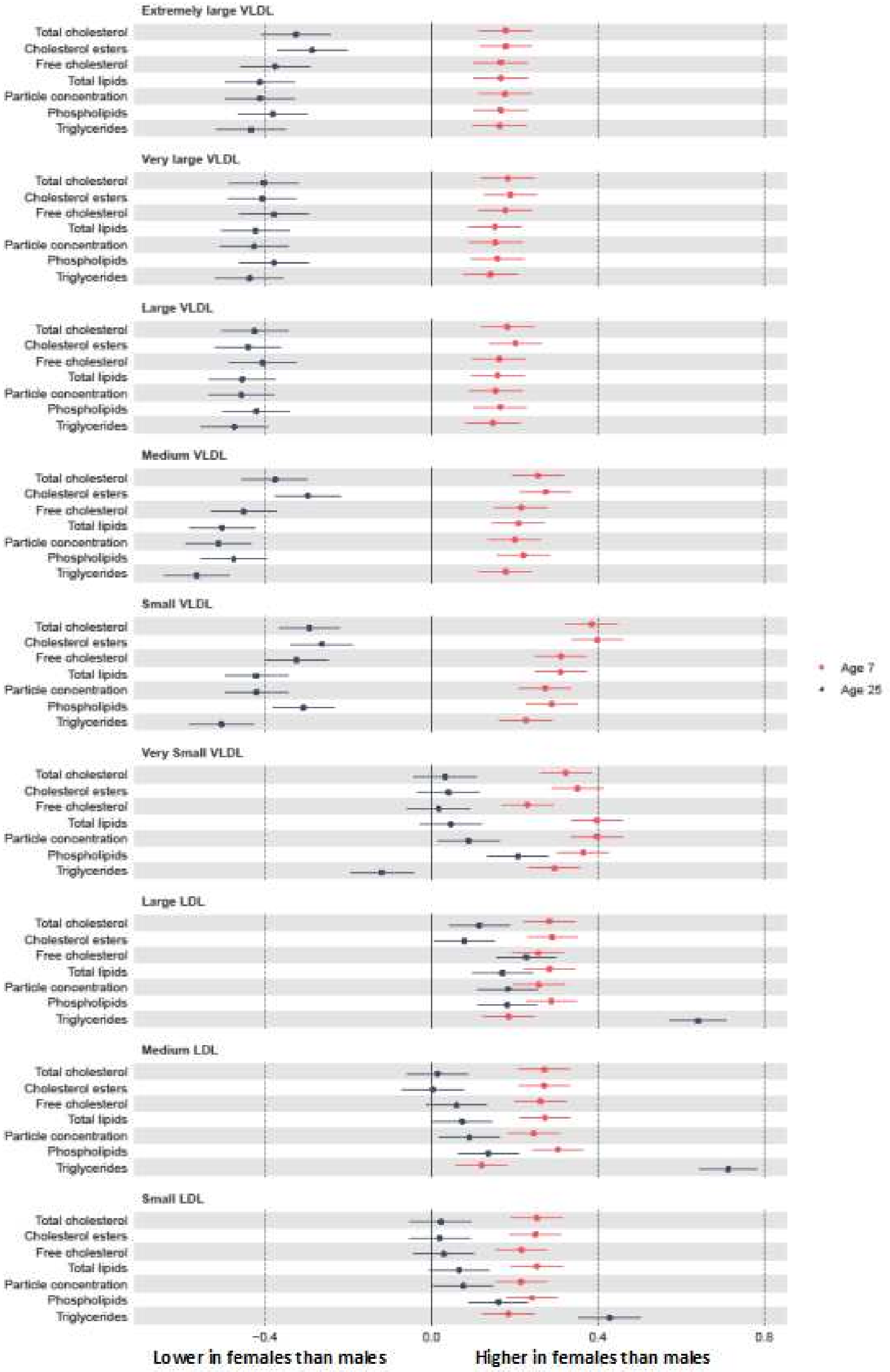

S1 Figure Mean sex difference in VLDL and LDL lipoprotein concentrations in SD units at 7y and 25y, estimated from multilevel models weighted by the probability of inclusion in analysis. Legend: LDL, low-density lipoprotein; VLDL, very-low-density lipoprotein. Differences shown are for females compared with males.

O'Keeffe LM, *et al. Heart* 2023; 109:674–685. doi: 10.1136/heartjnl-2022-321347

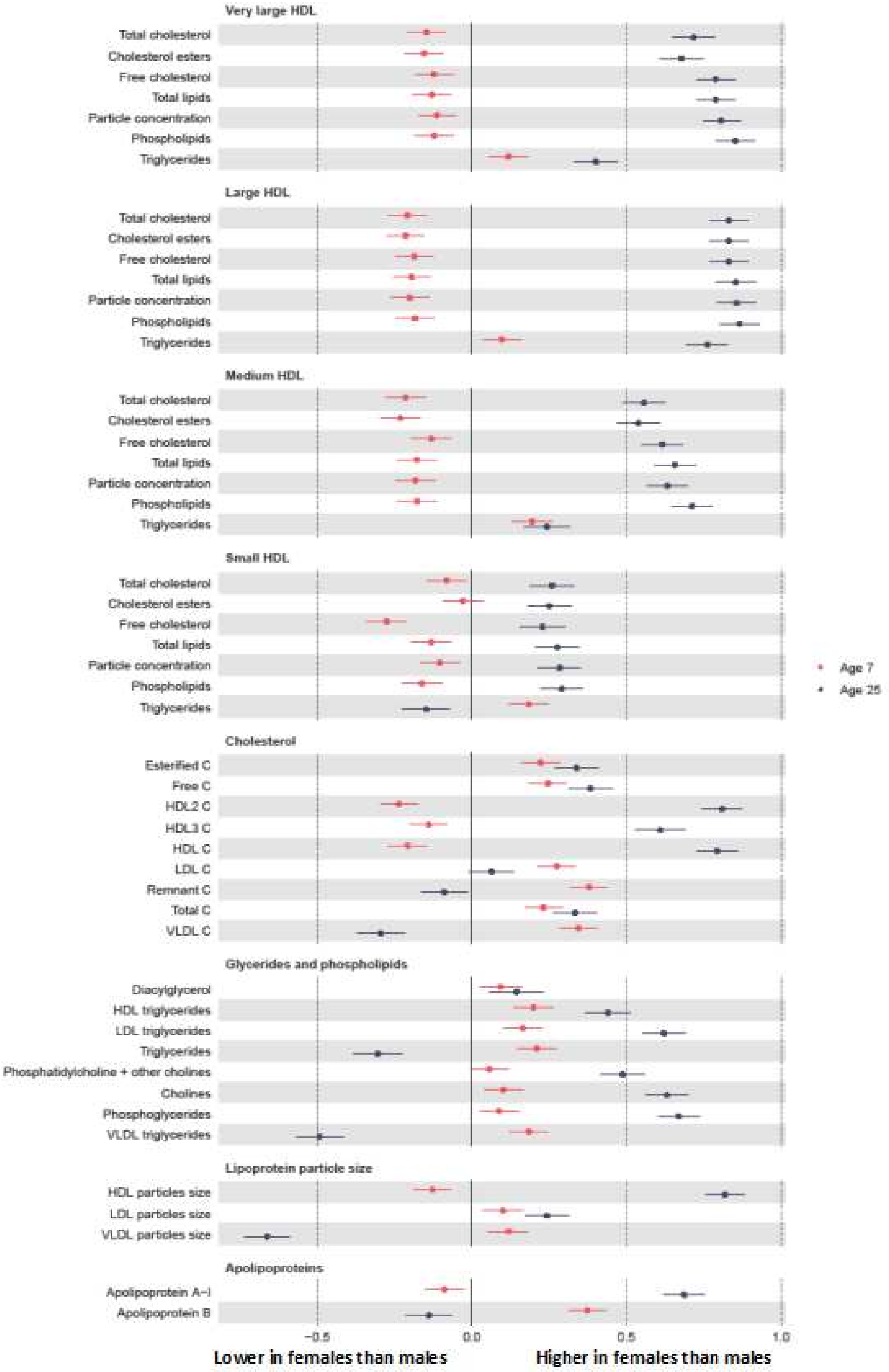

S2 Figure Mean sex difference in lipid concentrations in SD units at 7y and 25y, estimated from multilevel models weighted by the probability of inclusion in analysis. Legend: HDL, high-density lipoprotein; LDL, low-density lipoprotein; VLDL, very-low-density lipoprotein. Differences shown are for females compared with males. Note that diacylglycerol is only measured up to 18y.

O'Keeffe LM, *et al. Heart* 2023; 109:674–685. doi: 10.1136/heartjnl-2022-321347

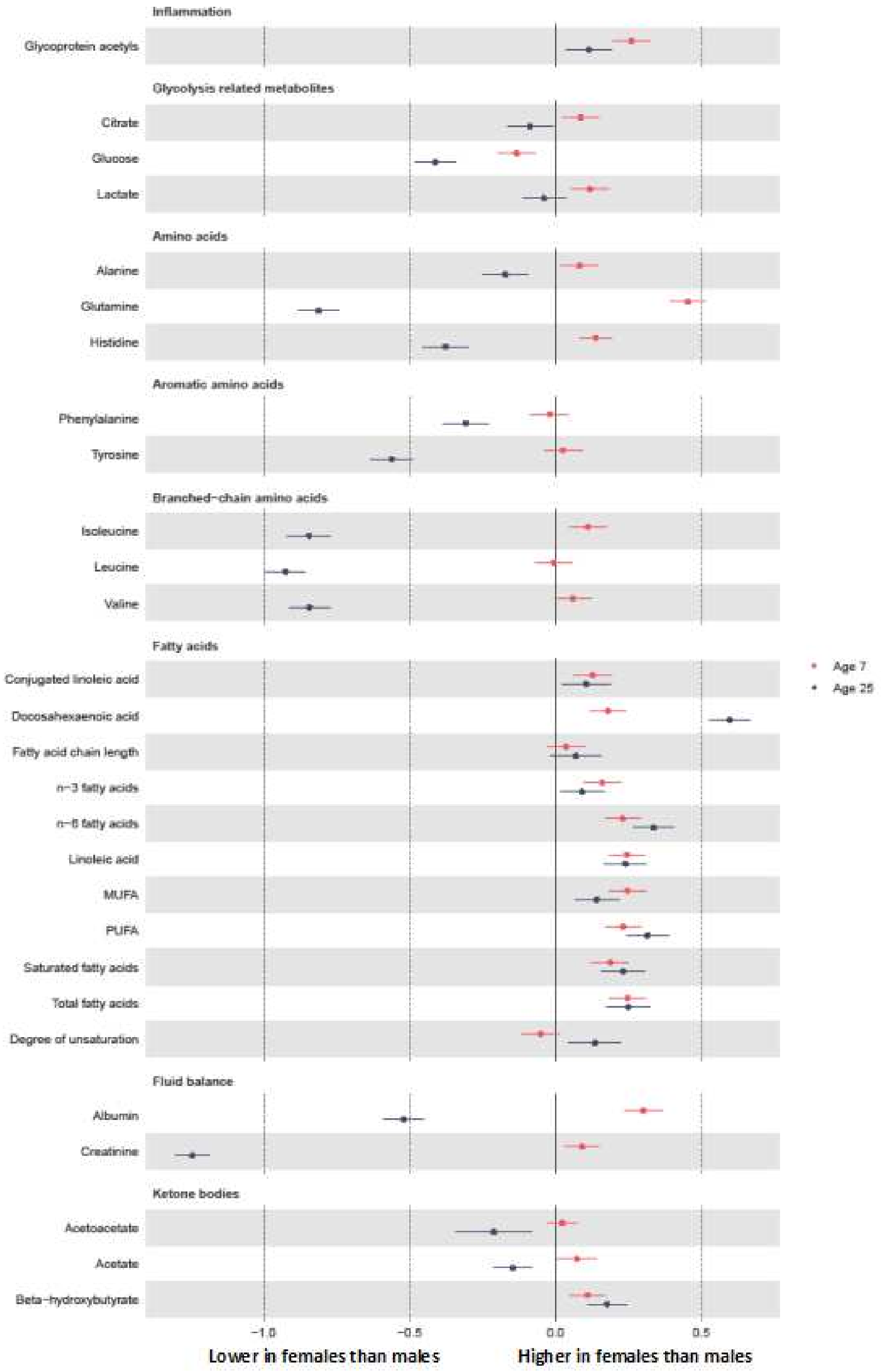

S3 Figure Mean sex difference in other trait concentrations in SD units at 7y and 25y, estimated from multilevel models weighted by the probability of inclusion in analysis. Legend: MUFA, monounsaturated fatty acids; PUFA, polyunsaturated fatty acids. Differences shown are for females compared with males. Note that conjugated linoleic acid, fatty acid chain length and estimated degree of unsaturation are only measured up to 18y.

O'Keeffe LM, *et al. Heart* 2023; 109:674–685. doi: 10.1136/heartjnl-2022-321347

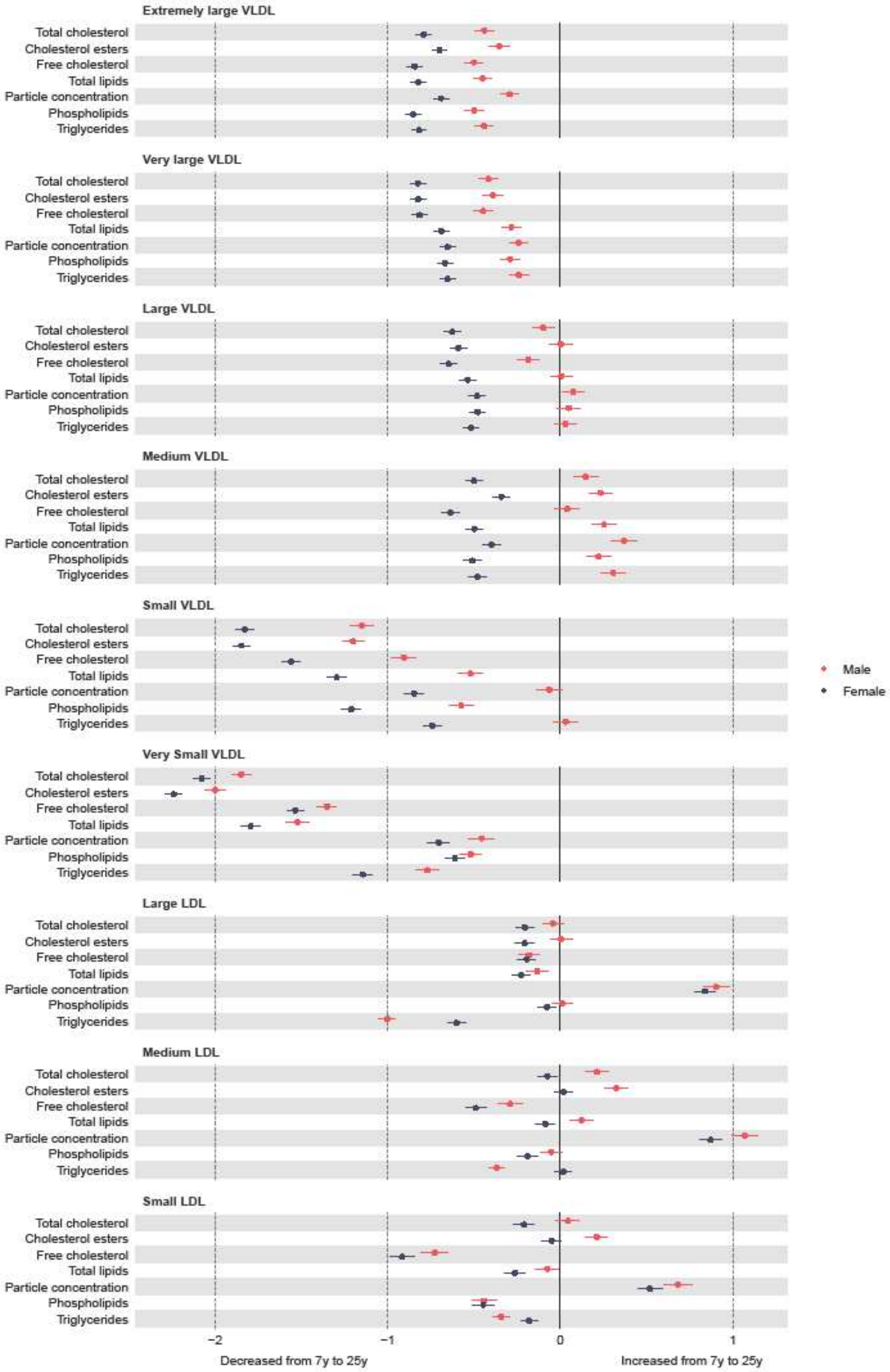

S4 Figure Mean sex-specific change in VLDL and LDL lipoprotein concentrations in SD units (standardised using sex-specific SDs) from 7y to 25y, estimated from multilevel models. Legend: LDL, low-density lipoprotein; VLDL, very-low-density lipoprotein.

O'Keeffe LM, *et al. Heart* 2023; 109:674–685. doi: 10.1136/heartjnl-2022-321347

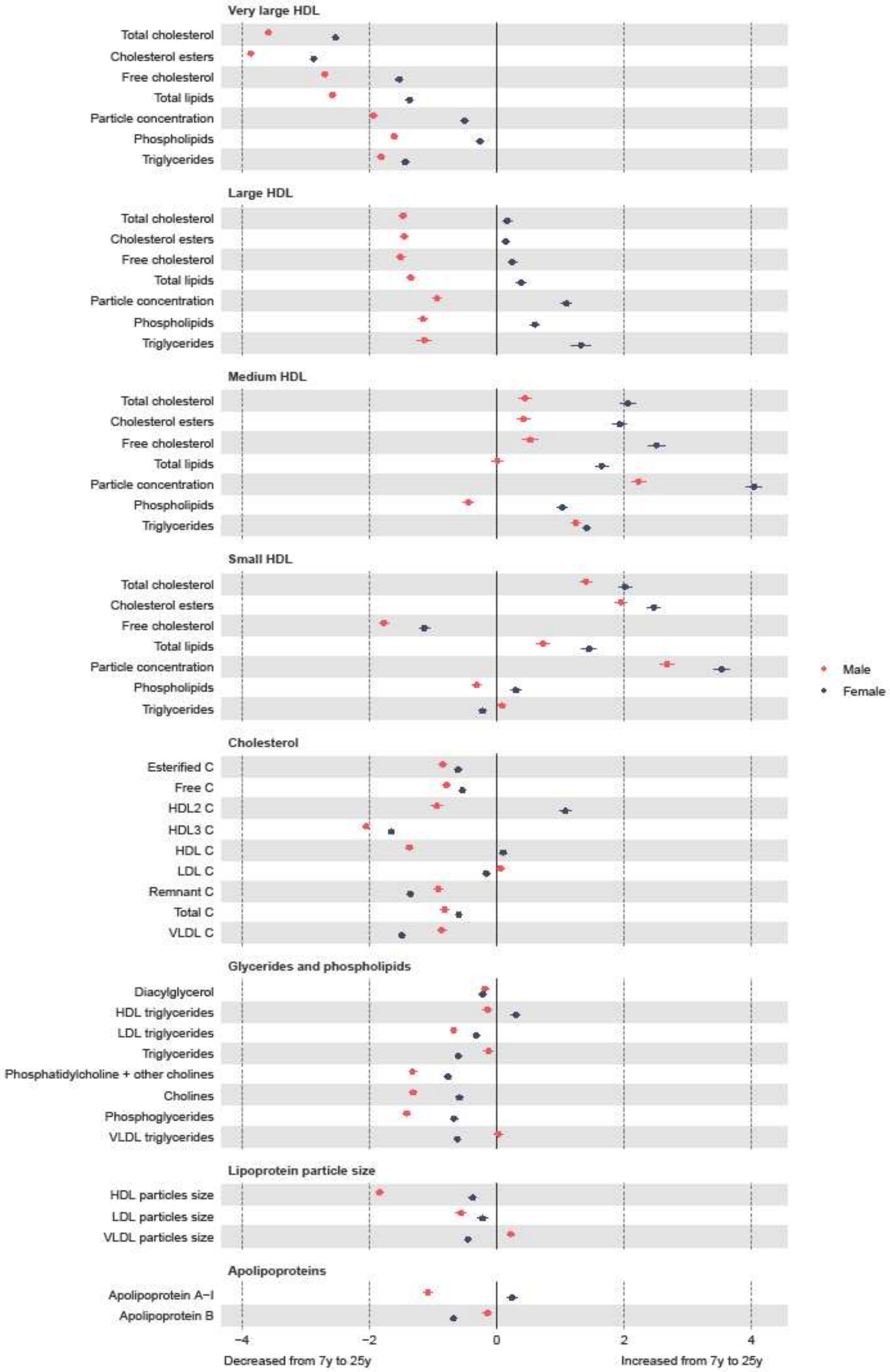

S5 Figure Mean sex-specific change in lipid concentrations in SD units (standardised using sex-specific SDs) from 7y to 25y, estimated from multilevel models. Legend: HDL, high-density lipoprotein; LDL, low-density lipoprotein; VLDL, very-low-density lipoprotein. Note that diacylglycerol is only measured up to 18y.

O'Keeffe LM, *et al. Heart* 2023; 109:674–685. doi: 10.1136/heartjnl-2022-321347

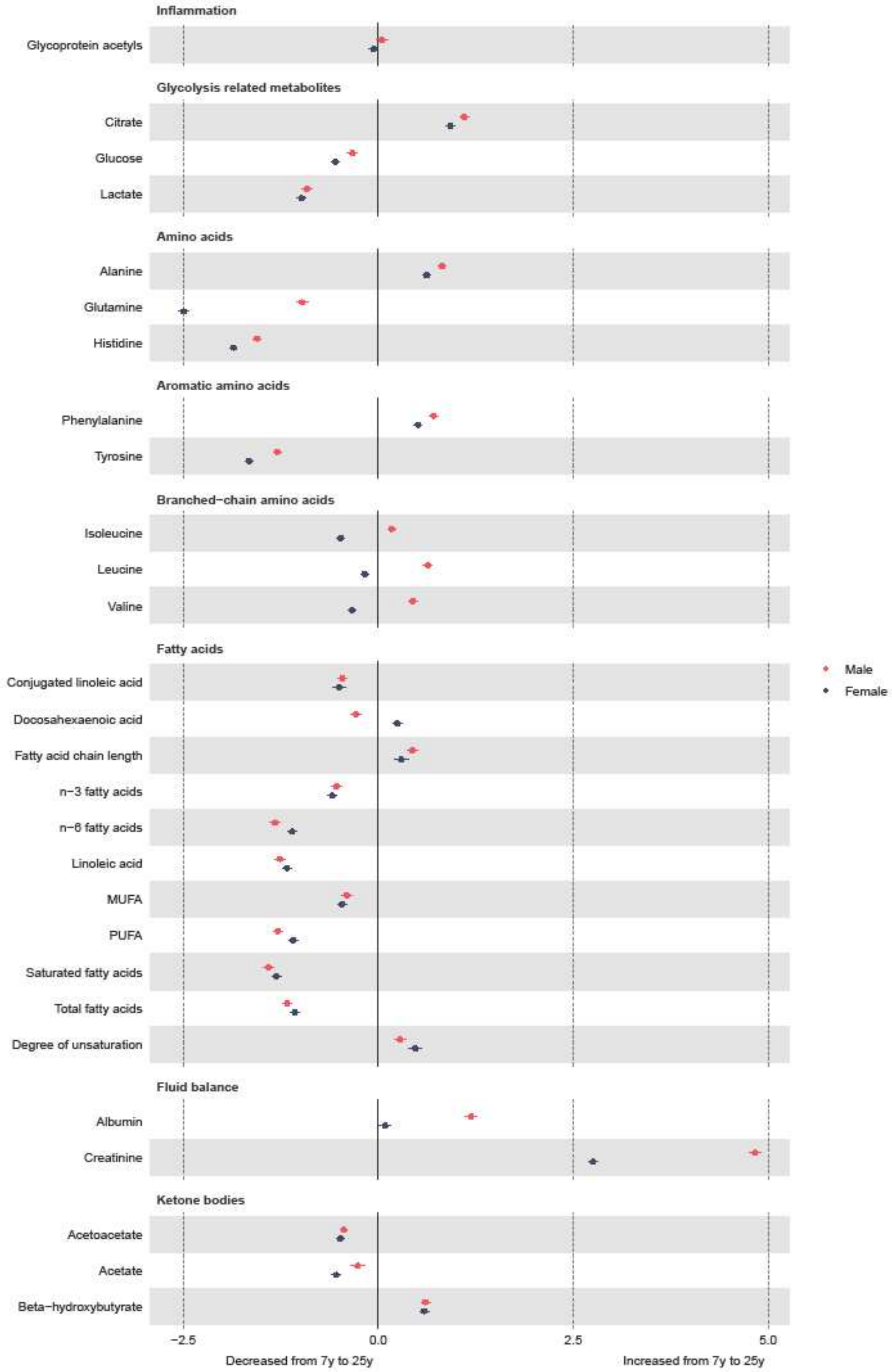

S6 Figure Mean sex-specific change in other trait concentrations in SD units (standardised using sex-specific SDs) from 7y to 25y, estimated from multilevel models. Legend: MUFA, monounsaturated fatty acids; PUFA, polyunsaturated fatty acids. Note that conjugated linoleic acid, fatty acid chain length and estimated degree of unsaturation are only measured up to 18y.

O'Keeffe LM, *et al. Heart* 2023; 109:674–685. doi: 10.1136/heartjnl-2022-321347

References

1. Hughes DA, Taylor KA, McBride N, et al. metaboprep: an R package for pre-analysis data description and processing. 2021

O'Keeffe LM, *et al. Heart* 2023; 109:674–685. doi: 10.1136/heartjnl-2022-321347
